# Both k-core percolation and directed graph analysis revealed succession and transition of voxels’ spatiotemporal progress on dynamic correlation resting-state fMRI

**DOI:** 10.3389/fnhum.2025.1543854

**Published:** 2025-04-16

**Authors:** Dong Soo Lee, Hyun Joo Kim, Youngmin Huh, Yeon Koo Kang, Wonseok Whi, Hyekyoung Lee, Hyejin Kang

**Affiliations:** ^1^Medical Science and Engineering, School of Convergence Science and Technology, Pohang University of Science and Technology, Pohang, Republic of Korea; ^2^Nuclear Medicine, College of Medicine, Seoul National University, Seoul, Republic of Korea; ^3^Nuclear Medicine, Korea University Anam Hospital, Seoul, Republic of Korea; ^4^Biomedical Research Institute, Seoul National University Hospital, Seoul, Republic of Korea

**Keywords:** graph node hierarchy, afferent node capacity, information flow, state transition, resting-state fMRI, k-core percolation, volume entropy

## Abstract

**Introduction:**

Voxel hierarchy on dynamic brain graphs is produced by k-core percolation on functional dynamic amplitude correlation of resting-state fMRI.

**Methods:**

Directed graphs and their afferent/efferent capacities are produced by Markov modeling of the universal cover of undirected graphs simultaneously with the calculation of volume entropy. Using these methods, state stationarity was tested for resting-state positive and unsigned negative brain graphs separately on sliding-window representation. The spatiotemporal progress of voxels was visualized and quantified.

**Results and discussion:**

The voxel hierarchy of positive graphs revealed abrupt changes in coreness k (k-core) and maximum k-core (kmaxcore) voxels on animation maps representing state transitions interspersed among the succession. Afferent voxel capacities of the positive graphs revealed transient modules composed of dominant voxels and independent components as well as their exchanges compatible with transitions. Moreover, this voxel hierarchy and afferent capacity corroborated each other only on the positive directed functional connectivity graphs but not on the unsigned negative graphs. The Spatiotemporal progression of voxels on positive dynamic graphs constructed a hierarchy by k-core percolation and afferent information flow by volume entropy and directed graph methods. We disclosed the non-stationarity and its temporal progress pattern at rest, accompanied by diverse resting-state transitions on resting-state fMRI graphs in normal human subjects.

## Introduction

The spatiotemporal trajectory of neurons is structured to represent population codes that support behavior and the underlying mental processes in humans and animals. The limited spatial and temporal resolution of measuring devices such as functional magnetic resonance imaging (fMRI) has obstructed the investigation of human mental processes, which has been further complicated by computing resources. The resting-state fMRI might have been widely used clinically, especially considering its ready availability but not, for assessing mental states’ fluctuation or transition in humans (Chang and Glover, 2010; Handwerker et al., 2012; Hutchison et al., 2013; Allen et al., 2014; Huh et al., 2022). Another barrier to ready use was the technological one, where we had to use regions of interest or principal components as the analytic units, instead of voxels.

However, improved brain imaging devices and pre- and post-processing technology with enhanced computing resources (Vidaurre et al., 2017; O'Neill et al., 2018; Vidaurre et al., 2018; Deco et al., 2021; Vidaurre, 2021; Vidaurre et al., 2021) now allow voxel-based investigations, which are currently the smallest macrocomplexes of neurons and allies. There have been attempts to investigate voxel level with dynamic functional structures of time-varying measures, although voxels themselves still contain an average of 100,000 neurons/mm^3^ (Vidaurre et al., 2017; Zhang et al., 2018; Huh et al., 2022; Matkovic et al., 2023). For instance, the time-varying features of instantaneous amplitude correlation, reduced to a few principal components, were successfully correlated with dynamic resting states and even personality traits based on their elucidation (Vidaurre et al., 2017). We now need to expand these pilot investigations to refined intervoxel studies while respecting the identities of voxels and propose proper measures to represent human resting states and their quantified contributions. The literature on intervoxel amplitude correlation has already revealed an equal prevalence of amplitude correlation and anticorrelation on voxel-based approaches (Wu et al., 2013; Huh et al., 2022; Fransson and Strindberg, 2023), unlike previous investigations that observed mostly positive region-based correlations (Wong et al., 2012; Wu et al., 2013; Parente and Colosimo, 2020; Noro et al., 2022). Resting-state fMRI yields the output of two immiscible graphs, equally propense graphs of correlation and anticorrelation, awaiting novel approaches to understanding how voxels compete and collaborate in composing resting states in humans, both in dynamic plots and in the static state (Huh et al., 2022).

The parameters of functional brain graphs had been either related to graph theory or classical many-body pairwise embedding technology. These methods had setbacks in terms of their implicit assumption that graphs are reducible to principal components (Vidaurre et al., 2017; O'Neill et al., 2018; Vidaurre et al., 2018; Deco et al., 2021; Vidaurre, 2021; Vidaurre et al., 2021) and that understanding the parameter and its distribution (i.e., degree and degree distribution for the rich-club coefficient) would be suitable for elucidating the hidden structure of intervoxel interactions (Zhou and Modragon, 2004; Colizza et al., 2006; Kim and Min, 2020). They are correct in terms of searching for the global characteristics of graphs or networks. However, investigators implicitly ignored the identities of voxels. What if voxels, not neurons as voxels, are already a macrostructure of neuron–glia–vessel complexes, and 1 min is enough time for making ensembles, calculating, and acting independently to release emergent behaviors with higher-order interactions? We attempted to restore the identity of voxels by calculating their characteristic contributions to the functional hierarchy (Huh et al., 2022) and in-degree (i.e., afferent to the nodes) information flow capacity in dynamic functional graphs.

Another limitation of mainstream brain connectivity investigations is their inability to produce dynamic representations of functional brain graphs (Chang and Glover, 2010; Handwerker et al., 2012; Hutchison et al., 2013; Allen et al., 2014; Huh et al., 2022) and/or their ignorance of the possibility to make directed weighted graphs using pairwise undirected observables (such as amplitude correlations) of dynamic functional brain graphs (Wu et al., 2013; Khazaee et al., 2017; Razi et al., 2017). We recently explored these uncharted methods and introduced the following schemes of investigation for using resting-state fMRI to characterize voxel hierarchy (Huh et al., 2022) and afferent or efferent node capacity, which are intended to represent the dynamic functional graphs of mental-state fluctuation and transition in humans.

**Figure 1:**
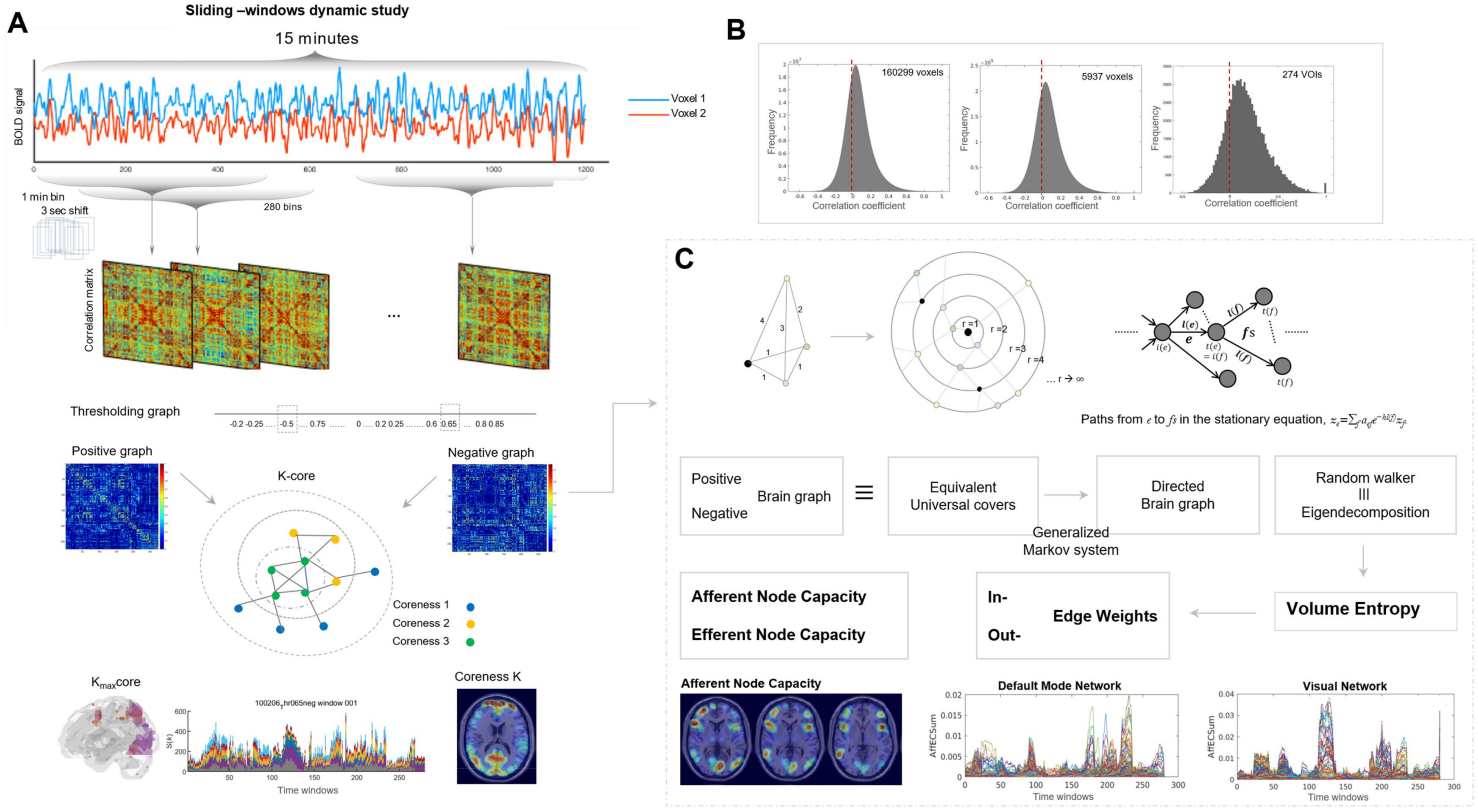
Schematic representation of methodological procedure. **(A)** Sliding-window representation of resting-state fMRI and its spatiotemporal visualization in matrix and MRI-overlaid animation plots. Resting-state fMRI acquired 15 min, 0.72 s for each frame were converted to 1-min time bin, 3 s of time-bin shifts, and then 280 time bin of timepoints were obtained for the HCP cohort. For 180 individuals from the Human Connectome Project, from 1,200 frames for 2 mm × 2 mm × 2 mm fMRI images, 280 time bins were derived, and the downsampled total number of voxels was 5,937 voxels for 6 mm × 6 mm × 6 mm and 1,489 voxels for 10 mm × 10 mm × 10 mm. The former was used for k-core percolation, and the latter was used for directed graph construction. Brain graphs consisted of 280 time-bin images of 5,937 × 5,937 (or 1,489 × 1,489) matrices or 280 time-bin images of 36-slices brain MRI-overlaid k-core map plots or afferent/efferent node capacity map plots. For both images, the final output in the form of MRI-overlaid brain images was in audio–video interleave (AVI) files and best viewed with animation play software of any kind. **(B)** Observed pairwise intervoxel correlations of brain graphs of the Human Connectome Project using the initially reconstructed 2 mm × 2 mm × 2 mm resolution (160,299 voxels), 6 mm × 6 mm × 6 mm resolution (5,937 voxels), and 274 anatomical regions of interest. Propensity of intervoxel correlations derived from the 160,999 voxels (2 mm × 2 mm × 2 mm) with 12.9 billion undirected edges, 5,937 voxels (6 mm × 6 mm × 6 mm) with 17.6 million undirected edges, and 274 anatomical volumes-of-interest (VOIs) with 37 K edges. Positive shift (less amounts of negative-valued edges than positive-valued edges) was already there but in small fraction in the initial 2 mm × 2 mm × 2 mm or 6 mm × 6 mm × 6 mm resolution brain graph, however, in brain graphs with 274 VOIs, negative correlations ranges from −0.3 to 0 and the area was just quarter of positive correlations. **(C)** Scheme to estimate volume entropy and afferent and efferent node capacities by making directed weighted graphs from the observed pairwise intervoxel undirected amplitude correlations after thresholding separately on positive and unsigned negative graphs. The graphs we acquired from resting-state fMRI brain imaging were, after thresholding, put into the calculation of volume entropy reported by Lee et al. (2019) and Lim (2008). An undirected graph was transformed to a universal cover, which is equivalent to the brain graph of interest. By modeling the graph geodesic configuration search with a generalized Markov system, the edges of the universal cover were traced by a random walker on the surface (N-1 dimensional) of the N-dimensional ball to infinity. The random walk is asymptotically yielding a volume bounded by the maximum and minimum (Lim, 2008). This volume was equivalent to the geodesic topological invariant of (N-1) dimensional surface of an N-ball, called volume entropy. While volume entropy defines the total information flow of the graph of interest, the edge lengths of the universal cover at the limit to infinity for the radius of the N-ball, once cropped as a matrix, yielded an edge matrix. As we used 1,489 nodes, 1,107,816 undirected edge-matrix before modeling, it was supposed to become a 2,217,121-element asymmetric matrix. The equivalence of a random walker on the universal cover in edge-matrix and eigen decomposition was adopted to calculate edge matrix and volume entropy (Lee et al., 2019), and MATLAB function eigs was used for the exact calculation. Finally, we could produce the edge length (capacity) matrix, and thus we summed up all the incoming edge weights to a node, to call it afferent node capacity, and all the outgoing edge weights from that node, to call it efferent node capacity (Ha et al., 2020). The final products of afferent/efferent node capacity were the normalized ones, and thus timepoint plots could be drawn for afferent and efferent node capacities, separately for positive and negative graphs of an individual. Top-right path diagram in Figure 1C; Reproduced from Lee et al., Scientific Reports, 2019, licensed under CC BY 4.0.

In this study, we assumed that (1) dynamic functional brain graphs derived from resting-state fMRI represent the fluctuating and sometimes transitioning brain states of human minds at resting state (Hutchison et al., 2013; Mooneyham et al., 2017; Gonzalez-Castillo et al., 2021); (2) sliding-window time binning of resting-state fMRI (Figure 1) enables visualization of continuous (non-explosive) temporal changes in composition (Hindriks et al., 2016) with the waxing and waning voxel behavior over time; (3) waveforms of each voxel, once observed pairwise, act as the simplest representation of their higher-order interaction (Lambiotte et al., 2019; Battiston et al., 2020), which might reveal the inherent characteristics of many-body intervoxel interactions (Khona and Fiete, 2022; Bollt et al., 2023); and (4) the above pairwise-observed amplitude correlations are the sum of signals (functional connectomic dynamics) and redundancy (including inherent and measurement-related error/noise) (Khona and Fiete, 2022; Varley et al., 2023). The first of the above assumptions is not refutable, meaning that this investigation cannot prove or refute it (Whi et al., 2022b); however, the remaining three are to be corroborated or partially proven for some measures or disproved for others by our study results.

Among graph-based methods, percolation has been successfully introduced to reveal hierarchical organization of resting-state fMRI connectivity in a mouse model (Bardella et al., 2016) and in humans (Bordier et al., 2017; Mastrandrea et al., 2017), to disclose disease-related differences (Mastrandrea et al., 2021). Percolation analysis on the multi-unit array data in primates revealed a hierarchical structure of neuronal interaction in movement control or motor inhibition (Bardella et al., 2020; Bardella et al., 2024). Hierarchical consideration of edges in the structure of individual graphs has been shown to benefit percolation for optimal thresholding (Bordier et al., 2017), remove weaker edges for sparsification (Nicolini and Bifone, 2016), or maintain the scale-free structure of functional graphs by removing redundancy (Huh et al., 2022). Recently, graph neural networks (Cui et al., 2023; Li et al., 2023) have enabled the extraction of spatial features in a generative deep learning especially using transformer and attention methods (Kim et al., 2021; Kan et al., 2022), primarily on static resting-state fMRI. The spatiotemporal progression of dynamic spatial and temporal features can be analyzed using methods such as temporal convolution networks and spatial self-attention blocks (Thapaliya et al., 2025a; Thapaliya et al., 2025b). Until now, most of the methods and the packages (Cui et al., 2023; Thapaliya et al., 2025b) allowed the data input in hundreds of regions but not in voxels. Several thousand voxels, now available as inputs made possible by this study, can be used to reveal spatiotemporal characteristics of mental states on resting-state fMRI.

Based on our preliminary research, described in previous studies (Huh et al., 2022; Whi et al., 2022b), the threshold range of dynamic brain graphs was set to guarantee scale-free degree distribution (distribution freedom) while cropping a large number of voxels. This thresholding was later found to be necessary for revealing the state transition of maximum coreness k (k-core) voxels, but not for discovering the abrupt module exchange of afferent node capacity on dynamic-directed weighted graphs. The sliding-window method (Huh et al., 2022; Whi et al., 2022b) was adopted to find intervoxel amplitude correlations separately for positive and unsigned negative graphs to determine intervoxel similarity per time bin for further analysis of (1) the production of k-core animation maps and corollary plots such as glass brain, flag plots, and timepoint plots with trajectory tracing and (2) afferent and efferent node capacity maps on animation. Upon varying thresholds using an exemplary case and the same threshold for all the time bins of individuals from the Human Connectome Project (HCP) (Glasser et al., 2013; Van Essen et al., 2013; Elam et al., 2021), the total sum of k-core voxels or temporal progression of afferent node capacity were compared with graphs’ sums of the number of edges, and the threshold effect was determined based on the following findings of this investigation.

Thus, in this investigation, using resting-state fMRI data from the HCP (Glasser et al., 2013; Van Essen et al., 2013; Elam et al., 2021), we performed k-core percolation (Azimi-Tafreshi et al., 2019; Kong et al., 2019; Whi et al., 2022b) for dynamic intervoxel amplitude correlations and calculations of volume entropy, estimating afferent/efferent node capacity (Lee et al., 2019; Ha et al., 2020). We investigated whether time-binned figures of k-core and maximum k-core (k_max_core) voxels produced from sliding-window method. (Huh et al., 2022; Whi et al., 2022b) prepared using a sliding-window approach would successfully display dynamic spatiotemporal changes in state transitions involving the participating components of k-core and k_max_core. Additionally, we compared whether the state transitions in the voxel hierarchy study were similarly represented on information-flow maps of in-degree (afferent) and out-degree (efferent) voxel capacities for both positive and negative graphs. Finally, we investigated whether the voxels themselves could be traced on the quantified maps in animation as well as on timepoint plots, which may enable identifying each voxel on the temporal axis by its characteristic values.

## Methods

### Data preprocessing

We downloaded resting-state fMRI (rsfMRI) data from the HCP.^1^ We selected 180 participants aged 22 to 36 years without any significant history of psychiatric disorders or neurological or cardiovascular disease (Van Essen et al., 2013; Elam et al., 2021). We used resting-state fMRI FIX-Denoised preprocessed data (Glasser et al., 2013) and performed further preprocessing, including smoothing with a 6-mm full-width at half-maximum Gaussian kernel and bandpass filtering (0.01–0.1 Hz). Next, we downsampled the data from 91 mm × 109 mm × 91 mm voxels to 2 mm × 2 mm × 2 mm voxels, and then from 31 mm × 37 mm × 31 mm voxels to 6 mm × 6 mm × 6 mm voxels, which reduced the computational load. We applied a mask to exclude voxels that did not belong to the brain, resulting in 5,937 voxels for k-core percolation. For the volume entropy calculation and directed graph composition, another downsampling with a 10 mm × 10 mm × 10 mm voxel resulted in 1,489 voxels.

### Independent component analysis

We performed independent component analysis (ICA) to identify independent components (ICs), that is, resting-state networks, using multivariate exploratory linear decomposition into independent components (MELODIC) (Beckmann et al., 2009). We obtained spatial maps of ICs and applied a threshold (*Z* > 6) to generate binary masks. In this study, we included seven ICs: the default mode network (DMN), salience network (SN), dorsal attention network (DAN), central executive network (CEN), sensorimotor network (SMN), auditory network (AN), and visual network (VN).

### Dynamic data analysis: sliding-window analysis

For the HCP data, we used sliding-window analysis to investigate the non-stationary and time-dependent dynamics of the brain. The window size was set close to 1 min (84 volumes, 60.48 s) with a shift of 4 volumes (2.88 s), resulting in 280 windows. A connectivity matrix of each window was calculated to conduct k-core percolation. We implemented k-core percolation for each connectivity matrix after applying the threshold, which ensures the scale-free network, to generate a binary matrix. Edges with values greater than 0.65 in positive graphs and 0.5 in negative graphs are assigned a value of 1; otherwise, they are assigned a value of 0.

### k-core percolation

We conducted k*-*core percolation to investigate the core structure of an individual’s functional brain network (Azimi-Tafreshi et al., 2019; Whi et al., 2022b). Metaphorically, this method peels away the layers of the network, much like peeling an onion. This procedure first removes nodes of degree 1 (*k* = 1). As nodes are removed, the degree of the remaining nodes also changes. Some nodes, whose degrees were not initially 1, are eventually removed if they meet the removal criteria. The procedure is performed recursively by incrementing k by 1 until no further processing is possible. A subgraph, known as the k-core, is obtained by removing all nodes with degrees less than k. The last surviving core was called the k_max_core. After k-core percolation was performed on each subject’s data, we classified k_max_core voxels using IC maps.

### Volume entropy calculation and construction of directed weighted graphs

During the volume entropy calculation, we modeled the functional brain graphs to have directedness using a generalized Markov system on universal covers of the undirected graph matrices. Matrix elements were detected via pairwise correlations of the voxels’ waveforms on resting-state fMRI. Universal coverage allowed one-directional random walkers to traverse all possible paths from any node to infinity, yielding an N-dimensional ball of infinite radius. The topological dynamics representing the capacity of information flow over the graphs were proven to have an asymptotic invariant specific to the graph. These intermediary edge matrices were supposed to reveal the in- and out-flow capacity of information via every edge from the standpoint of the information flow along the brain graphs (Lee et al., 2019). We refer to the in-flow capacity of certain nodes as the afferent node (voxel–node) capacity and the out-flow capacity as the efferent node capacity, as described in our previous study (Ha et al., 2020). Thus, edge length (or distance) derived from pairwise intervoxel amplitude correlation was used to define the hidden directed functional brain network.

As the N-dimensional ball of the universal cover expanded toward infinity with an increasing radius, the geodesic sum of the distance traveled by the random walker was modeled using a generalized Markov system. Eigendecomposition replaced the random walker model and yielded edge capacity matrices. A total of 10 mm × 10 mm × 10 mm downsampling resulted in a maximum of 1,489 × 1,489 edges, and after thresholding with the same criteria as for the 6 mm × 6 mm × 6 mm image data, approximately 2 million (incoming and outgoing) edges remained, allowing for the creation of 2,000,000 × 2,000,000 matrices for eigen decomposition. The eigs function of MATLAB^®^ 2024b (The MathWorks, Inc., Natick, MA, USA) was used to calculate edge matrices via Krylov–Schur decomposition, and then volume entropy was calculated in 2–3 h of computing time per time bin. This led to the completion of the calculation of edge matrices and volume entropy per individual within weeks (4 weeks for HCP data with 280 time bins per individual).

### Data visualization

Using graphs with 5,937 voxels and 6 mm × 6 mm × 6 mm brain volume images, k-core percolation was performed. The resulting outputs were visually represented as (1) animation maps of edge-scaled k-core values overlaid on 36-slice magnetic resonance imaging (MRI) templates, (2) edge-scaled flag plots on the runs of voxels with their IC labels, (3) k_max_core stacked histograms on the runs with their IC labels, (4) edge-scaled k-core value plots, (5) animation glass brain lateral or transaxial images for 280 time bins of 1-min duration (84 acquisition bins) with approximately 3 s (4 bins = 2.88 s) shifting from 1,200 acquisition bins (0.72 s/bin) of the HCP datasets. All these maps are presented as positive and unsigned-negative brain graphs. The total number of edges counted after thresholding was used to normalize (divide) k-core voxel values. These edge-scaled k-core values were used for animation plots, flag plots, and voxel trajectory timepoint plots per IC (voxel/IC trajectory timepoint plots).

Using graphs with 1,489 voxels and 10 mm × 10 mm × 10 mm brain volume images, the volume entropy was calculated again for positive and negative graphs, while simultaneously producing directed weighted graphs for each type of graph. The resulting outputs were visually represented as (1) animation maps of afferent or efferent node capacity overlaid on MRI templates and (2) afferent and efferent timepoint plots representing each voxel’s trajectory and their collective picture separately according to their belonging to ICs (DMN, SN, DAN, CEN, SMN, AN, and VN) and left cerebellum (L_Cbl), right cerebellum (R_Cbl), vermis (V), and the unclassified (Unc).

## Results

### Effect of thresholds on the number of edges, the k-core voxel, and the afferent voxel capacity

For the cohort of 180 individuals from the HCP [with 280 time bins each with sliding-window methods (Figure 1)], to crop the giant component including at least 85% of voxels but having a guaranteed scale-free (distribution-free) voxel degree distribution, thresholds were set to 0.65 for the amplitude correlation for positive graphs and 0.50 for negative graphs. Among these individuals, one exemplary case was selected to evaluate the effect of the total number of edges per time bin related to the preset thresholds. The thresholds were varied from 0.20 to 0.75 for the intervoxel correlations to construct 12 positive graphs and 12 negative graphs (Supplementary Figures S1A–D). The number of voxels in the graphs varied from 100% (*n* = 5,937) to 5% (*n* = 280) of the total voxels. Obviously, increasing thresholds decreased the number of voxels and the number of edges (Supplementary Figures S1E,F). The total number of edges showed an almost 1:1 correlation [1.09 ± 0.02 for 280 time bins with a threshold of 0.65 (voxel *n*; 5,622 − 5,174) in the positive network and 1.08 ± 0.02 for 280 time bins with a threshold of 0.5 (voxel *n*; 5,937 − 5,833) in the negative network] (Supplementary Figures S1G,H), with the total sum of k-core values per graph in both the positive and the negative graph analyses. For 180 subjects, this relationship between the total sum of edges and the total sum of k-core values was consistent across all individual and time bins, despite the use of the same thresholds of 0.65 for all positive graphs and 0.5 for all negative graphs.

**Figure 2:**
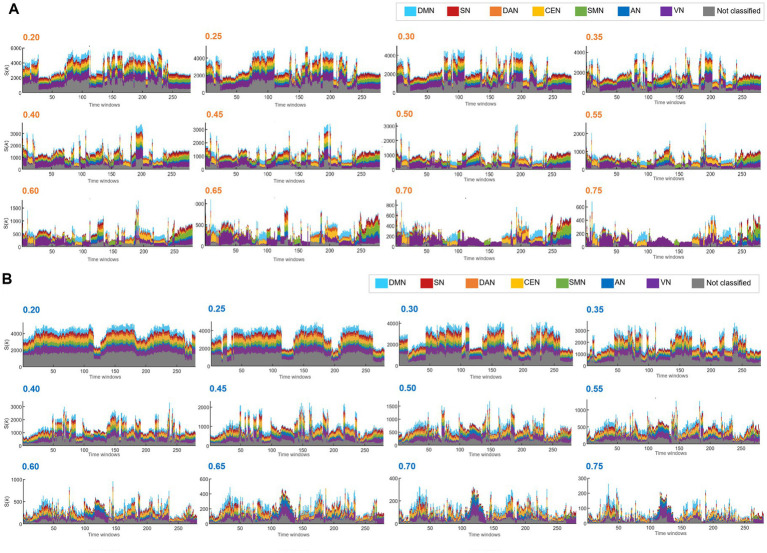
The hierarchical top tier voxels according to the varying thresholds in the positive and the unsigned negative graph. Stacked histogram k-core values in positive graphs of the case identification #100206. Each IC contained 732 voxels for DMN, 351 for SN, 363 for DAN, 682 for CEN, 483 for SMN, 289 for AN, 1,104 for VN, and 2,691 for the unclassified. **(A)** On the stacked histogram plots of k_max_core with the thresholds from 0.2 to 0.35, state transition was not found because the voxels from all the ICs participated evenly in the top tier voxels. **(B)** Stacked histograms of k-core values in unsigned negative graphs of the same case. The influence of the threshold of negative graphs was similar to that of the positive graphs, but more dramatic. With the thresholds between 0.2 and 0.3, the number of k_max_core voxels alone changed without the composition. With the thresholds of 0.35 to higher, the total k_max_core voxel numbers decreased, but the voxels/IC composition was still homogeneous. Apparent state transitions might as well show up, but with less confidence.

Based on this equivalence of the total number of edges per time bin in every individual, the k-core voxel values were divided by the total number of edges to yield edge number-scaled k-core voxel values. Thus, edge-scaling represents normalization of coreness values of voxels by the total number of edges for the graph in each time bin. The k-core voxel timepoint plots initially showed remarkable similarity in terms of the contours of collective trajectory bundles per IC, which was due to the effect of varying the total edge number per time bin (Supplementary Figure S1A). Edge-scaled k-core trajectory bundles showed gross similarity with minute differences between ICs (Supplementary Figure S1B). Scale-freedom (an almost linear decrease in the log–log plot of the degree distribution) was observed in the positive graphs with thresholds ranging from 0.55 to 0.85 and in the negative graphs with thresholds ranging from 0.50 to 0.85 (Supplementary Figures S1C,D). The voxel number of graphs allows us to disregard positive graphs with thresholds above or equal to 0.7 and negative graphs with thresholds above or equal to 0.65, as the number of voxels in these graphs was less than 85%. Notably, the voxels within the same IC exhibited heterogeneity of trajectories, contributing to the shape of the collective k-core per IC, meaning that each voxel took turns in the collective rise and fall along the time-bin axis. At a threshold above or equal to 0.4, k_max_core plots of positive graphs exhibited state transitions regardless of the number of edges in terms of the voxel composition of the k_max_core voxels and their IC belongings. On positive graphs with thresholds below 0.4, the state transition disappeared, except for the remaining fluctuations (Figure 2A). In contrast, k_max_core plots of negative graphs with thresholds of 0.35–0.55 rarely showed state transitions but depicted grossly similar fluctuating temporal progression (Figure 2B).

**Figure 3:**
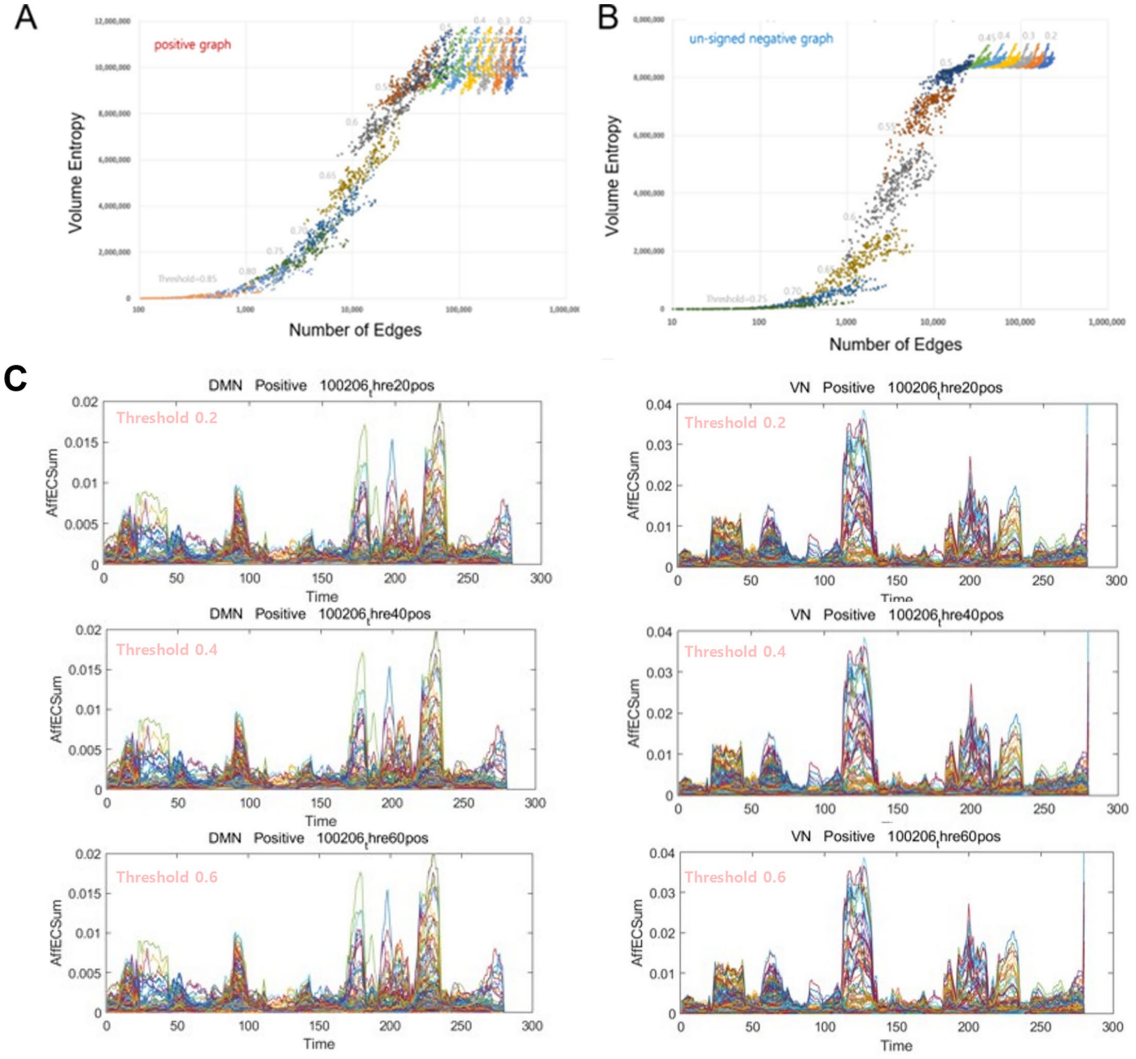
The volume entropy values, modules, and exchange patterns of the voxel/IC composition were exactly the same for the graphs with surplus edges. **(A)** In positive graphs with thresholds of 0.2, 0.25, 0.3, 0.35, 0.4, 0.45, 0.5, 0.55, 0.6, 0.65, 0.7, 0.75, 0.8, and 0.85, the volume entropy was calculated separately per threshold (*n* = 14) for each time bin (*n* = 280). The abscissas of the total number of edges were plotted logarithmically because the total number of edges ranged from thousands to millions. In these positive graphs, with thresholds equal to or lower than 0.45, the volume entropy was the same between time bins and between thresholds, irrespective of the thresholds and the corresponding numbers of edges. The volume entropy of positive graphs ranged from 9 to 12 million between time bins in graphs with thresholds of 0.45–0.2, which was larger than the range of volume entropy of negative graphs with similar thresholds. **(B)** In the negative graphs, the pattern was similar, and once the number of edges decreased to less than 8 million, the volume entropy decreased dramatically and proportionally with the number of edges. The volume entropy of negative graphs with thresholds of 0.45–0.2 ranged from 8.5 to 9 million. **(C)** Examples of voxels belonging to the DMN or to the VN showing no difference between the positive graphs with varying thresholds from 0.2 to 0.6. Notably, the contour of the formed modules, as well as the trajectories inside and the maximum height of the modules, was exactly the same. We could be sure that the modules and their exchange did not depend on or vary with the choice of thresholds according to the criteria of Supplementary Figure S2; at least the number of nodes preserved was greater than the set point (85% in this study). IC, independent components; DMN, default mode network; VN, visual network.

Volume entropy, a topological invariant of the brain graph using its equivalent of a universal cover, represents the unique characteristics of information flow of the functional graph shape (Figure 1C). It was not found to be linearly related to the total number of edges per time bin. This was scrutinized in this exemplary case. Once the number of edges decreased, either with or without loss of remaining voxels due to thresholding, the volume entropy proportionally decreased to the number of edges per brain graph (Figures 3A,B). When the lower limit of the total number of nodes was set to above or equal to 1,265 (85% of the total 1,489 voxels), the number of graph voxels did not influence volume entropy. In positive graphs with correlation thresholds below or equal to 0.4 (ranging from 0.2 to 0.4), the calculated volume entropy remained constant across the graphs. At the same time, the total number of edges varied from 78 K (average for 280 time bins at a threshold of 0.5) to 330 K (average for a threshold of 0.2). As the threshold increased from 0.45 to 0.85, the total edge number decreased initially, following a curvilinear decrease in volume entropy, and then decreased linearly from the threshold of 0.65. In negative graphs with thresholds above or equal to −0.4 for anticorrelations (or below 0.4 for unsigned anticorrelations), the volume entropy remained constant across the graphs. At the same time, the total edge numbers varied from 45 K (for a threshold of 0.4) to 193 K (for a threshold of 0.2).

Notably, the volume entropy was the same at thresholds lower than certain values in both positive and in negative graphs, as they shared the same skeleton structure of information flow on the brain graphs regardless of the thresholds below these values. Extra surplus edges of the graphs with lower thresholds did not affect volume entropy, the topological invariant that represents information flow (Supplementary Figure S2). In other words, unlike the total number of edge-dependent k-core values, the volume entropy of a graph, whether positive or negative, represents the total amount of information flow capacity of a graph, independent of the additional number of edges therein. This finding was reiterated in the pattern of afferent capacity on the directed graphs, particularly in the positive graphs (Figures 3C,D). The modular shapes of the temporal progression of the afferent capacity of the positive graphs were precisely the same when the threshold was below or equal to 0.7 (i.e., from 0.2 to 0.7), and this was also the case when the threshold was below or equal to 0.55 (from 0.2 to 0.55) in the negative graphs (Supplementary Figure S2). For the afferent capacity, the MRI-overlaid animation maps on the run showed the same pattern of modules and their exchanges, which was also the case for the timepoint plots of the positive graphs, regardless of the thresholds when they were above 0.7.

With these observations and analyses, it was found that the edge number affected the k-core values. Therefore, the k-core values were corrected by dividing them by their total edge number (edge-scaled k-core) per time bin. We compared these edge-scaled k-core values between intertime bins within individuals, between individuals, or between positive and negative graphs. In contrast, volume entropy and afferent/efferent node capacity were unaffected by the surplus edges when the thresholds were low enough, and we did not perform edge-scaling for volume entropy or afferent/efferent capacity.

Optimal thresholds were determined by (1) k-core (and its accompanying k_max_core) value, (2) volume entropy (and afferent/efferent node capacity), and (3) scale-free degree distribution among various thresholds in addition to the number of voxels (85%) (Supplementary Figure S2). Using separate thresholds for positive and negative graphs, we further analyzed the state fluctuations and transition of the k_max_core maps with a glass brain representation to determine the dynamic voxel hierarchy. We also analyzed the capacity of afferent and efferent nodes overlaid on MRI slices in 180 HCP subjects, as well as the afferent/efferent capacities of voxels on their corresponding timepoint plots. With these preset thresholds, we sought the state fluctuation or transition associated with modules and their exchanges of afferent and efferent capacity of voxels or ICs in both positive and negative graphs.

### k-core voxel values represent the hierarchical position of voxels in resting-state brain functional graphs

The intervoxel correlation of the voxel waveform amplitudes exhibited an almost symmetric distribution in the 17 million-edge histograms. We divided the positive and negative edges to construct positive and negative graphs, respectively, which are mutually exclusive and interdependent. Edges with negative correlation (intervoxel anticorrelation) were converted to unsigned values; thus, a negative graph is, in fact, an absolute-valued negative network. All subsequent analyses were performed for positive and unsigned negative networks (Figure 1B).

After thresholding the brain graphs with the appropriate correlation values, the brain graphs were distribution-free (descending mostly linearly on the log–log plot in the voxel degree-prevalence distribution), preserved as many voxels [5,000 (85% of the total)] as possible and had a sparse number of edges (0.5–10%) along all the time bins and individuals from the HCP (*n* = 180). Python codes were used to perform k-core percolation as previously described (Azimi-Tafreshi et al., 2019; Whi et al., 2022b). k-core values were annotated to 5,937 voxels and overlaid on 36 slices of MRI transaxial images (Supplementary Figure S3A). For 280 time bins of 180 individuals from the HCP project, animation movies for k-core voxel value maps revealed all *k* values, which varied in total sum and thus waxed and waned on animated displays. The total sum of the k-core values per graph was equal to the total number of edges for each time bin (Supplementary Figures S1G,H), while highly ranked k-core-valued voxels were displayed in scatters or clusters. According to the theoretical reasoning and the practical observation of the equivalence of the total number of edges and the total k-core values per time bin, animation movies were finally made using the edge-scaled k-core index (k-core voxel values divided by the total edge number per time bin), as well as animated edge-scaled flag plots and edge-scaled k-core timepoint plots (Supplementary Figures S3C,D).

The qualitative readout of these image-animation sets reveals fluctuating changes in hierarchical intensity, where red represents the highest position on the hierarchy, and blue represents the lowest position. Additionally, the jumping effect changed from one cluster to another cluster of hierarchically prominent voxels. The hierarchical dominance of clustered voxels seemed to be sustained for a varying short period and then quickly replaced by other clustered voxels. A stacked histogram for the hierarchically highest voxels on k_max_core plots showed an abrupt and sharp transition of the dominant voxel clusters. We referred to this abrupt transition of k_max_core voxels as the “state transition” of the hierarchically highest voxels (Supplementary Figure S3B). On the animated glass brain images of lateral (sagittal) and dorsal (transaxial) views, we explored the alternating participation of voxels belonging to the same ICs (Supplementary Figure S3C). Seven well-characterized ICs and their voxels are colored using rainbow colors. Animated k_max_core voxels/IC composition images, either stacked histograms or glass brains, easily revealed the hierarchical dominance of, for example, visual network (VN)-dominant k_max_core voxels or default mode network/central executive network (DMN/CEN)-dominant k_max_core voxels and their transition from the VN to the DMN/CEN from the DMN/CEN to the VN (Supplementary Figure S3D). Other combinations of k_max_core voxel/IC compositions could also be observed with every possible transition. In our previous report (Huh et al., 2022), we measured the intraoperator reproducibility of counting the number of state transitions, and the resulting reproducibility was indicated by a Pearson’s correlation coefficient of 0.88 (Supplementary Figure S4). These surveys were supplemented by animated flag plots of voxel k-core/IC compositions, which are the shuffled voxels displayed in the animation (Supplementary Figure S3D). This state transition was easily observed in the positive networks of almost all 180 normal adults (third to fourth decade in age) but was rare in the negative networks.

**Figure 4:**
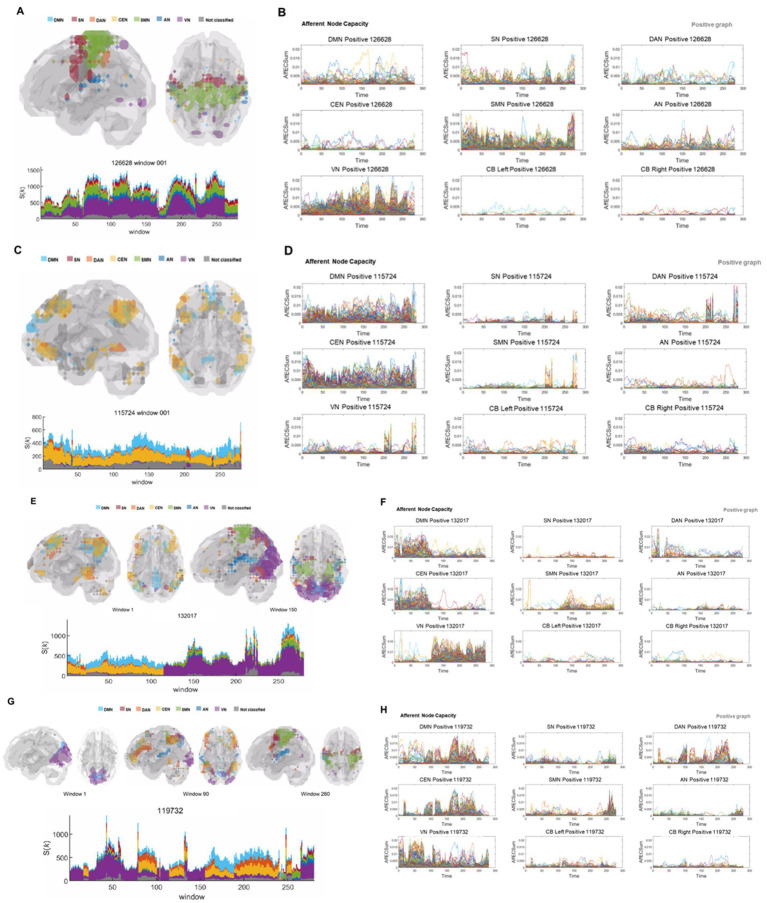
Various types of states and state transitions on k_max_core voxels/ICs from the DMN to the VN and modules, and their exchanges on afferent capacity voxel/IC timepoint plots. The color strips range from sky blue (DMN), red (SN), orange (DAN), yellow (CEN), green (SMN), blue (AN), violet (VN), and gray (unclassified). **(A)** Glass brain image animation and stacked histogram plots along 280 time bins of the k_max_core. Voxels belonging to VN, AN, SMN, SN, and DMN joined the k_max_core all around the time bins. No state transition was observed in this individual, and the total number of k_max_core voxels fluctuated. See Supplementary Video S1. **(B)** Afferent node–voxel capacity, labeled AffECSum, was plotted along the time bins separately for ICs annotated for every voxel, such as the DMN, SN, DAN, CEN, SMN, AN, VN, L Cbl, and R Cbl. The density of modules composed of the VN, SMN, SN, AN, etc. varied over time, but there was no dropout or exchange. A few loose threads were observed in the voxels/IC plots of the DMN, CEN, DAN, and AN. **(C)** Glass brain images and stacked histogram along 280 time bins of the k_max_core. Voxels belonging to DMN, CEN, and the unclassified group were the dominant and exclusive participants of the k_max_core throughout the time bins. Few state transitions were observed in this individual at approximately the 205th and 245th timepoints. See Supplementary Video S2. **(D)** On time-varying afferent node capacity voxel plots derived from positive brain graphs, voxels belonging to the DMN, CEN, and unclassified network dominated with larger afferent node capacities continuously during the entire period. The VN, SMN, SN, and AN splashed briefly at the end of the period. **(E)** In this individual, k_max_core plots revealed initial DMN and CEN dominance and later VN dominance with a small SMN or DMN or others. See Supplementary Video S3. **(F)** Afferent node capacity voxel plots corroborated the k_max_core plots, implying that k_max_cores were run by positive afferent node–voxel capacity, the sum of node-linked edge capacities coming in from other voxels, of the DMN and CEN, with a small amount of DAN during the first half of the period. At approximately the 115th timepoint, the dominance of the DMN and CEN voxels abruptly abated, and the dominance of the VN voxels decreased with little help from the SMN and scant help from the DMN, DAN, and CEN. **(G)** Glass brain images and stacked histogram of the k_max_core. State transitions from VN dominance to DMN, CEN, and DAN, or vice versa, are repeatedly shown. At the 80th timepoint, a sharp transition from sole VN dominance to DMN, DAN, CEN, and VN dominance was found. A single-petal bin preceded just before the transition. Between the 100th and 150th time bins, VN dominance occurred first, followed by DMN, DAN, CEN, and unclassified codominance. See Supplementary Video S4. **(H)** Afferent node–voxel capacity, labeled as AffECSum on the ordinate, plots. Previously developed ICA using images of 180 static individuals defined the modules of ICs. Interestingly, the DMN, DAN, and CEN modules gathered together to make module congregates of similar (but with small variations) progress over time bins. The modules were prominent for all ICs, the left and right cerebellums, and the unclassified. Attention might be given to time bins around the 100th one, which showed a razor-sharp transition from the DMN, DAN, and CEN comodules to the VN module and back to the DMN comodules. This was a clear representation of the effect of the k_max_core ‘state transition’ on afferent node capacity. IC, independent components; DMN, default mode network; SN, salience network; DAN, dorsal attention network; CEN, central executive network; SMN, sensorimotor network; AN, auditory network; VN, visual network; Cbl, cerebellum; L, left; R, right.

**Figure 5:**
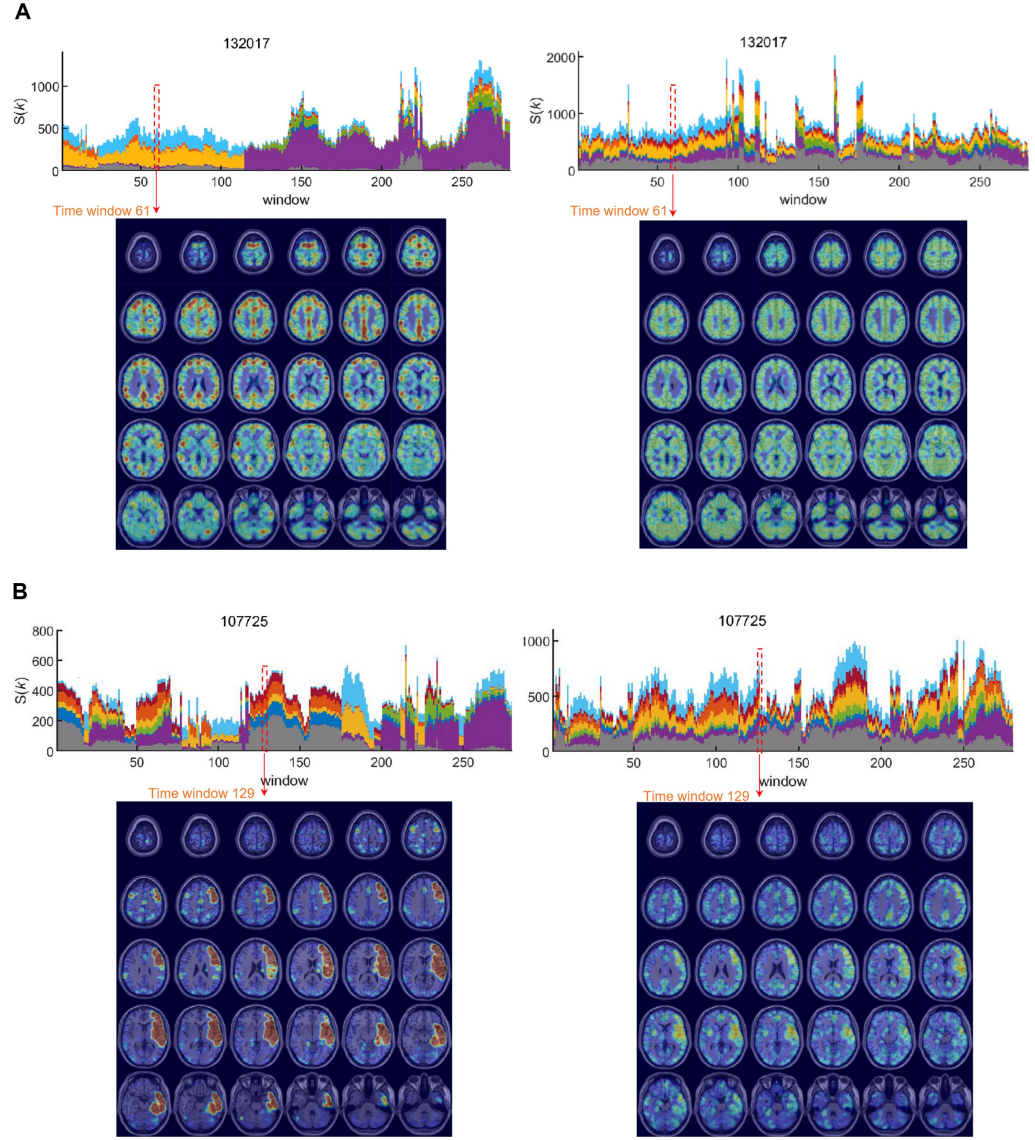
Hierarchically top-tier voxels on stacked histogram timepoint plots of k_max_core voxels or hierarchically colored MRI-overlaid animation plots. Animation images were captured via snapshots for visualization. **(A)** In this individual presented in the above Figure 4E, who showed initial DMN and CEN dominance and later VN dominance in positive graph (left side of this picture), in the unsigned negative graph on the right side, a sustained state of voxels belonging to most ICs was observed across all the time bins. This case was representative of the negative graphs of all individuals, in whom the edge number scaled (abbreviated edge-scaled) k-core images showed a characterless flickering pattern of ripples. See Supplementary Videos S5A,B. **(B)** In this individual, the positive graph presented a typical state transition on the stacked histogram timepoint plots of k_max_core voxels but extraordinarily showed the top tier of the left frontal cortex (Broca’s area) in the first half of the time bins’ progression, intervened by right frontal lobe leveling up (arrows) on the k-core animation plots. However, the negative graph did not show any abrupt state transition but only fluctuations in the stacked histogram of k_max_core. At later timepoints (125th to 170th), the left frontal prominence of the negative graph accompanied the prominent left frontal area of the positive graph (120th to 180th). See Supplementary Videos S6A,B. IC, independent components; DMN, default mode network; CEN, central executive network; VN, visual network.

Among 180 individuals, the number of state transitions ranged from none to the most frequent in the positive graphs (Figure 4 and Supplementary Videos S1–S4). State transition was not observed in 17 subjects, as the same state persisted throughout the entire period (Figures 4A,C and Supplementary Videos S1, S2). Only one state transition was observed with a half-and-half division of states in 10 subjects (Figures 4E, 5A and Supplementary Video S3), and prominent, typical state transitions were observed on several occasions in 105 subjects (Figure 4G and Supplementary Video S4). The others exhibited an intermediate pattern; i.e., an intermediate pattern between no transition to one with half-and-half transitions (Supplementary Figures S5A,C), an intermediate pattern between one transition to indistinct/a few transitions, and an intermediate pattern between typical (Supplementary Figures S6A,C) or too-frequent transitions (Supplementary Figure S7A). Synchronized combinations of state fluctuations were rare but were present in 12 individuals, as illustrated in the eloquent case, are shown in the Supplementary Figure S8A Asymmetry of module composition of state was easily recognized on edge-scaled k-core animation images, one of which showed alternating contributions of the CEN (from left to right, then to left, and then to right) (Supplementary Figure S9A), another of which showed a left frontal lobe reminiscent of Broca’s area (Supplementary Figure S9C). The other showed ripples in the left cerebellum but not in the right cerebellum (Supplementary Figure S9F). All of these are shown in the positive graphs. Unlike in positive graphs, in unsigned negative graphs, the state transition was not remarkable but was vague, if any, and the k_max_core and k-core animation revealed ripples with infrequent unison-like synchronization (Figure 5 and Supplementary Videos S5, S6). In an exotic case (only one of the 180 individuals, Supplementary Figure S9C), animated k-core plots revealed the explicit state of Broca’s area twice in negative graphs (Figure 5B and Supplementary Video S6), which was the same combination of the left frontal area of the salience network (SN), dorsal attention network (DAN), CEN, and auditory network (AN) in the positive graph of the preceding time bins of the first half of the image acquisition (Supplementary Figure S9C). Otherwise, in all the other individuals, the negative networks did not exhibit a characteristic state composition or pattern of k_max_core. They were ignorant of the individuation of the individuals on their behalf.

As k-core percolation was performed independently for each time bin in the positive or negative graphs produced with the same thresholds for each individual, the total sum of edges varied significantly per time bin within an individual as well as between individuals. In all individuals, the sum of the k-core values across the 5,937 voxels was one-to-one match with the sum of all edges per graph time bin (Supplementary Figures S1G,H). This relationship was without exception. Thus, k-core timepoint plots were made using edge-scaled k-core values and were presented as quantitative plots.

Quantitative edge-scaled voxel k-core values were displayed based on voxels/IC composition, which enabled us to follow the trajectories of each voxel’s k-core values on the timepoint trajectory plots of voxels per IC. From the DMN to the VN, the seven components, as well as the left and right cerebellum and vermis and their corresponding voxels, were displayed using MATLAB or Microsoft Excel. On both outputs, the 10 ICs exhibited quasi-similar contours due to the confounding effect of edge numbers if we used edge-non-scaled voxel k-core for timepoint plots. After edge-scaling, transient increases, decreases, or ripples remained on the contour surface (Supplementary Figures S1A,B). The subtle differences in edge-scaled voxel k-core timepoint plots did not affect the evaluation of the distribution heterogeneity of voxels and their trajectories. In this study, although we used voxel annotation by ICs derived from group static ICA of 180 individuals’ cohorts, voxel trajectories were grouped into recognizable modules for each voxel/IC composition. In addition to the collective behavior of the temporal progression of the voxel/IC composition, these timepoint plots enabled us to follow through the voxel behavior of joining by taking turns in the k_max_core. Each voxel has its own characteristic recognizable trajectory or temporal path along the time bins, meaning that (1) heterogeneity was present between voxels taking hierarchical top-tier positions although their associated ICs were the same and (2) despite this heterogeneity, the voxels for each IC collectively constituted a discernible trajectory along the temporal axis according to the ICs to which they belonged. This was the requisite for the hierarchical temporal progression of voxels themselves and their assigned identity, based on their characteristic belonging to ICs.

### Volume entropy, regardless of surplus edges, functions as a global parameter of information flow over resting-state brain functional graphs

To reduce the computational burden, brain graphs (with a threshold of 0.65 for positive graphs or −0.5 for negative graphs) were downsampled to 1,489 voxels and subjected to volume entropy calculations using the model described in our previous report (Lee et al., 2019). In short, each graph was transformed into a universal cover consisting of nodes and edges from the individual original graph (Figure 1C). According to the theorem of minimum volume entropy (Lim, 2008), a random walker’s journey on the metric surface of the metric ball of the universal cover converges to an asymptotic end as the radius of this metric ball increases to infinity. The topological invariant, volume entropy of a brain graph, h, on the exponential term is now defined on the metric ball of the universal cover of the original graph (Figure 1C). In the previous study (Lee et al., 2019) and in the following application study (Ha et al., 2020), instead of using numerical analysis to find asymptotically converging values of volume entropy, we applied a generalized Markov system on the edge transition matrix and eigen decomposition. Volume entropy is a global measure that represents the total sum of the information flow over the edges of functional brain graphs, whether positive or negative.

The volume entropy of a graph depends on the number of nodes and edges, and the graph’s constitution. We first tried to remove the confounding effects of the number of nodes and then to understand those of the edges. In the positive and negative graphs, we used graphs with the same thresholds of our choice for all the HCP individuals. The number of nodes was higher than 70% of 1,489 voxels (>85% of 5,937 voxels). We found that the number of nodes did not affect the volume entropy on the timepoint plots when we compared the volume entropy of time bins per se and the volume entropy divided by the number of nodes (Supplementary Figure S10A). Therefore, although variable, if above a certain percentage of nodes were included, we could ignore the effect of the number of nodes. The impact of the number of edges was slightly more complex, as expected, regarding the relationship between the number of edges and the graph structure.

By varying the thresholds in individual graphs and observing the entire cohort, we obtained the following findings. First, the number of edges per time bin varied greatly (from 5,000 to 200,000) for every individual. Volume entropy decreased proportionally to the number of edges below a certain threshold, specific to each brain graph. For example, first, in an individual (Figure 3A), an average of 50,000 or fewer edges showed a coarsely proportional decrease in volume entropy. Second, in this individual, the average of approximately. 330 K (threshold of 0.2) to 78 K (threshold of 0.5) edges in positive graphs or approximately 193 K (threshold of 0.2) to 45 K (threshold of 0.4) in negative graphs, the volume entropy was approximately 9–12 million in positive graphs, or approximately 8.1–9 million in negative graphs (Figures 3A,B). Third, unlike nodes (regularized within an individual, referred to as edge-scaled), the volume entropy divided by the total number of edges waxed and waned in timepoint plots in all individuals (Supplementary Figure S10A). Volume entropy with preset thresholds for an individual (across all time bins) or for the entire cohort of individuals could not be used for comparisons (Supplementary Figure S11). Nor could the edge-scaled volume entropy per time bin. This observation limited the use of volume entropy in brain graphs as a global parameter of information flow capacity for comparisons among individuals; instead, the co-production of afferent and efferent node capacity on directed brain graphs could be used. The timepoint plots of the afferent capacity of the positive graphs revealed module formation and exchange. The same modules with voxel/IC compositions and their changes were identified (Figures 3C,D). The plateauing relationship between volume entropy and the total number of edges with lower thresholds (e.g., ≤0.4 in positive graphs) is the first observation that the surplus edges did not contribute to the globally cumulative information flow in a graph represented by volume entropy, a topological invariant of a graph (Figures 3A,B).

### Directed functional brain graphs yielded afferent/efferent voxel–node capacities as surrogates for edge transition matrices

Edge weights of nodes to and from any nodes yielded edge transition matrices, which were asymmetric in our previous study by Lee et al. (2019), using 274 node regions. By incorporating pairwise intervoxel correlation in this study, rather than the previous inter-regions of interest correlation, we reproduced the asymmetry of directed edge matrices per time bin. The edge matrix was huge, with a rare possibility of direct visualization; thus, the edge metric was converted to a node metric. That is, from the edge matrices, marginal values were calculated to yield afferent (sum of columns representing “to the node”) and efferent (sum of rows representing “from the node”) values. These afferent and efferent node capacities were overlaid on the MRI slices, and the time bins were merged to create audio–video interleave (AVI) files for animation playback. Positive and negative graphs, along with their afferent and efferent node capacities, produced 2 × 2 (a total of four) animations per individual (Figure 1 and Supplementary Video S7).

**Figure 6:**
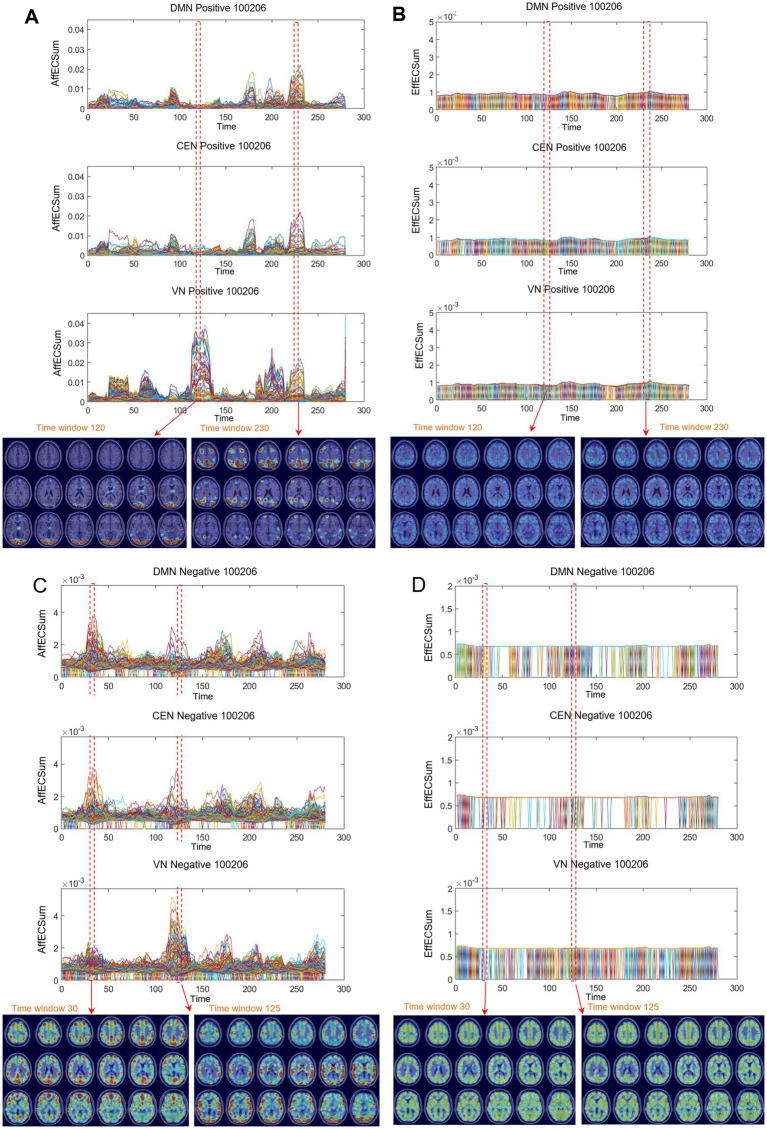
Differences between positive and negative graphs of afferent and efferent voxel capacity timepoint plots were common on the directed graphs of all the individuals. Afferent node capacity showed characteristic patterns on both timepoint plots and MRI-overlaid map animations. Afferent capacity was greater than efferent capacity for voxels in general and for voxels/ICs. Afferent capacity of the positive graph was greater than that of the negative graph. **(A)** Time-bin timepoint plot of voxels/DMN and voxels/VN of afferent node capacity of positive graphs. Module formation and switching were visualized with MRI-overlaid animation plots (shown here with snapshots of the 120th to 230th time bins) for afferent node capacity. The maximum height of the module was 0.05, which was 50 times greater than the 0.001 for the efferent node capacity. See Supplementary Video S7A. **(B)** Timepoint plots of efferent node capacity were homogeneous, which was also well observed in the animation plots with monotonous snapshots. This characterlessness was common in all the individuals. See Supplementary Video S7B. **(C)** Timepoint plot for the afferent node capacity of unsigned negative graphs. The contour and trajectory of the voxels/IC (DMN, AN, and VN) looked similar to those of the positive graphs. However, the height of the module was only one-seventh that of the positive graphs. Nevertheless, the 0.006 afferent node capacity in the negative graph was almost 9 times the efferent node capacity (0.0007) in the negative graph. See Supplementary Video S7C. **(D)** Monotonous and smaller efferent node capacities of negative graphs are shown. See Supplementary Video S7D. DMN, default mode network; CEN, central executive network; AN, auditory network; VN, visual network.

The animated afferent node capacities of positive graphs were unique in their revelation of voxels’ composition of modules and their exchanges along the time-bin axis, but not efferent capacities or afferent/efferent capacities of negative graphs (Figure 6). As each time bin’s edge capacity and volume entropy calculation were already normalized during calculation (Lee et al., 2019), comparable modules popped up from the baselines with exponential height, took temporary union in various combinations of ICs, and took turns, which we called ‘module exchange’ (Figure 6A and Supplementary Video S7A). For example, in these positive graphs, DMN voxels frequently coalition with CEN voxels, SN voxels with AN or sensorimotor network (SMN) voxels, or VN voxels with a variety of IC voxels. The transition from prominent DMN/CEN modules to VN modules, or vice versa, was observed frequently during our read-outs. On the animated afferent node capacity of positive graphs, we observed a waving or undulating progress with collective ICs, sometimes almost all the ICs (Supplementary Figure S12A). In contrast, animated efferent node capacities of positive graphs were nearly stationary with small multifocal flickering on MRI-overlaid brain animation plots (Figure 6B and Supplementary Video S7B). Animated afferent node capacities of negative graphs showed ripples of collective voxels (Figure 6C and Supplementary Video S7C). Negative graphs tended to rarely generate modules, which differed from the afferent node capacity of positive graphs but also formed the unison of smaller module collections with much lower maximum heights of modules (one-third to one-eighth of positive graphs) (Supplementary Figures S12, S13). The animated efferent node capacities of the negative graphs were stationary with multiple small flickers (Figure 6D and Supplementary Video S7D).

The quantitatively assessed afferent node capacities were displayed in voxel-run timepoint plots (point line-connection figures) based on the voxels/IC composition, which followed each voxel’s spatiotemporal trajectory of afferent node capacity along the time bins for each IC (Figures 6A,C; Supplementary Videos S7A,C; Supplementary Figure S12A). Afferent node capacities of the positive graphs exhibited individually distinct envelopes of the voxels’ trajectories for each IC, characterized by exponential increases and decreases, as well as on-and-off movement along the temporal time-bin axis. These modules were interchangeable between any combination of ICs, changing from one IC to another and vice versa. This module exchange reminded patterns of state transition of animated stacked histograms of k_max_core voxels.

**Figure 7:**
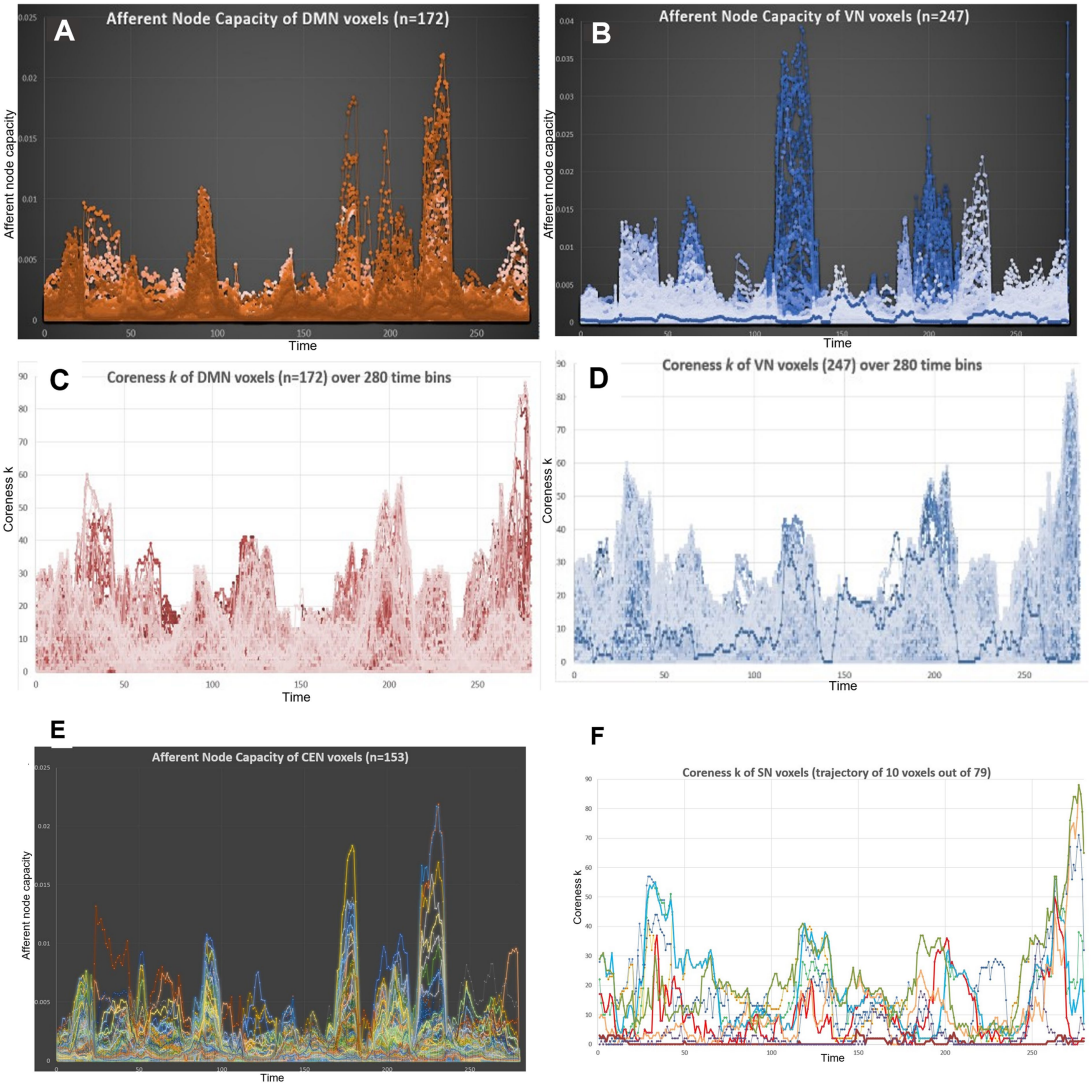
The voxel trajectories showed extreme heterogeneity in terms of the afferent node capacity and edge-scaled k-core timepoint plots. **(A)** A total of 172 voxels of the DMN were traced along the 280 time bins. The plot revealed that each voxel took its own trajectory, meaning that it contributed once to form a module and then at the next module, either idled or contributed, and then at the third module, unexpectedly behaved, etc. Despite these behaviors of individual voxels, an IC in this case, the DMN made a plausible module to be named a characteristic IC with the support of the voxels’ unpredictable trajectories. **(B)** The behavior of the 247 trajectories of VN voxels was similar to that of the DMN voxels. VN voxels seemed to consist of two clusters to make modules, that is, if we labeled 1–5 for medium-to-large-sized modules from the start, whitish modules of 1 and 5, bluish modules 3 and 4, and mixture, the two clusters might have been dissociated as separate ICs beforehand. **(C)** and **(D)** k-Core (edge-non-scaled) showed the same contour for the DMN and VN. This was because the total number of edges per time bin was the major determinant of total k-core. The behavior of the trajectories of each voxel, either the DMN or VN, followed its own fate to take any or no route of contribution precariously to form ICs, but collectively made the rise and fall of each peak. **(E)** A total of 153 CEN voxels were followed for their trajectories of afferent node capacity. One can easily follow the luxury of heterogeneity of each voxel. **(F)** Trajectory images with entire voxels/IC would have made resolving impossible; thus, we chose 10 random trajectories of the SN (79 voxels). Timepoint plots of these 10 voxels showed the heterogeneity of trajectories, making it unbelievable that they belonged to the same IC or SN. IC, independent components; DMN, default mode network; VN, visual network; CEN, central executive network; SN, salience network.

Interestingly, voxel trajectories were highly heterogeneous in making modules for every IC. In other words, when we used an Excel chart display for every voxel (*n* = 69–247) belonging to ICs (AN ~ VN) with the capability of each voxel trajectory tracing, a voxel was on the highest swing in one module but on the baseline without any ascent in the next module (Figure 7). *A priori* entitlement of voxels could only manifest the correspondence of voxels to a specific IC. This pattern was universal among individuals in the cohort in terms of the voxels’ behavior along the temporal axis, as it belonged to an IC macroscopically and groupwise; however, participation was *ad hoc* and alternated with that of the colleague voxels (Figures 7E,F). This immediately defied the simplicity of the region of interest (ROI) approach for following up the collective trajectories of voxels in an ROI. The center-of-mass assumption of inter-ROI correlation was computationally convenient but based on an incorrect assumption in the previous ROI, as investigated in studies including ours (Lee et al., 2019; Ha et al., 2020).

### Voxel hierarchy and afferent capacity of functional brain positive graphs represent resting-state transition at rest

We asked questions regarding whether the voxel–node hierarchy of a dynamic functional brain graph can be represented by the afferent or efferent node capacity thereof for each individual, either or both on the positive and the unsigned negative graphs. While voxel k-core values were derived from the undirected graphs, which were the aggregates of in- and out-degrees of voxel nodes, afferent and efferent node capacity was derived from the directed graphs produced independently using universal cover/Markov modeling, which separately represented the afferent (incoming) and the efferent (outgoing) edges with their own weights (Figure 1). Both the voxel k-core value and afferent node capacity timepoint plots, as well as their overlaid MRI animations, revealed the module exchanges during the progression of the temporal time bins, but not on the efferent node capacity plots. In the positive graphs, the animated stacked histogram (or glass brain display) of the k_max_core voxels (Figures 4A,C,E,G; Supplementary Videos S1–S4; Supplementary Figures S5A,C, S6A,C, S7A, S8A, S9A,C,E) and the animated afferent node capacity of the voxels/IC composition (Figure 6A; Supplementary Videos S7A,C; Supplementary Figure S12A) well disclosed these module exchanges, corroborating each other. Unique to the afferent node capacity of the positive graphs, the timepoint plots of the afferent node capacity well represented the voxel/IC module formation and exchanges and are presented in the Figures 4B,D,F,H and Supplementary Figures S5B,D, S6B,D, S7B, S8B, S9B,D,F. However, this was not the case for the efferent node capacity, even in the positive graphs (Figure 6B; Supplementary Video S7B; Supplementary Figure S12B). In unsigned negative graphs, rare examples of module exchange were present (Supplementary Figures S13A,C,E,G), even in the afferent capacity, but obviously not in the efferent capacity. Thus, positive graphs, but not unsigned negative graphs, were the main source of fMRI evidence of module exchanges. Notably, the voxel k-core was derived from undirected graphs, and the afferent and efferent voxel capacities were derived from directed graphs.

The next question was whether positive animated stacked histograms of k_max_core and/or positive animated afferent node capacity are complementary or redundant for revealing state transitions by showing the exchange of voxels/IC composition within modules. Both methods were found to reveal similar numbers and timing of state transitions, which were sometimes clearer in positive animated stacked histograms of k_max_core voxels and, at other times, more easily recognized in positive animated afferent node capacity brain overlaid images. Scrutiny of the timepoint plots of both the k_max_core and afferent node capacity revealed that they were the same in their ability to identify state transitions or module exchanges during the resting state. However, these observations in individuals did not overlap entirely, and one did not include the other. Eventually, trajectory tracing of single voxels, for example, enabled us to determine that their trajectories were unrelated to each other (Supplementary Figure S14). Voxel/IC timepoint plots of afferent node capacity and stacked histograms of k_max_core voxels were the most effective for revealing the state transitions and module exchanges once they were observed in collective plots along their IC compositions. Both parameters were helpful in revealing the time bins of the start and end of the modules, the combination of the modules, and the contribution of combination products (simultaneously activating voxels belonging to the ICs at that moment), not exclusive to one or the other modality.

Timepoint plots of k-core voxels *per se* were found to be intensely dependent on the total number of edges for each time bin; thus, the seemingly self-similar feature *per se* was an artifact of the confounding effect of varying the total number of edges. In contrast, the k_max_core was free from this confounding effect, at least within certain threshold ranges. The afferent node capacity on the directed positive graphs was theoretically and data-wise free from the edge-number confounding effect. The following enabled the discovery of resting-state fMRI evidence of fluctuating/transitioning human resting states: confirmation of the scale-free degree distribution, finding the hierarchical skeleton of brain graphs and the subsequent k-core percolation, relabeling the centrality of voxel degrees with hierarchically structured values of k-core and, finally, an information/graph topological approach producing directed graphs and afferent/efferent node capacity. Both methods independently went on to unravel module switches, reminiscent of resting-state transitions in their own ways, and corroborated each other. Eventually, the timepoint plots of both measures clearly showed that they were not adequately proportional to cancel or include one within the other (Supplementary Figure S14).

The following simple question was whether the voxels’ behavior, along the temporal axis of the time bins, occurred on the two-dimensional (2D) plane of the abscissa of the hierarchy, that is, the k-core value and the ordinate of the afferent node capacity. From the positive graphs, an exemplary case was selected, and an IC was selected. When the trajectory of a voxel (and others) was drawn along the temporal progression on this plane, exuberant paths were produced, which need to be modeled in future studies (Figures 7E,F). The heterogeneity of these voxel trajectories, both in terms of hierarchy and of afferent node capacity, was another advantage of these methods, which involved animated display of pairwise voxel-based amplitude correlation studies in resting-state fMRI.

**Figure 8:**
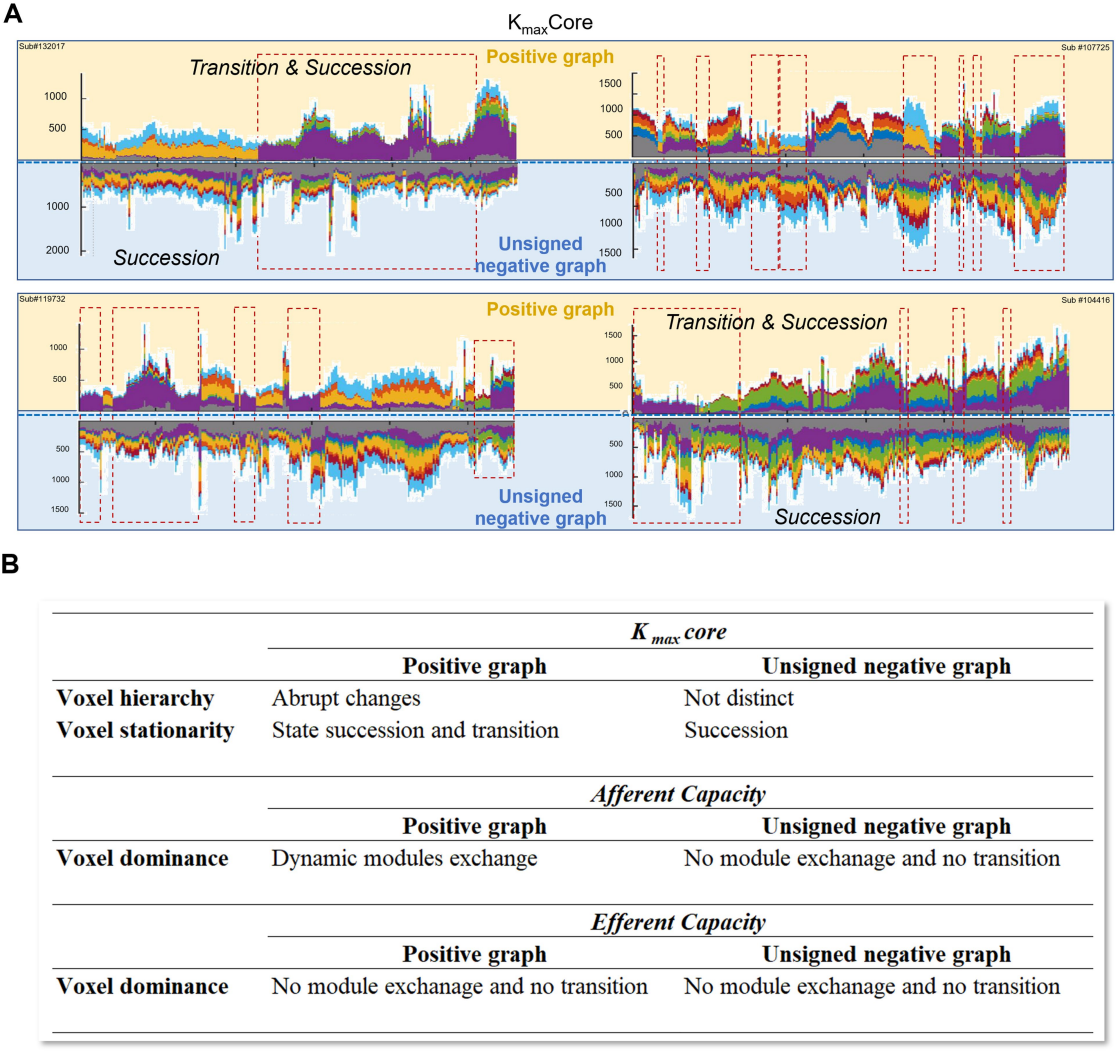
Graphical summary and tabulation of the results. **(A)** The spatiotemporal progression of hierarchical structure was investigated in dynamic positive and unsigned negative brain graphs, respectively. Spatiotemporal progression of voxels’ hierarchical information was visualized in four representative subjects. Voxel hierarchy of positive graphs showed transition and succession; however, the transition was not distinct in the unsigned negative graph. The negative networks did not show a characteristic state composition or pattern of k_max_core. **(B)** The voxel hierarchy in positive graphs displayed abrupt changes and state transition/succession through animated coreness maps and k_max_core representation, such as stacked histograms and glass brain visualization. The afferent voxel capacity in the directed graph showed dynamic module exchange in the positive graph, but not efferent capacities in the positive graph or afferent and efferent capacities in the negative graph.

To compare the state progression and transition patterns of k_max_core over time, we also analyzed four centrality measures from graph theory, such as degree centrality, eigenvector centrality, betweenness centrality, and clustering coefficient. As shown in the Supplementary Figure S15, except for betweenness and clustering coefficient, the other measures exhibited considerable similarity, as seen in the glass brain visualization. However, in the stacked histogram, we observed that k_max_core changes were more pronounced in revealing transitions compared to degree, eigenvector, and strength centrality. To compare with k_max_core, we identified the maximum voxel for each measure by determining the percentage of voxels corresponding to k_max_core in each time window and designating voxels with values equal to or above this threshold as max voxels for the respective measures. To facilitate the understanding of complex results, Figure 8 presents a tabulation of summarized results and a reconstructed version of Figure 5.

## Discussion

In this study, state progression and transition on resting-state networks were investigated using two separate methods: k-core percolation on the degree sequence and information flow analysis of constructed directed graphs, both positive and negative. k-Core percolation overcame the current ignorance of voxels, producing collective trajectories that give rise to emergent modules and state transitions at rest. Disregarding the principal components, we focused on the voxels on the hierarchical ladders of functional brain graphs, exploring their transience of combinatorial actions. Information flow measures of topological invariant searches of volume entropy allowed us to construct the most probable directed weighted graphs and their corollary quantitative afferent and efferent capacities. This latter approach automatically transformed pairwise edge information into node characteristics, known as afferent or efferent voxel–node capacities. Fortunately, both methods demonstrated the presence of state and state transitions at rest caught by resting-state fMRI and visualized the modules composed of voxels pre-annotated to ICs with either animation or timepoint plots. Voxel trajectories have now been understood to join popular ICs, that is, the DMN, CEN, SN, VN, or others. Further investigation of trajectory clustering is warranted to understand their within-IC taking turns in timepoint progress. Of course, the underlying mechanism of state transition or module switching can be elucidated by combining phase coherence and amplitude correlation. The role of negative correlations (i.e., anticorrelations) requires further study, although we considered negative correlations as unsigned correlations in this study and found that their unsigned pseudo measures did not contribute substantially to the state transition. We have briefly summarized our results as a graphical figure and tabulation in Figure 8.

Unlike structural brain graph interpretation, functional dynamic brain graph interpretation requires understanding graph flexibility. When we interpret functional brain graphs, we need to note that functionally, the brain is not a closed system but an open system receiving inherent emergent (Bianconi and Rahmede, 2017; Calmon et al., 2022; de Schotten and Forkel, 2022; Calmon et al., 2023) and/or external perturbation stimuli (Brändas, 2024). This is also the case for the resting state, although people erroneously assume that the stationary state of the human mind will be observed in resting-state fMRI studies. Neuron–glia complexes consisting of brain voxels serve as connected or independent identifiers of inherent emergent behaviors, with the characteristics of a microcanonical ensemble (Wu et al., 2013; Huh et al., 2022; Whi et al., 2022b). This openness of voxels leads to the spatiotemporal expansion of interactions with adjacent and remote voxels, resulting in graph structures that exhibit both positive and negative correlations/coherences. The sliding-window analysis method for brain blood oxygen level-dependent (BOLD) waveforms needed to be chosen to discretize the temporal expansion of the fMRI signals, which made the analysis computationally burdensome. Fortunately, computing resources, whether hardware or software, are available and affordable; thus, voxels and their trajectories can now be traced. The observables from this voxel-wise volume of the brain graph consist of voxel-sized quanta spatially and 1-min window-sized quanta temporally. Functional interactions between adjacent or remote voxels are endowed with a luxurious time for 1 min (several rounds of global communication via electrical and chemical interactions between neuron–glia complexes within each voxel), and adjacency or neighborliness in their similarity characteristics of functional correlations are then free from any constraint of structural connections between voxels. The BOLD waveforms of voxels and the intervoxel correlation values thereof now depend solely on their observed correlation and coherence. In other words, the behavior of several thousand voxels is more likely to be that of a three-dimensional lattice, where the nodes can communicate within the observed period of 1 min, regardless of the Euclidean physical or structural distance. Only the observed similarity between voxels dictates further interpretation of brain graphs. We did not integrate phase coherence in this study.

Percolation has been used for decades to understand the configuration space or the temporal axis, and phase transitions are well understood in statistical physics. Pioneering works have reported the use of percolation in brain mapping and connectivity analysis (Alexander-Bloch et al., 2010; Gallos et al., 2012; Morone and Makse, 2015; Del Ferraro et al., 2018). This percolation was also adopted to understand hierarchical organization and optimal thresholding of functional brain graphs (Bardella et al., 2016; Nicolini and Bifone, 2016; Whi et al., 2022b). Degree was commonly used to find the hierarchy of nodes as well as edges using k-core percolation on resting-state fMRI. Most recently, investigators used transfer entropy (TE) using a multivariate TE toolbox of MATLAB for electrophysiological measurements in primates to understand motor behavior-related hierarchical organization of directed weighted graphs derived from TE preprocessing. TE is another excellent measure of representing directed information flow over the edges made of a hundred nodes (Bardella et al., 2024). Compared to this approach, we used an adjacency matrix derived from an amplitude correlation matrix, applied k-core percolation to spatiotemporal data, and considered undirected connections between 18 million voxel pairs. Temporal sampling and its tracing of top-tier voxels were the uniqueness of our study. Directed information flow over the spatiotemporal functional brain graphs was investigated using another directed graph format, whose afferent and efferent capacities overlaid on the voxel nodes to reveal the state transition, which was observed only in the afferent capacity. Information flow was observed without neuronal measurement (instead, using BOLD) or percolation process (instead, using afferent capacity overlaid on MRI templates).

In the Introduction section, the first assumption was announced to be untestable in our investigation (Hutchison et al., 2013; Mooneyham et al., 2017; Gonzalez-Castillo et al., 2021); however, on resting-state fMRI, we discovered modular on-and-off phenomena and module exchanges at rest in humans. Edge weights and simplified adjacency matrices, which represent degrees, have been used for understanding networks. The long-held assumption that every edge could be treated as equal was challenged by investigators using percolation (Bordier et al., 2017; Huh et al., 2022; Whi et al., 2022b; Bardella et al., 2024). We also challenged another assumption that undirected graphs would suffice to understand the mechanism of functional brain graphs safely, using the observed amplitudes and their correlations on fMRI. Correlation analysis produces only undirected graphs, and investigators used transfer entropy (Bardella et al., 2024) to make directed graphs. We tested the following questions: (1) What if we eliminate the equality of edges and adopt hierarchical repositioning of adjacency matrices (and degrees) into k-core and (2) What if we create directed graphs using a graph universal cover/Markov model and observe the afferent voxel–node capacity of these directed graphs. This approach overcame the bottlenecks of limited representation and consequent insufficient understanding of the dynamic states on resting-state fMRI, which were now shown to be non-stationary. *Post hoc*, we suggest that the state transitions or module exchanges on fMRI represent spontaneous resting-state succession or transition at rest in humans.

The second assumption was whether sliding-window time binning of resting-state fMRI (Figure 1) reveals the waxing and waning behavior of modules or states made by the gathering and/or dissociating of voxels to demonstrate the continuous changes in the composition of voxels/ICs on timepoint plots (Hindriks et al., 2016; Mokhtari et al., 2019; Lurie et al., 2020; Whi et al., 2022b). In the unsigned negative graphs, spontaneous succession without any abrupt transition of states was noted in the temporal progression of the animated k-core/k_max_core images of all the individuals. In contrast, in the positive graphs of almost all individuals, in addition to the fluctuating voxels/IC composition, abrupt state transitions were also observed. The duration of one state varied, and the number of state transitions from one to another varied likewise among individuals [15 min in the HCP database (Van Essen et al., 2013; Elam et al., 2021)] and within a session [5 min in the standard positron emission tomography (PET)/fMRI or Kirby database (Choe et al., 2015)], as detailed in our preprint in bioRxiv (Huh et al., 2022). Figures 4–6 and Supplementary Figures S5–S9, S12, S13 show the k_max_core and afferent capacity spectra of voxel nodes in representative individuals. A 15-min period of resting-state fMRI was sufficient to reveal the diversity of human resting states and their succession/transitions. We suspect that we observe the luxury of fluctuating states in individuals over the 15-min period. If we had only observed the first or last 5 min, we might have seen partial results and related our findings to the characteristics of the subjects, which might have led to categorizing their traits erroneously (Whi et al., 2022b). In our previous report (Huh et al., 2022), a person in the Kirby project underwent a resting fMRI study once a week for 3.5 years, which was visualized using our k_max_core voxel/IC composition timepoint plots. The dominant compositions were mostly similar during certain consecutive weeks and months and then changed to another pattern over a certain period. Thus, the feasibility of studying the association between dynamic characteristics and individuals’ traits is called into question. The characteristic cores of DMN/CEN or VN dominance, as observed with a static hierarchy in our previous study (Whi et al., 2022b), should also be reinterpreted in terms of their significance. It is easy to assume that the observed period is stationary during the resting state in terms of functional graph structure. If static data in one block were studied using percolation, we can also assume that it might have yielded a pattern of dominant voxels per study of an individual, assuming stationarity. However, our findings showed that this was not the case, using k_max_core as non-stationarity is absolute with a time resolution of 3 s. A diverse pattern of succession and transition of voxels’ temporal progression was prominent at this time resolution, and we referred to the change in pattern as a state transition. Here, state transition implies non-stationarity and underlying succession. The question of the possible representativeness of static analysis for time-varying, fluctuating, and transitioning states warrants subsequent studies from the points of hierarchy and the afferent capacity of positive and negative graphs.

State transition is currently being interrogated using complex network theory to determine its underlying mechanisms (Bianconi et al., 2015; Alet and Laflorencie, 2018; Heyl, 2018; García-Pérez et al., 2020; Nokkala et al., 2024). A growing many-body system (Khona and Fiete, 2022; Bollt et al., 2023) was the basis of the network’s hyperbolic geometry, which emerged from the many-body interactions in the simplicial complex (Bianconi and Rahmede, 2017). Brain voxels have been considered as elements or quanta in many-body interactions, which are suitable for hyperbolic embedding with scale freedom and phase transition (Bianconi and Rahmede, 2017; Whi et al., 2022a; Whi et al., 2022b). In our previous study, a many-voxel collective configuration showed abrupt transitions in k-core percolation (Huh et al., 2022; Whi et al., 2022a; Whi et al., 2022b) and abrupt (or explosive) changes in the k_max_core voxel timepoint plots (Huh et al., 2022). The former was on the configuration space, and the latter was on the temporal axis. The abrupt increase in the afferent node capacity of the directed graphs corroborated these findings in this study. Voxel hierarchy and information flow revealed intervoxel correlations and anticorrelations. This may be the essence of the dynamic criticality of the functional brain connectivity observed in the resting state, extending beyond stability or metastability (Cocchi et al., 2017). In future study, the reason why the positive graphs had recognizable state transitions and an explosive emergent rise and fall of afferent modules will be investigated, while negative graphs did not.

The combination of the third and fourth assumptions were observed pairwise waveforms of each voxel (García-Pérez et al., 2020; Huh et al., 2022; Whi et al., 2022a) act as the simplest representation of their higher-order interaction (Cinelli, 2019; Lambiotte et al., 2019; Battiston et al., 2020; Boccaletti et al., 2023) and that the pairwise-observed amplitude correlation as an observable is the sum of signals (functional connectomic dynamics) and redundancy (including inherent and measurement-related error/noise) (Pitkow and Angelaki, 2017; Luppi et al., 2023). These assumptions were implicit basics for all our analyses of dynamic functional brain graphs, such that observables like intervoxel similarity consisted of signals and redundancy (or noise/error). According to these assumptions, we removed measurement-related errors and noise, as well as inherent redundancy, by evaluating log–log plots of the degree distribution of intervoxel pairwise similarity (Whi et al., 2022a; Whi et al., 2022b). Unexpectedly, the afferent node capacity and volume capacity remained unaffected by the increase in the number of edges by several orders of magnitude (from thousands to millions) when we varied the thresholds from 0.45 to 0.7 and increased the number of edges by several orders of magnitude. Technically, this guaranteed the resilience of the information flow analysis using a topological approach/Markov modeling, and we did not need to remove the redundant weakly connected edges beforehand. Theoretically, this could also mean that the surplus edges do not contribute to the information flow or global information capacity of the graphs. Surplus edges consisted mainly of redundant edges and small amounts of errors/noises. This would make sense if the redundancy did not confound or help the information flow in the brain graphs with surplus edges. Proving or falsifying this idea requires further studies using computational modeling with population codes simulating neuron–glia complexes. Intervoxel similarity of the signals was determined solely by amplitude correlation in this investigation, and we disregarded intervoxel phase similarity and differences (Kringelbach et al., 2020). The amplitude-based similarity measures might have constrained the surplus edges to avoid contributing to the calculation of graph volume entropy or afferent/efferent capacity, as the edges were stripped of their phase information.

The thresholding of intervoxel correlations in making brain graphs to expose the skeleton of their hierarchical structures was an essential prerequisite for effective k-core percolation. In the recent reports using percolation (Nicolini and Bifone, 2016; Mastrandrea et al., 2017) for characterization of hierarchical organization, weaker edges were removed in our previous study (Huh et al., 2022; Whi et al., 2022a; Whi et al., 2022b), and we extracted scale-free cores for the following percolation. When we used the unthresholded or underthresholded data of the same individual for k-core percolation, the percolation proceeded without yielding state transitions on the k-core animation plots or any abrupt changes in the k_max_core voxels/IC composition timepoint plots. The distribution-free or scale-free characteristics of the degree distribution dictated the lower limit of the threshold. Instead, the node (voxel) number criterion, that is, 85% or more voxels, determined the upper limit of the threshold, which could maintain enough nodes to form the giant connected component. Positive correlation-based brain graphs, as well as unsigned negative correlation-based brain graphs, allowed thresholds to range from 0.45 to 0.7, within which the ranges of windows, scale freedom, edge number–total k-core proportionality, and consistency of volume entropy were conserved (Supplementary Figure S2). We set the thresholds separately for each group of subjects (a cohort) in the HCP or Kirby database (Choe et al., 2015; Huh et al., 2022), based on their positive and negative graphs.

In the 180 individuals of the HCP cohort, with 280 time bins each, outlier time bins were rare in terms of the number of voxels. Timepoint plots of both edge-scaled total k-core and volume entropy were not affected by the number of voxels (Supplementary Figure S10A). The number of edges was also not affected as well, but for different reasons. The total k-core was strictly proportional to the total number of edges and was normalized by dividing it by the number of edges. After scaling with a number of edges, comparisons were possible between any time bins within or between individuals using various edge-scaled plots, such as animated edge-scaled flag plots, edge-scaled k-core timepoint plots, and k-core map plots. Meanwhile, k_max_core plots such as stacked histogram timepoint plots and glass brain voxel/IC animation were free from any edge number effects, since they show top-tier voxels irrespective of the remaining edges after thresholding.

The volume entropy and afferent node capacity were also unaffected by the number of edges, not requiring an edge-scaling process. A redundant surplus of edges with full participating voxels less than a certain threshold (e.g., below 0.5 in the positive network in one case) did not influence the calculated values of volume entropy for all 280 time bins (Figure 3). We concluded that the volume entropy of a brain graph (per time bin) reflects the core skeleton of functional brain connectivity graphs, rather than the surplus of edges. Thus, thresholding was not necessary for assessing volume entropy or quantifying afferent and efferent capacities. However, the same thresholds were used in this investigation for both k-core percolation and directed graph construction, as well as for determining the afferent capacity to facilitate side-by-side comparisons.

As mentioned, the k_max_core (hierarchical structure) and afferent node capacity (sum of edge weights of voxels on a directed weighted graph) of positive graphs corroborated each other, revealing spontaneous module switches and suggesting resting state transitions. In unsigned negative graphs, however, the k_max_core and afferent node capacity rarely showed state transition or module switches, as did the efferent node capacity of positive graphs. The quantified timepoint plots of the afferent capacity for the negative graphs showed a much lower capacity than those of the positive graphs. The volume entropy of negative graphs also tended to be lower than that of positive graphs. For the 180 individuals in the HCP cohort, the mean number of average (over 280 time bins) edges per individual in the negative graphs (thresholds of 0.5) was 240 K compared with 490 K in the positive graphs (thresholds of 0.65). Unfortunately, we could not directly compare the measures of positive and negative graphs because these positive and negative graphs are separate entities from the same individuals. In almost all the subjects, negative graphs did not reveal state transitions or module switches. Thus, the discovery of state transitions and module switches in positive graphs was remarkable and was considered to indicate that the resting state is non-stationary.

In directed functional brain graphs as previously observed (Lee et al., 2019; Ha et al., 2020) and in this study, we found discordance between afferent and efferent flows. In our previous study, discordance in the edge matrix of afferent and efferent was observed on static functional directed brain graphs, but we could not explain this phenomenon. In this investigation, we cropped many (280 time bins per individual) dynamic directed graphs and their corresponding edge matrices for each individual. We calculated afferent and efferent node capacities from the edge capacities on the volume entropy calculation program written in MATLAB, and the calculation was normalized with the volume (Lee et al., 2019), and the brain-overlaid image was normalized to the maximum value of all the voxels of afferent and efferent node capacities in an individual. Discordance, which cannot be an artifact, prevailed with no exception. Animated efferent node capacity was less bright without discernible module exchanges on the brain-overlaid dynamic displays.

Only the positive afferent node capacity on the animated brain-overlaid (or on k_max_core stacked histogram timepoint plots) showed definitive modules and module exchanges (states and state transitions) during the resting-state fMRI acquisition session. Negative afferent nodes showed ripples and small multifocal flickering without any modules or module exchanges. Therefore, we propose that positive afferent node capacity is the source of state transition in the resting state in humans. This finding reminded us of the earlier report by Logothetis et al. (2001) that found that local field potential (LFP) was an immediate source of BOLD signals in a visual stimulus task study using monkeys in a simultaneous EEG/fMRI machine. Local field potential represents the amplitude of afferent (presynaptic) collective inputs at the synaptic buttons in the dendrites of neurons. Postsynaptic potentials were the output from the designated neurons. The difference is that Logothetis et al. (2001) used a task paradigm, not a resting-state scheme, and the LFP is not merely equivalent to afferent voxel capacity. We propose this analogy because this finding is new and encourages future computational modeling studies to address this puzzle.

Although state transition *per se* was a novel discovery enabled by sliding-window hierarchy and information flow analysis, we also observed that the voxel/IC representation allowed us to reveal the heterogeneity of voxel participation at each time bin (Huh et al., 2022) (Figure 7 and Supplementary Figure S14). This interesting observation was derived from the selection of the input, not the ROI, but the voxels being considered as independent entities. As we disregarded the topography of voxels’ BOLD signals but rather on the pairwise intervoxel correlation edges, we might also assume that voxels were independent during the 1-min-long 3-s shift time-bin window, even allowing us to disregard spatial adjacency. This is different from the situation of structural connectivity. Recent advances in hardware, central processing units (CPUs), and memory for universal Linux/Windows workstations have led to the routine use of intervoxel pair matrix computation, including eigen decomposition. When we downsampled the original 2 mm × 2 mm × 2 mm fMRI data to 6 mm × 6 mm × 6 mm or 10 mm × 10 mm × 10 mm resolution, the propensity of the edges in any individual of any cohort was half positive and half negative. After making this inadvertent observation, we could no longer reduce the fMRI data to ROIs, such as 274 parcels, without showing positive correlation bias (Lee et al., 2019; Ha et al., 2020; Whi et al., 2022a). Therefore, voxel-based calculations seemed appropriate for every subsequent investigation (Whi et al., 2022b). To facilitate the identification of voxels, ICA with a readily available algorithm was used to annotate every voxel to 7 or more ICs. The remaining voxels (a collection of smaller ICs) were unclassified.

Currently, network science or graph research has accumulated sufficient knowledge about network and graph structures with an emphasis on communities hidden in graphs using metric measures and their geometric meanings (Boguña et al., 2021; Leus et al., 2023). The communities were determined by the network/graph structures themselves (Azimi-Tafreshi et al., 2019; Shang, 2020; Young et al., 2021; Hajibabaei et al., 2023; Peixoto and Rosvall, 2023) or separately defined using biological annotations within the networks/graphs (Bazinet et al., 2023a; Bazinet et al., 2023b). Among these many, simplicial complexes for higher-order networks or synchronization (Gambuzza et al., 2021; Baccini et al., 2022), higher-dimensional hyperbolic embedding with popularity similarity (Boguña et al., 2021; Kovacs et al., 2022; Leus et al., 2023), and generalized k-core percolation (Kovacs et al., 2022) led us to the community hidden in the graphs. Instead, in our investigation, we annotated each voxel belonging to ICs defined *a priori* using ICA outputs.

The trajectories of voxels belonging to the same ICs were surprisingly heterogeneous for both spatiotemporal deployments of k-core and of afferent capacity. Voxels took turns performing their jobs of emergent construction of modules, rising to the top of the hierarchy during the short 15-min period (Figure 7). Edge-scaled k-core was grossly similar but differed in terms of the contours of the collective trajectory maps (Supplementary Figure S1). The afferent capacity of voxels exhibited an on-and-off pattern of module formation and switching between ICs, whereas the efferent capacity did not (Figure 6; Supplementary Video S7; Supplementary Figure S12). The afferent and efferent capacities of voxels and their module-forming characteristics were initially assumed to form a hierarchical structure of edge composition in a collective, allowing the top-tier voxels to climb up to reach the k_max_core. This naïve expectation was not accurate regarding the role of the efferent capacity, as it was fairly homogeneous despite spatiotemporally scattered small flickering (Figure 6 and Supplementary Video S7). In a separate analysis of negative graphs in the form of unsigned graphs, we did not observe prominent modules nor top-tier voxels/ICs with any transitions, even in terms of afferent capacity. Thus, we suggest that the afferent node capacity of positive graphs contributes to hierarchical edge characteristics. If so, we needed to survey whether one belongs to another; that is, the two spatiotemporal trajectories of hierarchy or afferent capacity would be inclusive or overlapping if they were plotted together (Supplementary Figure S14). For any voxel on the surface or three-dimensional (3D) plots, no relationship was observed between the two measures, the k-core and the afferent capacity of the positive graphs. They looked varied on their own on their axes of abscissa and ordinate. One was not replaceable by the other. We presumed that the underlying mechanism behind their spatiotemporal progression needs to be further scrutinized if we want to understand the nature of state transition on fMRI.

Our investigation differed from previous studies that used various centrality measures in that those studies examined the structure of the networks, whereas we paid attention to the voxels themselves. Their results revealed that the degree distribution represented global graph characteristics but did not accurately reflect the voxel entities in their structural graphs. This created a difference in the interpretation of their various centrality measures, such as degree centrality, betweenness centrality, eigenvector centrality, and PageRank centrality (Curado et al., 2023), compared to our k-core values (Whi et al., 2022b). Interestingly, our volume entropy/afferent node capacity works by taking advantage of the fact that eigen decomposition is equivalent to the asymptotic measure of a random walker job, regardless of its variations of adaptive signed, return, quantum or hypergraphs (Carletti et al., 2020; Curado et al., 2023; Sotero and Sanchez-Bornot, 2023). It would be interesting to determine whether any relationship exists between the afferent/efferent node k-core measure and other centrality measures on the separated afferent and efferent directed networks we produced in this investigation. This comparison study is underway, and the pilot results are included as Supplementary Figure S15. Degree and strength animations were similar to k-core animation, but betweenness centrality was different. Top-tier voxels’ behavior on the stacked histogram animation plots would show their capability to reveal states and state transitions, although there are no reasonable criteria that exist to define top-tier voxels. A less popular, rich club coefficient would avail a similar representation of centrality as percolation, but has not been investigated yet.

Our findings raise the following questions: The first refers to the nature of state transition, revealed by an observation here using resting-state fMRI. fMRI observables were analyzed easily using (1) a classic statistical physics method (k-core percolation) and (2) the construction of directed weighted graphs based on a detection scheme of topological invariants of graphs. Currently, there is no plausible explanation for these “state transitions,” which were observed universally between individuals and even within sessions in an individual (Huh et al., 2022). The question of what caused these state transitions has not yet been answered, but clues include (1) non-linear dynamic interpretations ranging from criticality and self-organizing characteristics of neurons in the literature (Shew and Plenz, 2013; Hesse and Gross, 2014; Zimmern, 2020), represented as voxels in our investigation; (2) large-scale neuronal theory, recently refined as a neuronal communication system (Avena-Koenigsberger et al., 2017; Hahn et al., 2019; Tian and Sun, 2022) or voxels’ communication dynamics in our investigation; and (3) information theory perspectives for complex networks (Anand and Bianconi, 2009), von Neumann entropy and its transition interpretation (Minello et al., 2019; Krohn et al., 2023) or complexity entropy, such as Kolmogorov interpretation (Mateos et al., 2018), or a topological invariant named graph volume entropy (Lim, 2008; Lee et al., 2019) in our investigation. Further investigation is underway to explore these clues as time allows, as it is a very time-consuming process for computation.

The spatiotemporal trajectories gained momentum for visualization and quantification in our investigation. Once traced, these outputs can be inserted into refined computational models for dynamic functional brain voxels with higher spatiotemporal resolution along discrete time-bin axes. Recent introduction of graph neural networks (GNNs) or their improved versions, such as the graph isomorphism network with relational updates or spatiotemporal GNN, may allow comprehensive modeling of normal states’ temporal progression (Cui et al., 2023; Li et al., 2023). Specifically, time convolution over the spatial self-attention block methods may aid in constructing norms for healthy individuals’ resting-states on fMRI (Thapaliya et al., 2025a; Thapaliya et al., 2025b). The possibility of explaining spontaneous or stimulus task-associated resting-state changes in humans, whether normal; sleeping; conscious, sedated, semi-conscious, or comatose; or in the states of disorder or disease during their course or rehabilitation after successful or unsuccessful treatments, is an issue to be investigated soon.

In conclusion, we have two distinct methods for producing one spatiotemporal deployment of hierarchical realization of functional brain graphs (k-core images on animation) and producing another spatiotemporal realization of normalized afferent/efferent node capacity. These two methods were derived from each discipline and model. Exact computing was reproducible in calculations without any inter-operator bias, across all the chosen thresholds. However, there appeared to be a narrow window enabled the observation of state transitions on the k_max_core or a wider window that allowed for module exchanges on afferent node capacities.

## Data Availability

The data used in this study were obtained from the Human Connectome Project (HCP) database (https://www.humanconnectome.org/), WU-Minn Consortium. And the codes generated for this study are available through a standard material transfer agreement with POSTECH for academic and nonprofit purposes from the corresponding author upon reasonable request.

## Footnotes

^1^ www.humanconnectomeproject.org
