# Supplementary material for: Both k-core percolation and directed graph analysis revealed succession and transition of voxels’ spatiotemporal progress on dynamic correlation resting-state fMRI
Source: Front Hum Neurosci. 2025 Apr 16;19:1543854. doi: 10.3389/fnhum.2025.1543854 (PMC12040893; doi:10.3389/fnhum.2025.1543854)
Supplement: Supplementary file 13 [file Data_Sheet_1.pdf]

Supplementary Materials

List of Supplementary Figures

Supplementary Figure 1. The ability to discover the hierarchical top tier voxels according to the varying thresholds, which are the same for all the time bins per individual, to make the preprocessed input data of pairwise intervoxel amplitude correlations.

Supplementary Figure 2. The criteria for acceptable brain graphs, positive and unsigned negative, were set as follows: 1) prescreening with the number of nodes and scale-freeness of the degree distribution according to the varying thresholds and 2) the consequences of thresholding on volume entropy, modules on afferent node capacity, state transitions and the relationship between the number of edges and total coreness k.

Supplementary Figure 3. Characteristic measures on the graphs and their animations and timepoint plots of a representative individual for functional brain graphs of positive (and unsigned negative) intervoxel correlations.

Supplementary Figure 4. Reproducibility of designating 'state transition' by counting the numbers by one operator among the authors using the positive graphs.

Supplementary Figure 5. Glass brain animation plots and stacked histogram timepoint plots on undirected positive graphs and afferent capacity timepoint plots showing module formation and switches on directed positive graphs: intermediate pattern between no transition and 'the state transition before and after half the period'.

Supplementary Figure 6. Glass brain animation plots and stacked histogram timepoint plots on undirected positive graphs and afferent capacity timepoint plots showing module formation and switches on directed positive graphs: examples of typical state transitions.

Supplementary Figure 7. Glass brain animation plots and stacked histogram timepoint plots on the undirected positive graphs and afferent capacity timepoint plots showing module formation and switches on the directed positive graphs: an individual showing too-frequent transitions.

Supplementary Figure 8. State fluctuation of synchronized voxels on glass brain animation plots and stacked histogram timepoint plots and on afferent capacity timepoint plots.

Supplementary Figure 9. Asymmetry of module composition of states in three subjects showing frontal alternating (A,B), recurrently appearing in left frontal area (C,D), and left cerebellar asymmetry (E,F) patterns.

Supplementary Figure 10. Volume entropy (A) and afferent node capacity (B) with timepoint plots and their corresponding coreness k (C) and k_{max}core timepoint plots (D).

Supplementary Figure 11. The relationships between the volume entropy and total coreness k of the time bins of the positive and negative graphs of the individuals are presented in Suppl. Fig. 10.

Supplementary Figure 12. Example of voxels/IC composition timepoint plots and their afferent and efferent node capacity animation maps of positive graphs in an individual (#128632).

Supplementary Figure 13. Back-to-back representation of afferent node capacity of voxels/IC timepoint plots of unsigned negative graphs and their corresponding positive graphs in representative individuals, followed by their matching k_{max}core stacked histogram timepoint plots.

Supplementary Figure 14. Trajectory tracing of a voxel along the time-bin progress of its own coreness k values and afferent node capacity

Supplementary Figure 15. Glass brain animation plots and stacked histogram timepoint plots of k_{max}core voxels and other graph metrics

List of Supplementary Movies

Supplementary Movie 1. Animation plots for Figure 4A. Glass brain and stacked histogram of k_{max}core voxels.

Supplementary Movie 2. Animation plots for Figure 4C. Glass brain and stacked histogram of k_{max}core voxels.

Supplementary Movie 3. Animation plots for Figure 4E. Glass brain and stacked histogram of k_{max}core voxels.

Supplementary Movie 4. Animation plots for Figure 4G. Glass brain and stacked histogram of k_{max}core voxels.

Supplementary Movie 5A. MRI-overlayered coreness k maps of positive graphs on animation for Figure 5A.

Supplementary Movie 5B. MRI-overlayered coreness k maps of unsigned negative graphs on animation for Figure 5A.

Supplementary Movie 6A. MRI-overlayered coreness k maps of positive graphs on animation for Figure 5B.

Supplementary Movie 6B. MRI-overlayered coreness k maps of unsigned negative graphs on animation for Figure 5B.

Supplementary Movie 7A. MRI-overlayered afferent node capacity maps of positive graphs on animation for Figure 6A

Supplementary Movie 7B. MRI-overlayered efferent node capacity maps of positive graphs on animation for Figure 6B

Supplementary Movie 7C. MRI-overlayered afferent node capacity maps of unsigned negative graphs on animation for Figure 6C

Supplementary Movie 7D. MRI-overlayered efferent node capacity maps of unsigned negative graphs on animation for Figure 6D

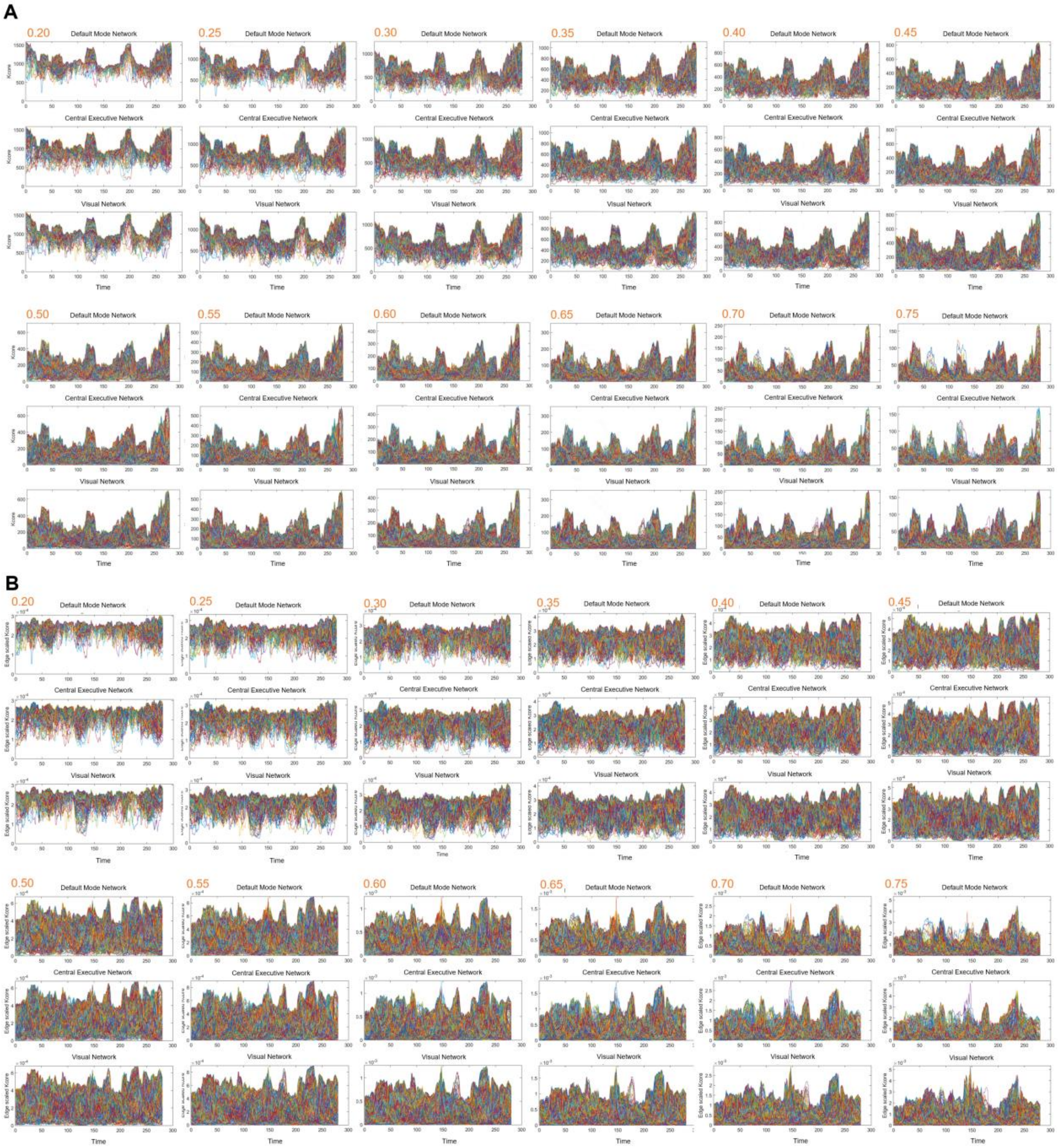

C

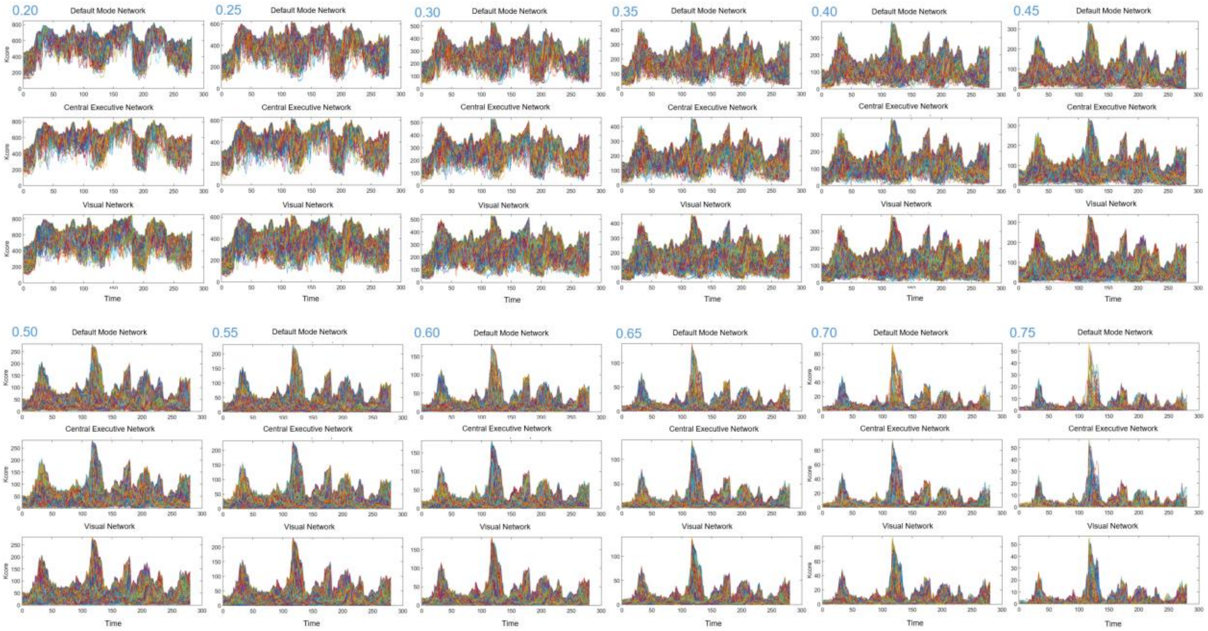
0.20
0.25
0.30
0.35
0.40
0.45
0.50
0.55
0.60
0.65
0.70
0.75
Default Mode Network
Central Executive Network
Visual Network
Kcore
Time

D

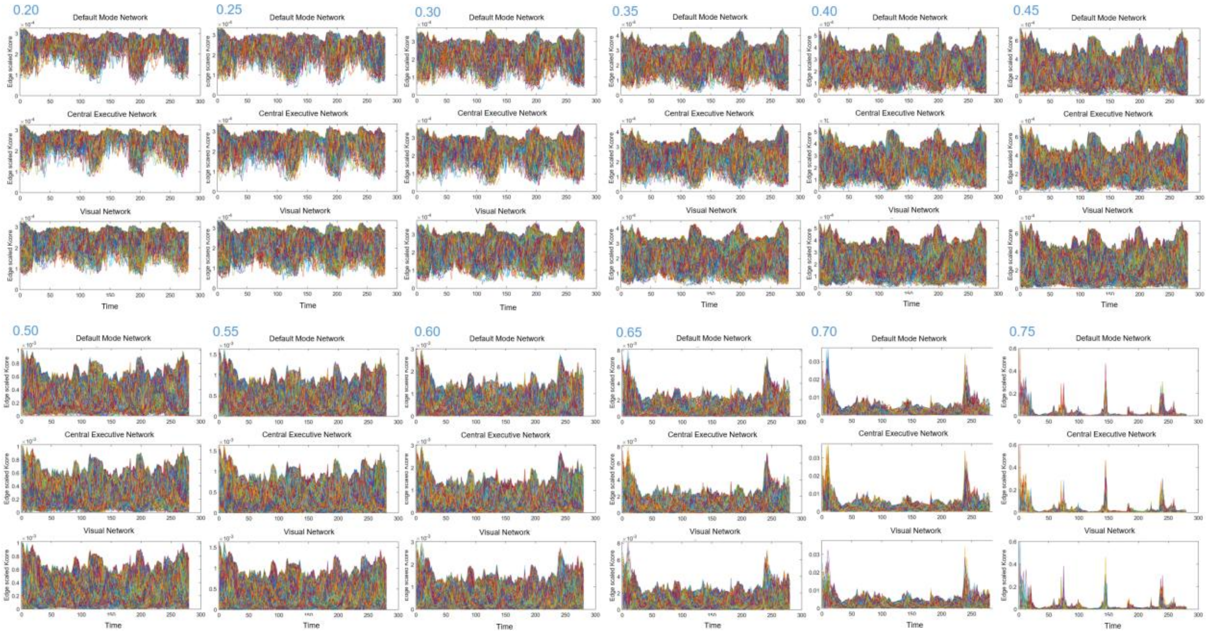
0.20
0.25
0.30
0.35
0.40
0.45
0.50
0.55
0.60
0.65
0.70
0.75
Default Mode Network
Central Executive Network
Visual Network
Edge scaled Kcore
Time

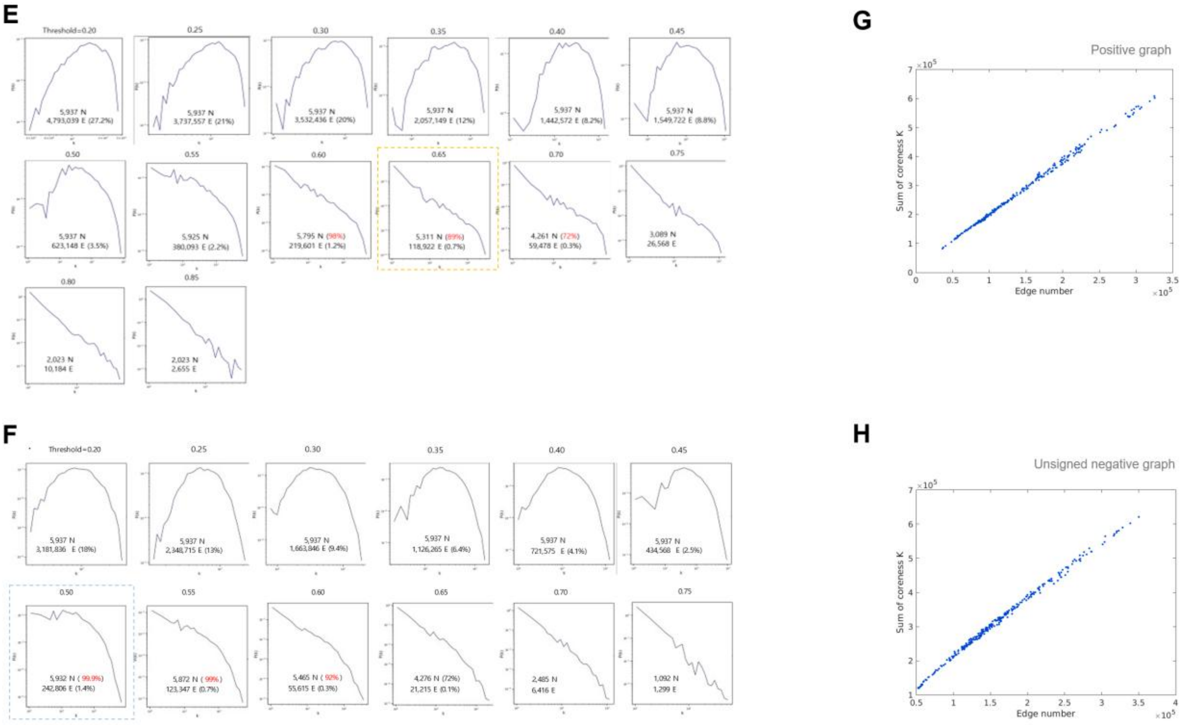

Supplementary Figure 1. The ability to discover the hierarchical top tier voxels according to the varying thresholds, which are the same for all the time bins per individual, to make the preprocessed input data of pairwise intervoxel amplitude correlations.

Individuals' intervoxel correlations of 5,937 voxels were assumed to contain the edges showing the characteristic scale-free-ness of their degree distribution and the surplus edges making the distribution have median/mode/mean structures. Varying the thresholds disclosed the degree-distributions of thresholded intervoxel edge weights (correlations) and these correlation matrices and their adjacency matrices were used as input for k core percolation. Thresholds were varied from 0.20 to 0.75 (total 12 thresholds) and k core percolation yielded timepoint plots, MRI-overlayed coreness k animation map plots, and stacked histogram plots of k_{max}core.

A. Voxels timepoint plots of coreness k values in positive graphs of the case identification #100206. Each IC contained 732 voxels for DMN, 351 for SN, 363 for DAN, 682 for CEN, 483 for SMN, 289 for AN, 1,104 for VN and 2,691 for the unclassified. In this figure, timepoint plots of DMN, CEN, and VN were only displayed. The coreness k trajectory timepoint plots showing the coreness k values (Kcore) were plotted.

B. The voxels' coreness were divided (edge-scaled) by the total number of edges per time

bins. The same threshold used for all the time bins let the edges vary in number of several orders (several thousands to millions) and thus the edge-scaled voxel Kcores showed less variation than the non-scaled ones. Stacked histograms of the thresholds from 0.45 to 0.75, state transitions were clearly shown (Figure 2) and the timepoints of state transition were almost at the same points and the same duration.

C. Voxels timepoint plots of coreness k values in negative graphs of the case identification #100206. The contained voxels in each IC are the same with positive graph (A). The coreness k trajectory timepoint plots showing the coreness k values (Kcore) were plotted.

D. The voxels' coreness k were divided (edge-scaled) by the total number of edges per time bins in negative graph. The same threshold used for all the time bins let the edges vary in number of several orders (several thousands to millions) and thus the edge-scaled voxel coreness k showed less variation than the non-scaled ones.

E. In positive graphs, degree distribution showed typical changes according to the varying thresholds. For each threshold, number of voxels remaining were from 5,937 to 2,023 and number of edges from 4.9 million to two thousand. Percent indicators in the boxes of the thresholds 0.60, 0.65, and 0.70 in red showed the threshold of 0.70 are not allowed as the number of nodes was less than 85% (set threshold in this study). Scale-freeness were noted from the threshold of 0.55 to the higher ones. Individual variations between 180 subjects were noted but in a few and we could set the universal threshold of 0.65 for the positive graph studies in this HCP cohort.

F. In unsigned negative graphs, degree distribution changes were the same as the positive graphs. Red percent indicators say that we needed to use the threshold equal or lower than 0.60. Edge numbers ranged from 3.1 million (threshold 0.2) to 1,229 (threshold 0.75). Scale freeness was found at the threshold 0.5 or higher. Changing pattern according to the thresholds was similar to that of positive graphs. After looking at the changing pattern of scale-freeness and the remaining number, we chose the threshold 0.50 for the negative graphs in this HCP cohort.

G. The total coreness k were highly correlated with the number of edge number in positive graph. Therefore, to understand the spatiotemporal progress of voxel's hierarchy over time, coreness k was analyzed by dividing it by the number of edges in each window. This means that the spatiotemporal progression is not reflecting the difference in the number of edges.

H. The total coreness k were highly correlated with the number of edge number in positive graph. Therefore, the coreness k was analyzed by dividing it by the number of edges in unsigned negative graph.

HCP: human connectome project, IC: independent component, DMN: default mode network, SN: salience network, DAN: dorsal attention network, CEN: central executive network, SMN: sensorimotor network, AN: auditory network, VN: visual network

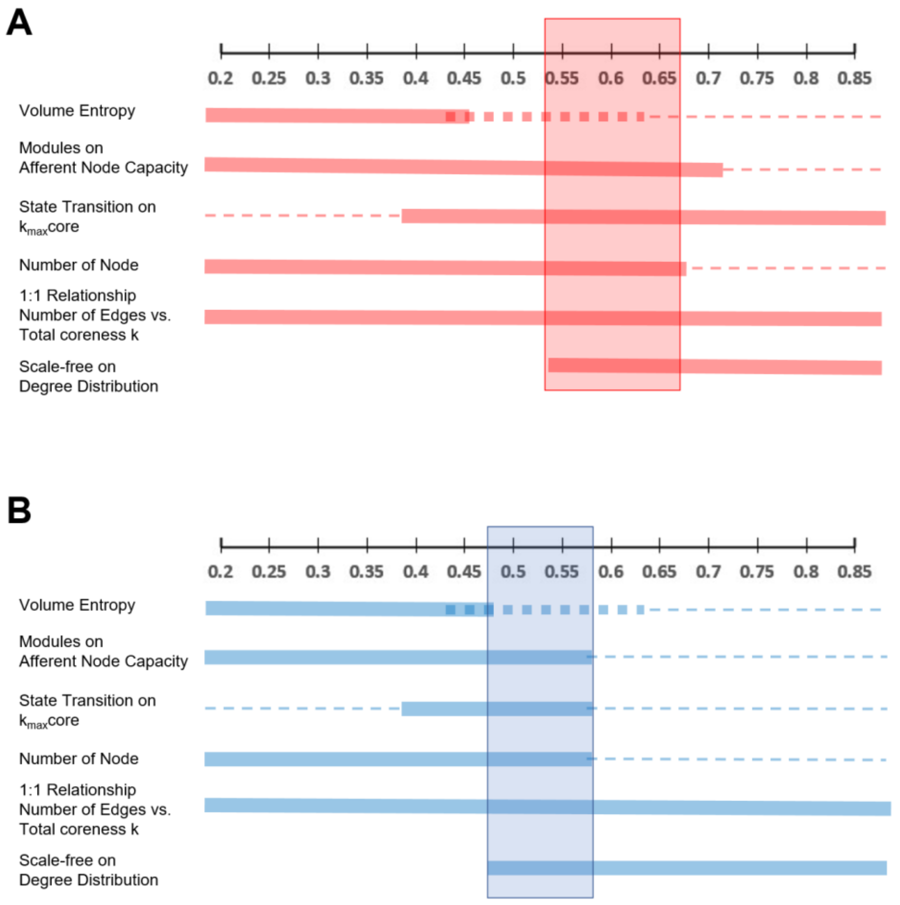

Supplementary Figure 2. The criteria for acceptable brain graphs, positive and unsigned negative, were set as follows: 1) prescreening with the number of nodes and scale-freeness of the degree distribution according to the varying thresholds and 2) the consequences of thresholding on volume entropy, modules on afferent node capacity, state transitions and the relationship between the number of edges and total coreness k.

A. In an example of positive graph of an individual (#100206), number of nodes more than 85% of 5,937 voxels were guaranteed until the threshold was 0.65 and degree distribution was scale-free (linear descent on log-log plot of degree-distribution plots) for all the time-bins of brain graphs whose thresholds were equal or higher than 0.55. Post-hoc observation of volume entropy showed the same values for the graphs with the thresholds ranging from 0.2 to 0.45 and with higher thresholds, slowly decreasing till 0.6 and then linearly decreased according to the total edge numbers of each time-bin graphs. Directed graph and its afferent node capacity revealed exactly the same modules (voxels/IC composition) and module exchanges until the threshold of 0.7. K core percolation results, especially k_{max}core stacked histogram timepoint plots yielded the evidence of state transitions, the same from the threshold 0.4 to the last one of 0.85. One-to-one relationships between the total number of

edges and the total coreness k of the brain graphs per time bins maintained all over the thresholds.

B. In unsigned negative graph of this person, number of nodes larger than 85% were until the threshold of 0.55. Scale-freeness was observed when the threshold was equal to or higher than 0.5. Volume entropy were the same until the thresholds were less than 0.5, and modules were found until the threshold increased to 0.55. Implicit evidences of state transition were barely observed when the thresholds were between 0.4 and 0.55. 1:1 relationship between number of edges and total coreness k maintained all over the thresholds.

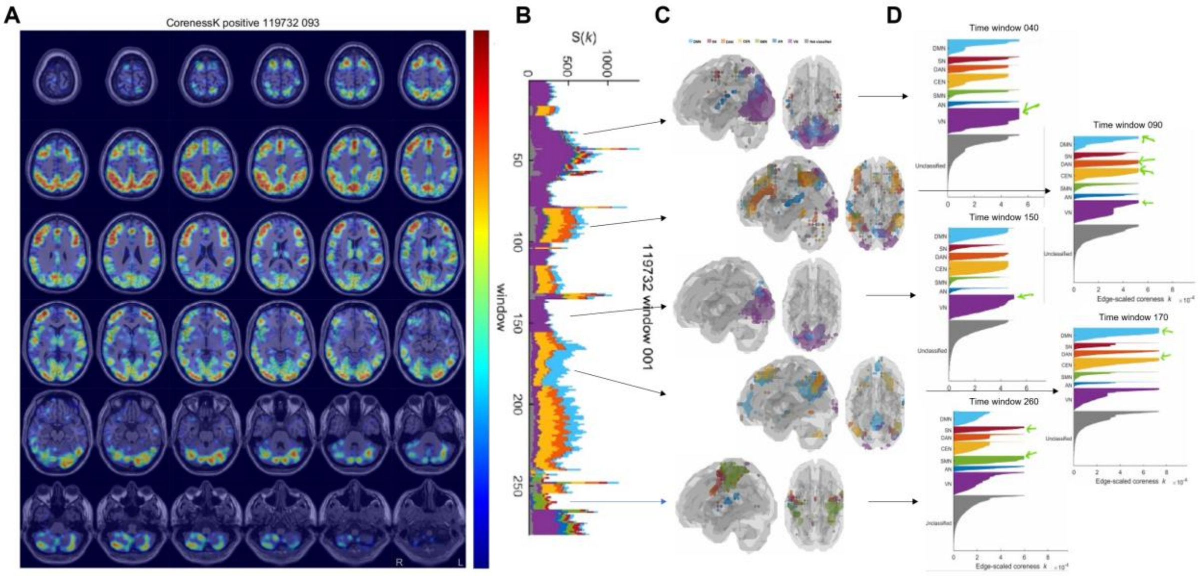

Supplementary Figure 3. Characteristic measures on the graphs and their animations and timepoint plots of a representative individual for functional brain graphs of positive (and unsigned negative) intervoxel correlations.

A. Coreness k values of all the voxels were divided by total number of edges to yield edge-scaled coreness k values. These edge-scaled coreness k values were overlayed on the 36 slices MRI in radiologic convention and were displayed as avi file on animation. The 93th time bin snapshot image was displayed as an example.

B. Timepoint plots of stacked histogram of an example case showed the states of clustered time bins with similar composition of k_{max}core voxels/IC compositions, which were colored to clearly visualize the abrupt changes of the voxels/IC compositions at certain points. We called this time-bin switching of voxels/IC composition as ‘state transition. In this case, at least 11 state transitions were recognized.

C. Glass brain images of left lateral and superior views were matched with the time-bin points of k_{max}core stacked histogram. The uppermost show VN dominant with scanty companion ICs state, the next one DMN/DAN/CEN major and VN/AN minor, the third VN dominant, the fourth DMN/CEN main, and the lowest show SMN with SN.

D. On the animated flagplots (snapshots here for explanation), small green hand-drawn arrows indicate the k_{max}core voxels (and their ICs) on the top-tier (rightmost tier).

HCP: human connectome project, IC: independent component, DMN: default mode network, SN: salience network, DAN: dorsal attention network, CEN: central executive network, SMN: sensorimotor network, AN: auditory network, VN: visual network

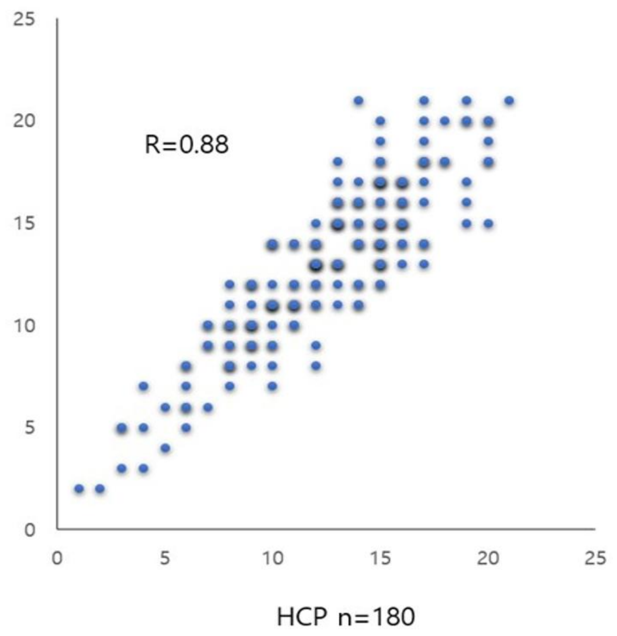

Supplementary Figure 4. Reproducibility of designating 'state transition' by counting the numbers by one operator among the authors using the positive graphs.

Even taking into consideration of the operator's learning curve and no explicit definition of state transition on stacked histogram of k_{max}core voxels/IC composition a priori, intra-operator reproducibility was acceptable (Spearman's rho of 0.88). Later on, the state transition were defined as 1) abrupt change of k_{max}core voxels/IC composition within one time bin, 2) state should be at least one or more time-bin duration, 3) conservatively, voxels' fraction changes of IC composition did not constitute a state, meaning that different states are defined more importantly by IC composition changes with or without fraction changes, and, nevertheless, 4) in ambiguous occasions, heuristic decision whether this or that abrupt change deserves 'state transition' was allowed. Based on the third and the fourth oracles, negative graphs lost many of the state transitions because the subtle changes were not so abrupt nor so clear as those of positive graphs. Interestingly, the stacked histogram of k_{max}core voxels/IC composition, once mixed blindly of positive and negative graphs, we could easily dissociate the positive and negative groups.

A

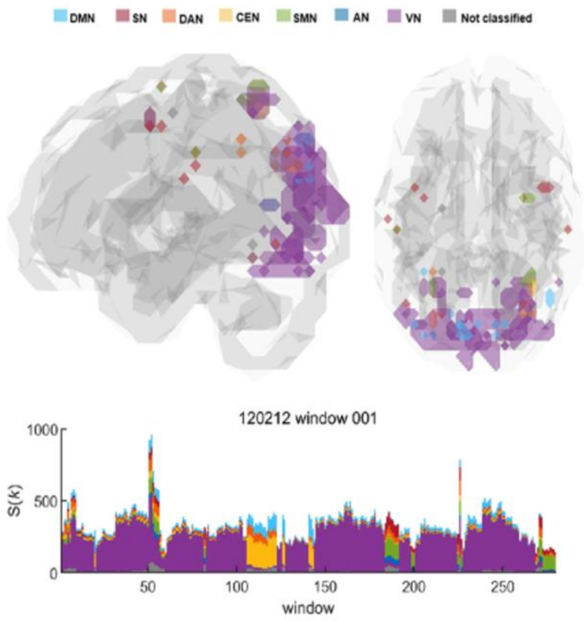
DMN
SN
DAN
CEN
SMN
AN
VN
Not classified
120212 window 001
S(k)
window

B

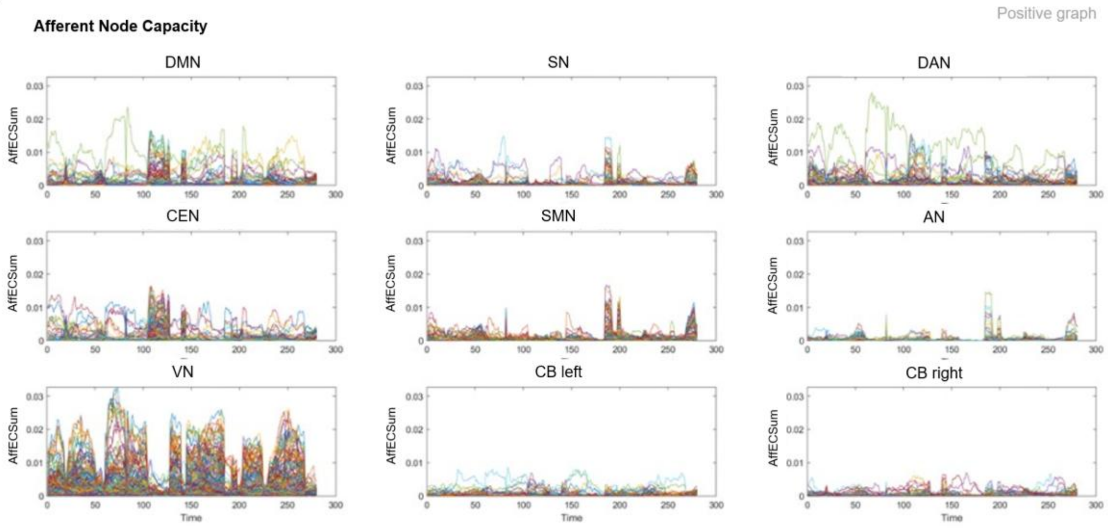
Afferent Node Capacity
Positive graph
DMN
SN
DAN
CEN
SMN
AN
VN
CB left
CB right
AffECSum
Time

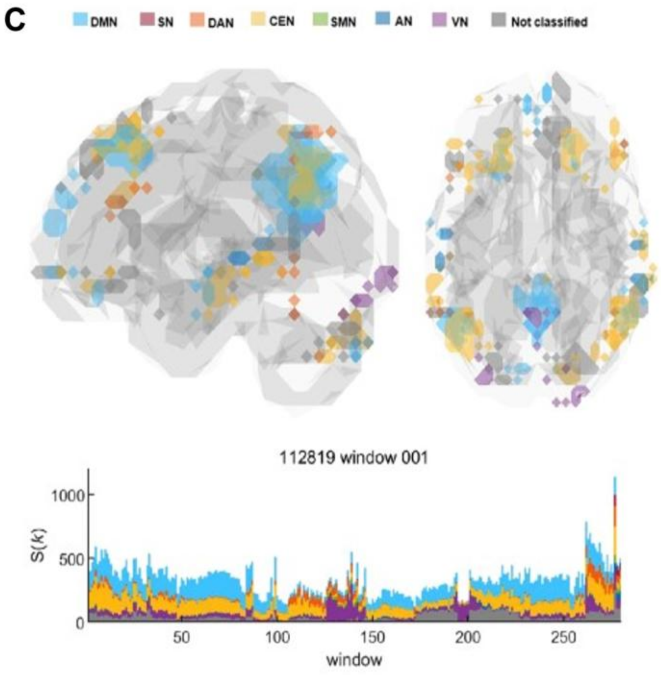

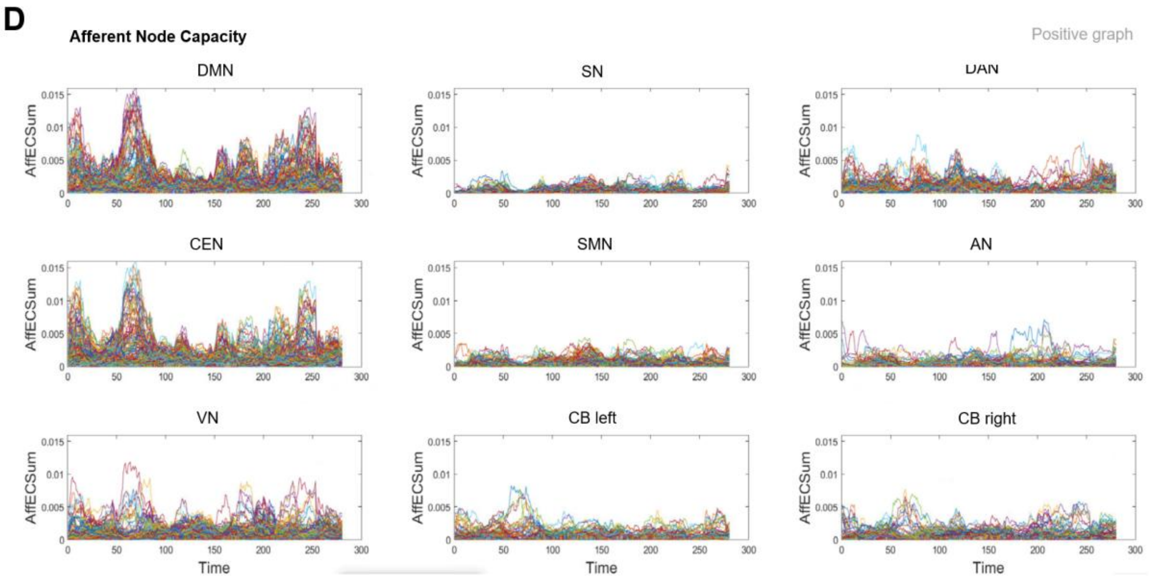

Supplementary Figure 5. Glass brain animation plots and stacked histogram timepoint plots on undirected positive graphs and afferent capacity timepoint plots showing module formation and switches on directed positive graphs: intermediate pattern between no transition and 'the state transition before and after half the period'.

A. In this individual, VN voxels dominated throughout all the time-bins of the k_{max}core plots except at the middle of the period. After 100th time-bin, DMN/DAN/CEN took over the top

tier on the hierarchy for some while and SN/SMN/AN did just before 200th time bin for a short while. Single IC (VN) dominated then for another several minutes while yielding to its combinatorial competitors for moments and took the hierarchical supremacy again.

B. On afferent node capacity plots of this individual, VN voxels dominated every time bin except time bins after 100th and before 200th. After 100th time bin, DMN, CEN and DAN voxels filled the gap between the VN dominances, and just before 200th time bin, SN, SMN and AN joined to fill another hierarchical supremacy.

C. In another individual, DMN/CEN voxels dominated almost all the time-bins of the k_{max}core plots. However, in the middle of the period (between100th and 150th time-bins), DAN/CEN first and VN plus other smalls took charge in the top tier of the hierarchy. Around 200th time bin VN alone occupied the supremacy.

D. In afferent node-voxel capacity plots of this individual, DMN and CEN voxels showed almost the same pattern of temporal progress while DAN joined intermittently in part. Grossly similar to the findings on the k_{max}core plots, however, the prominent state transition to DAN or VN between 100th and 150th of were not clear on the afferent capacity plots.

DMN: default mode network, SN: salience network, DAN: dorsal attention network, CEN: central executive network, SMN: sensorimotor network, AN: auditory network, VN: visual network

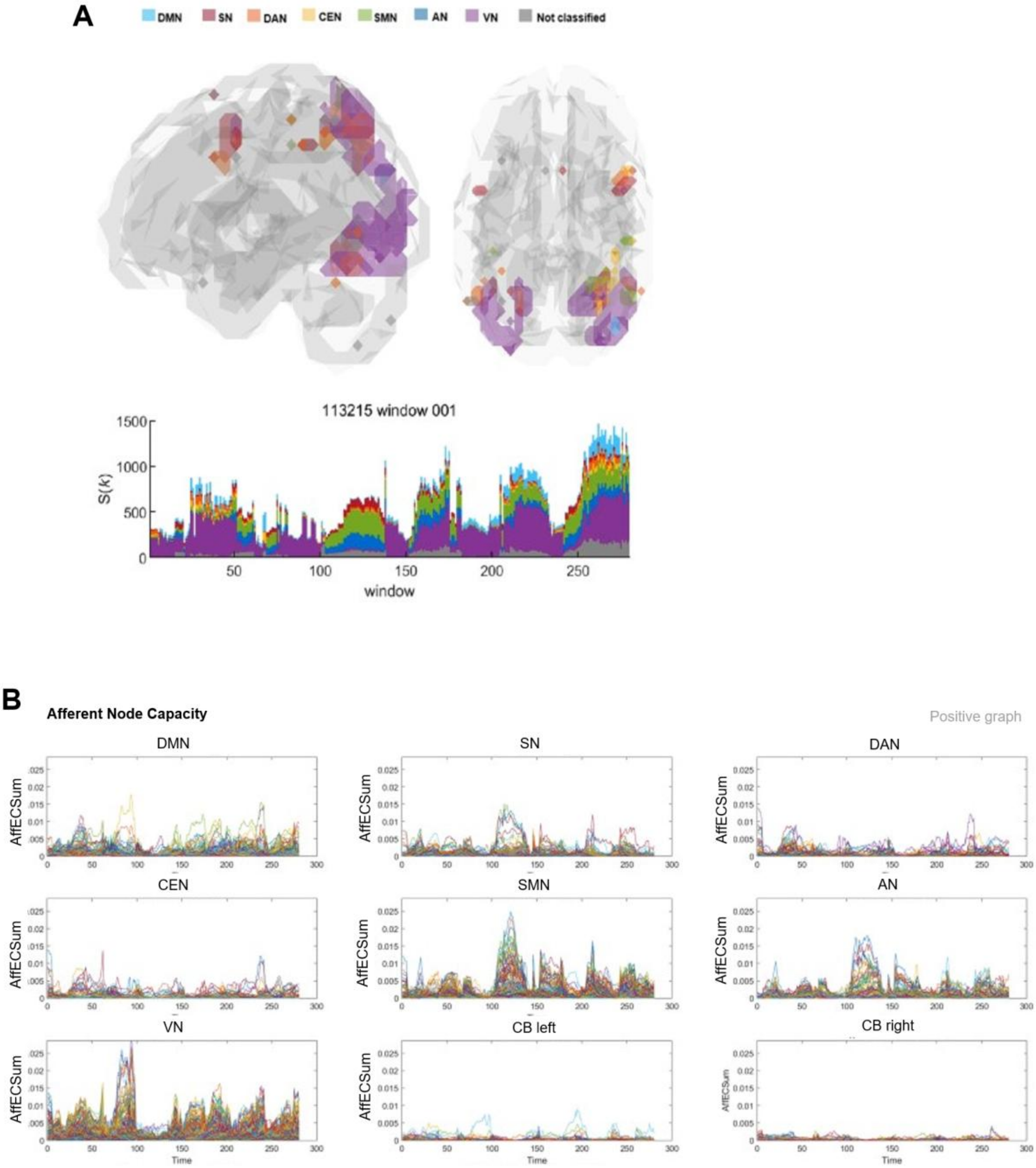
A
DMN
SN
DAN
CEN
SMN
AN
VN
Not classified
113215 window 001
S(k)
window
B
Afferent Node Capacity
Positive graph
DMN
SN
DAN
CEN
SMN
AN
VN
CB left
CB right
AffECSum
Time

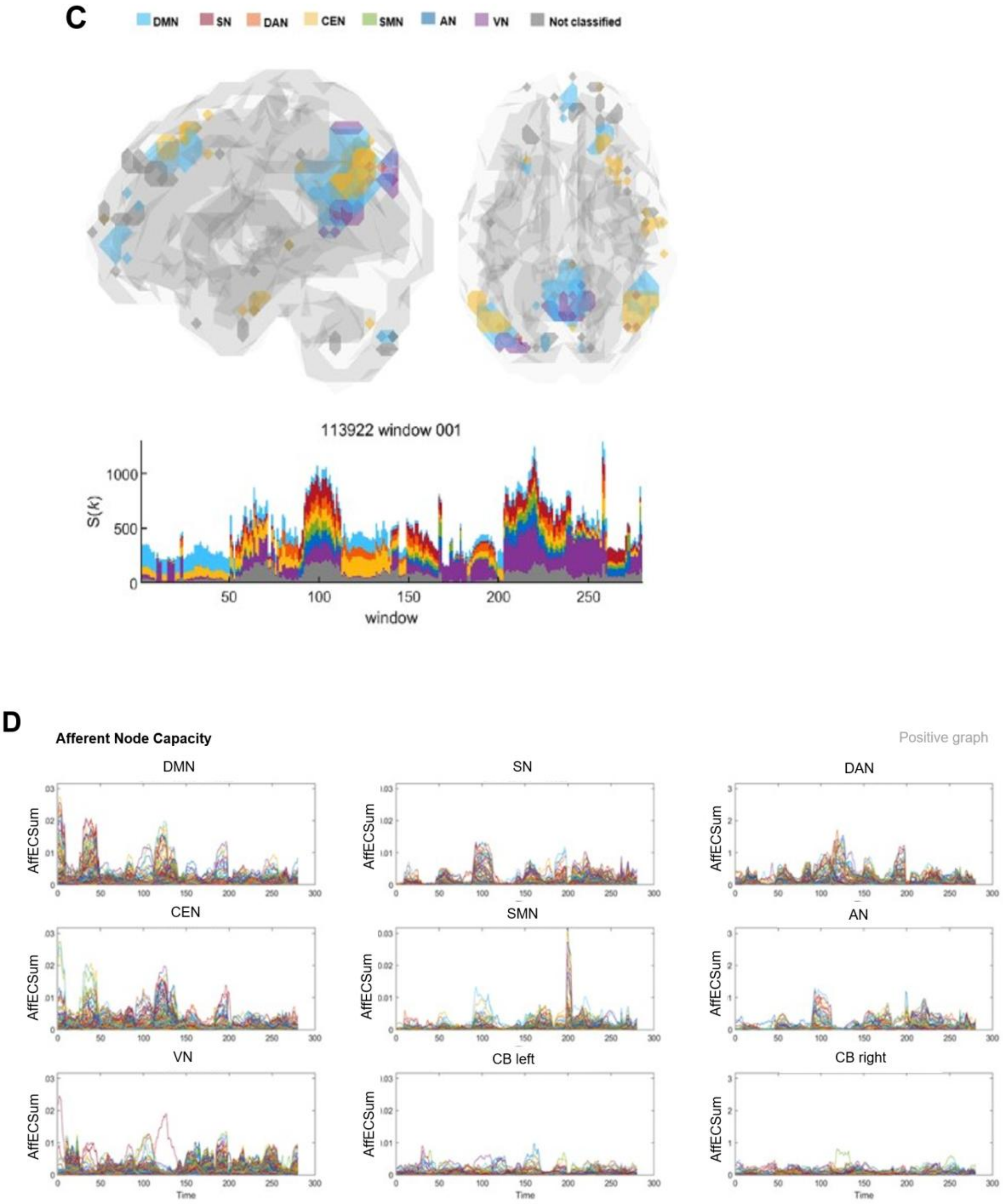

Supplementary Figure 6. Glass brain animation plots and stacked histogram timepoint plots on undirected positive graphs and afferent capacity timepoint plots showing module formation and switches on directed positive graphs: examples of typical state transitions.

A. In this individual, state transition was mostly from VN and its allies to SMN/AN/SN and back to VN dominance. Unlike frequent transitions from DMN/CEN to VN and from VN to DMN/CEN, this type of transition from VN to SMN was less frequent. During the period between 80th and 150th time bins, SMN/AN/SN co-dominance was surrounded by preceding VN and following VN dominances, and sharp transition in-between was easily recognized.

B. On afferent node-voxel capacity plots of this individual, characteristic absence of VN module and replacing SMN/AN/SN co-modules was noted during the period between 100th and 140th time bins.

C. In another individual, state transition was shown from DMN/CEN to VN and back at the earlier part. Around 50th time bin, DMN/CEN dominant state was replaced by all-modules participation, called distributed (ICs) state, which reached 90th time bin, constituting rainbow-type collective modules. This rainbow/distributed dominance was followed by DMN/DAN/CEN at 110th time bin. Several state transitions followed from the distributed to VN, VN to DMN/DAN/CEN/VN, to very short DMN/CEN, and then rainbow/distributed states. Many state transitions could be recognized like this in almost all the 180 individuals. This was the source of counting the numbers of state transitions on k_{max}-core plots (Suppl. Figure 4)

D. On afferent node capacity plots of this individual, between 25th and 50th time bins, and only DMN/CEN were conspicuous with the vacancy of VN and others. Around 100th time bin, reminding distributed module of k_{max}core plots, all the modules including the unclassified showed up. Rainbow-type distributed dominance on k_{max}core plots were called 'unison' not orchestration or symphony.

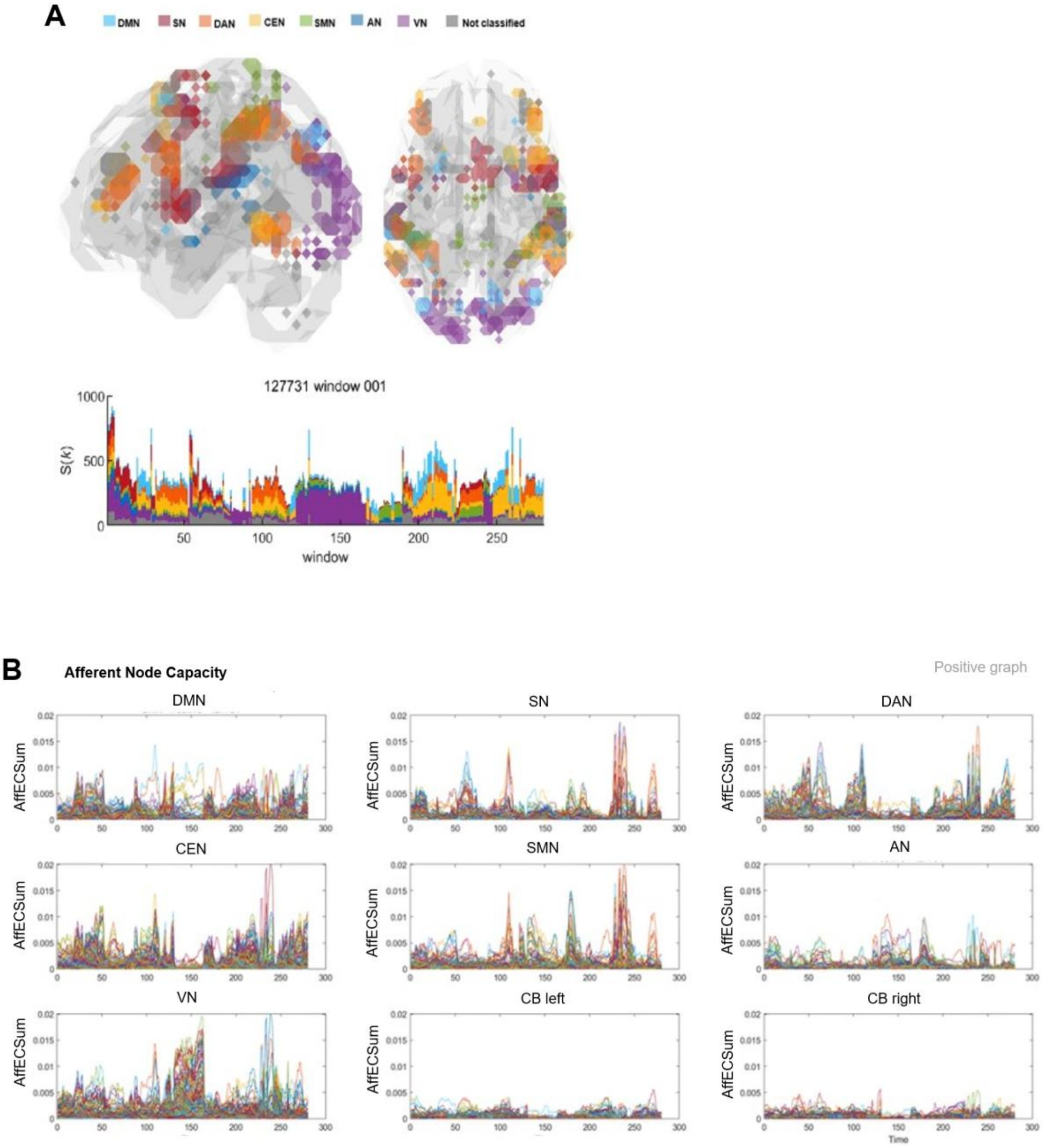

Supplementary Figure 7. Glass brain animation plots and stacked histogram timepoint plots on the undirected positive graphs and afferent capacity timepoint plots showing module formation and switches on the directed positive graphs: an individual showing too-frequent transitions.

A. In usual real cases like this individual, k_{max}core plots were more flamboyant. Stationary state sustained for variable duration even from one (3 seconds) to several tens (e.g. 50 seconds) of time bins. In this casen, state transitions could have been defined in many ways as arbitrarily as the investigators wanted. However, based on final heuristic decision, considering that stacked histogram plot consisted of the numbers of k_{max}core voxels colored

according to IC compositions, this individual was found to have 17 state transitions. Flamboyance in state progress and transition and the definition of state transition still remained a challenge.

B. Afferent node-voxel capacity of this individual showed slim, sharp, rapidly changing, and differently gathering and dismantling of voxels on every instance of observation of time bins. Nevertheless, during the period between 125th and 170th time bins when VN dominance was prominent on the k_{max}core plots, VN/SMN/AN modules were built temporarily. Then, interestingly VN module disappeared suddenly and DMN/CEN filled the vacancy and replaced VN.

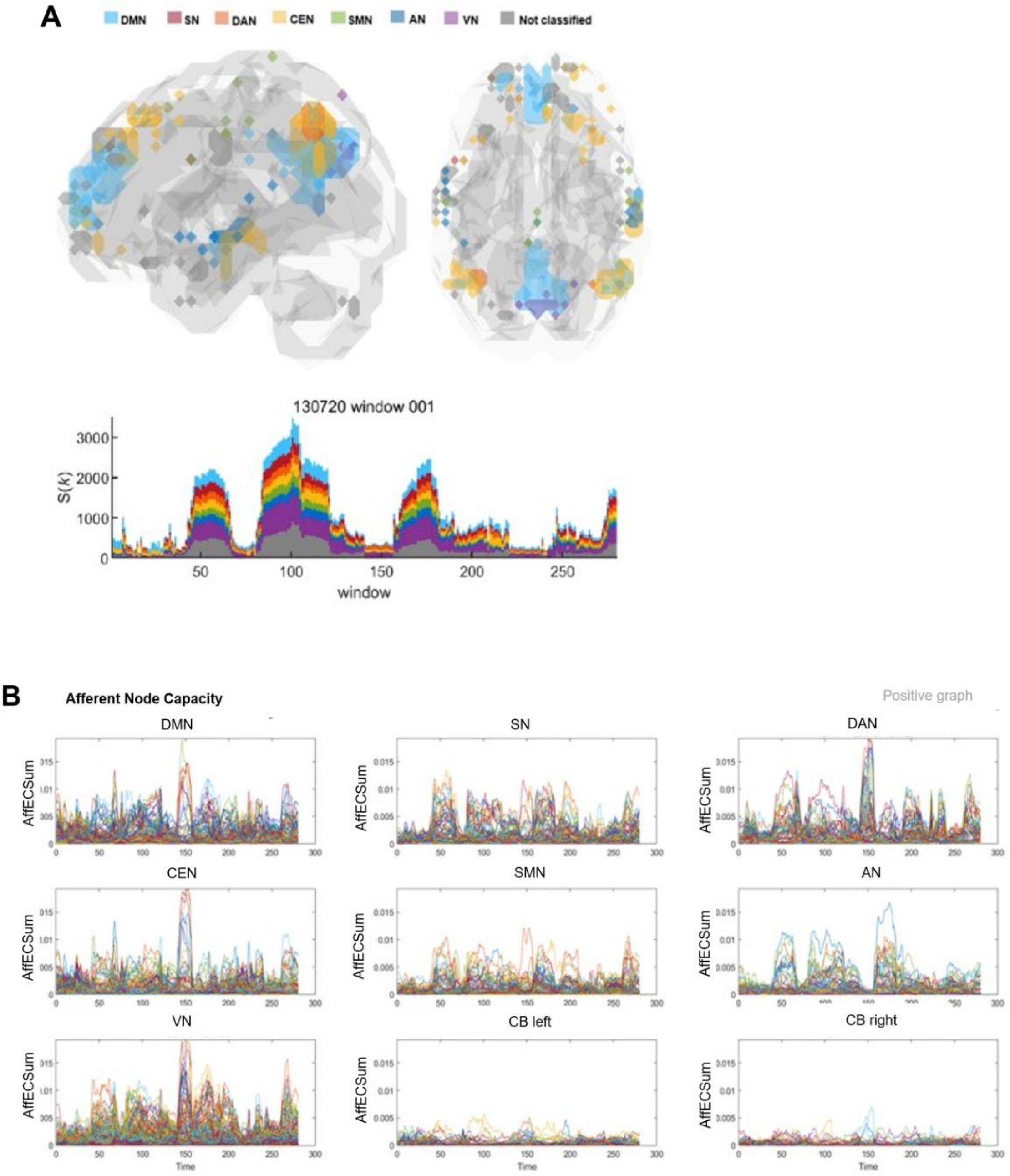

Supplementary Figure 8. State fluctuation of synchronized voxels on glass brain animation plots and stacked histogram timepoint plots and on afferent capacity timepoint plots.

A. In this individual, k_{max}core timepoint plots showed characteristic rainbow-type distributed modules interspersed by smaller co-modules. Unison of participation of ICs of clusters were similar to each other in the entire period, though replaced by a few ICs taking the temporary top tier intermittently.

B. Afferent node-voxel capacity showed two similar co-modules, i.e., one DMN/CEN/VN and another SMN/SN/AN/Unclassified. Interestingly, DAN mostly mimicked SMN/SN/AN except for a period around 150th time bin, when it adopted the feature of DMN/CEN/VN at that time bin.

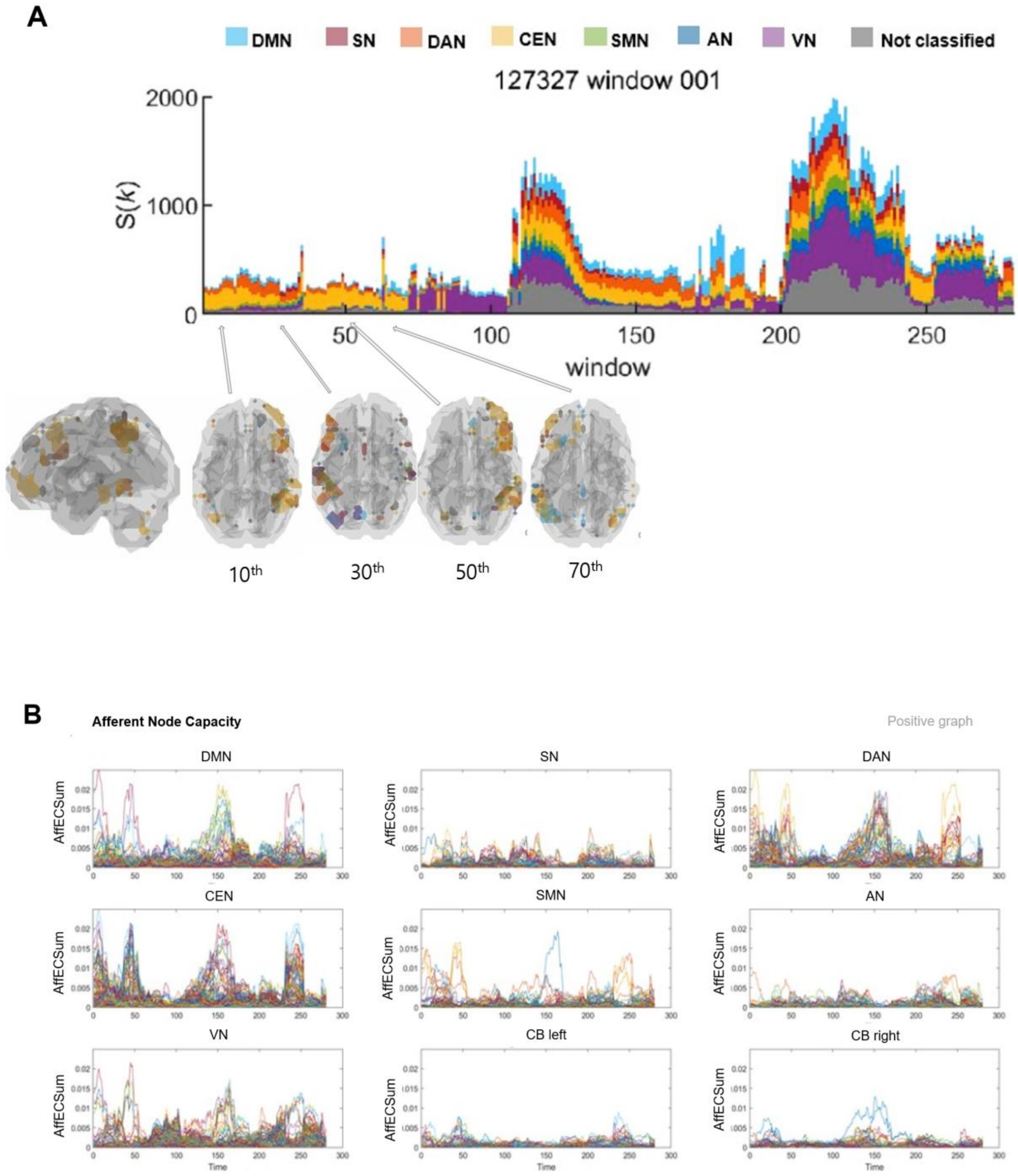
A
DMN
SN
DAN
CEN
SMN
AN
VN
Not classified
127327 window 001
S(k)
window
10th
30th
50th
70th
B
Afferent Node Capacity
Positive graph
DMN
SN
DAN
CEN
SMN
AN
VN
CB left
CB right
AffECSum
Time

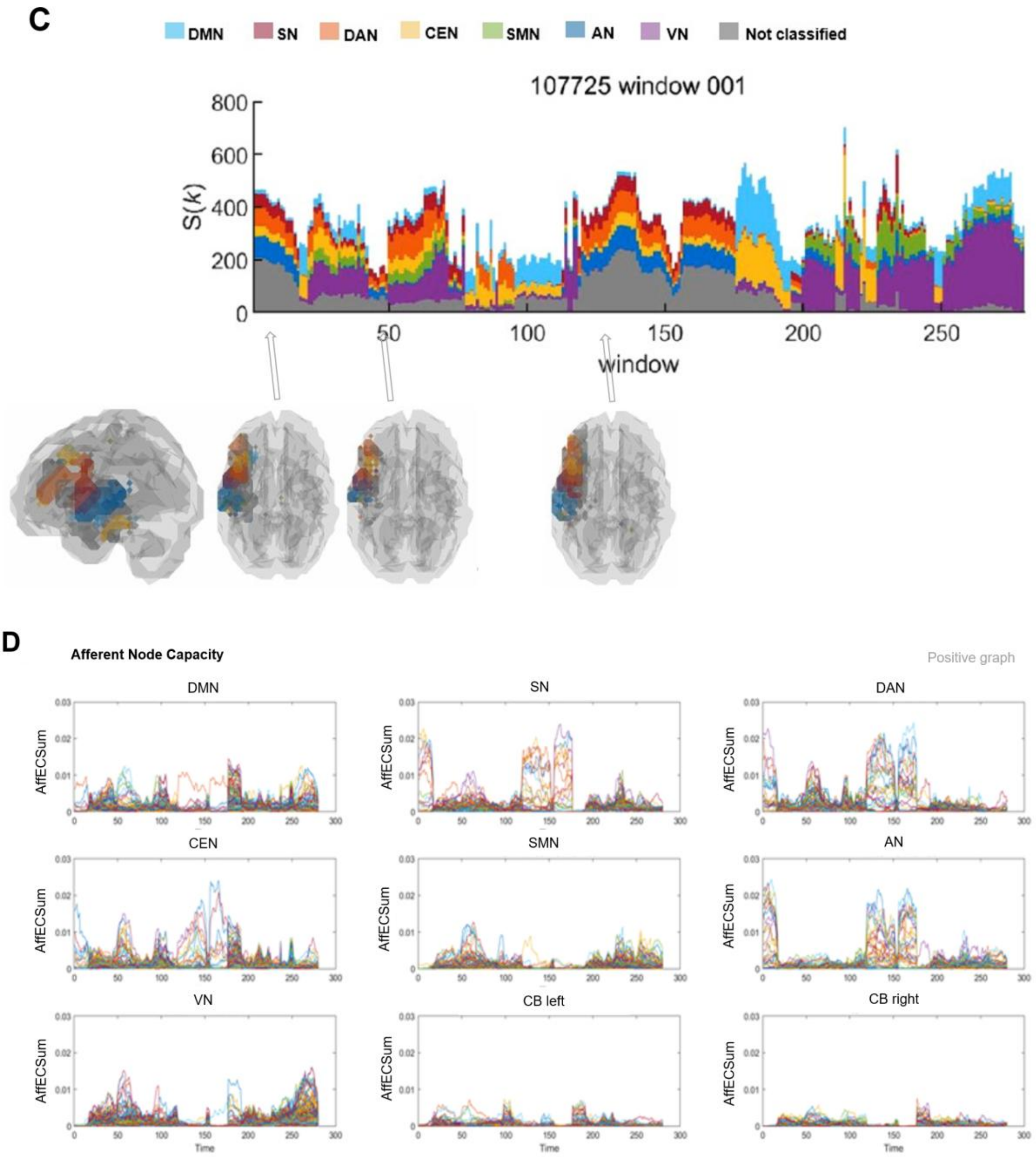
C
DMN
SN
DAN
CEN
SMN
AN
VN
Not classified
107725 window 001
S(k)
window
D
Afferent Node Capacity
Positive graph
DMN
SN
DAN
CEN
SMN
AN
VN
CB left
CB right
AffECSum
Time

E

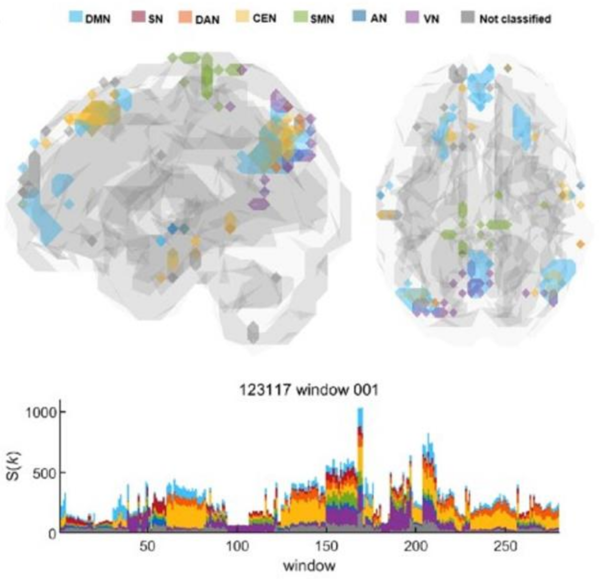

F

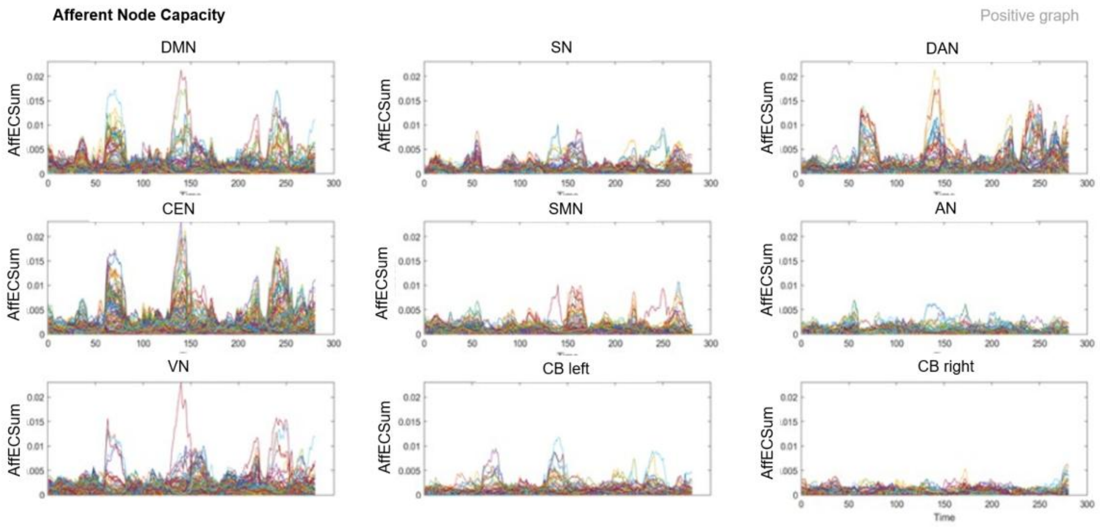

Supplementary Figure 9. Asymmetry of module composition of states in three subjects showing frontal alternating (A,B), recurrently appearing in left frontal area (C,D), and left cerebellar asymmetry (E,F) patterns.

A. This individual showed usual pattern of state transition on stacked histogram plots, but on the glass brain images, repeated alternation of right and left DAN/CEN was found.

B. Afferent node capacity showed DMN/DAN/CEN modules and independent SMN, SN, AN and the unclassified, which made rainbow-type distributed modules on k_{max}core plots at the later part of the plots.

C. In this extraordinary individual also presented in Figure 5B, k_{max}core plots showed ordinary state transition from DMN/SN/DAN/CEN/AN dominance via DMN/CEN

dominance to rainbow distributed dominance etc. On glass brain images, however, left dorsal frontal areas of DAN/CEN/SN/AN took the hierarchical supremacy three times at the start, 50th time bin and period between 120th and 170th bins until it yielded to the following DMN/CEN and then VN with other smalls.

D. On afferent node capacity plots, during the period between 120th and 170th time bins, SN/DAN/CEN/AN/Unclassified co-modules presided as dominating modules with the void VN/SMN/cerebellums

E. This individual was one of the typical examples of state transition. Peculiar finding was in the asymmetry of cerebellum. DMN/CEN, sole VN, DMN/DAN/CEN, rainbow distributed all-ICs took turns, to-and-fro with clear state transitions.

F. Afferent node-voxel capacity showed two similar co-modules, i.e., one DMN/CEN/VN and another SMN/SN/AN/Unclassified. Interestingly, DAN mostly mimicked SMN/SN/AN except for a period around 150th time bin, when it adopted the feature of DMN/CEN/VN at that time bin. Left cerebellum made modules and followed the track DMN/DAN/CEN modules, while right cerebellum did not.

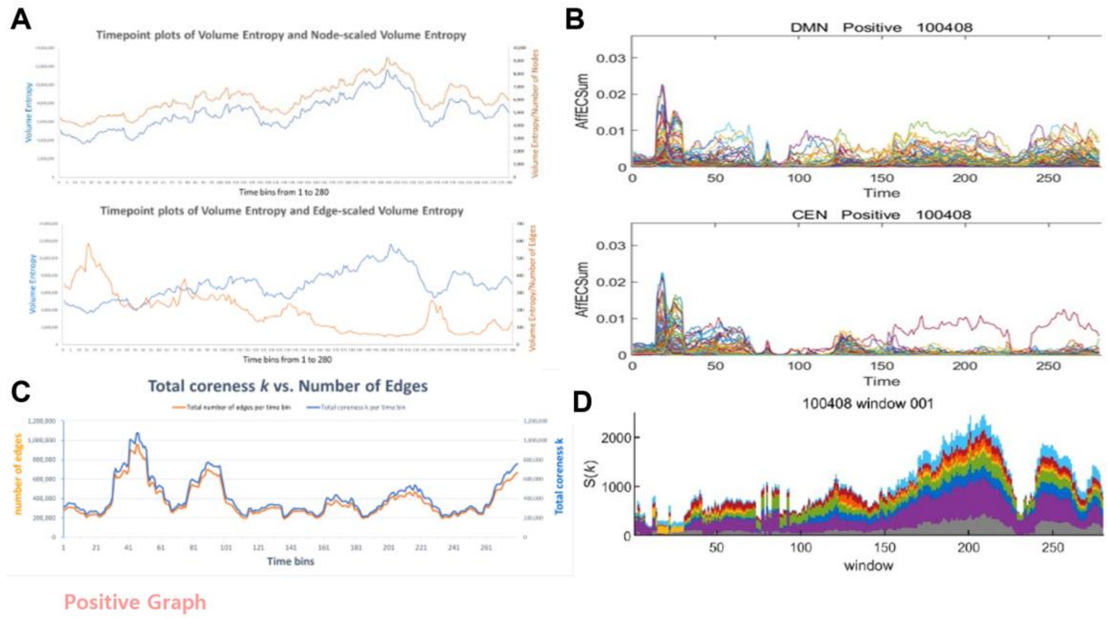

Supplementary Figure 10. Volume entropy (A) and afferent node capacity (B) with timepoint plots and their corresponding coreness k (C) and k_{max}core timepoint plots (D). All these measures were from positive graphs of an individual (#100408). A. Volume entropy was normalized using (divided by) number of nodes and the curves were exactly the same. In contrast, edge-scaled volume entropy was coarsely inverse of the original curve of volume entropy per se. B. Voxels belonging to DMN and CEN were visualized for afferent node capacity. C. Timepoint plots of total coreness k were plotted together with the total number of edges per time bins, which revealed exactly the same time course. D. Timepoint plots of k_{max}core showed initial state transitions. 10th to 30th time bins showed dominance of voxels belonging to DMN/CEN, which was mimicked by the module formation of and exchange on the afferent node capacity.

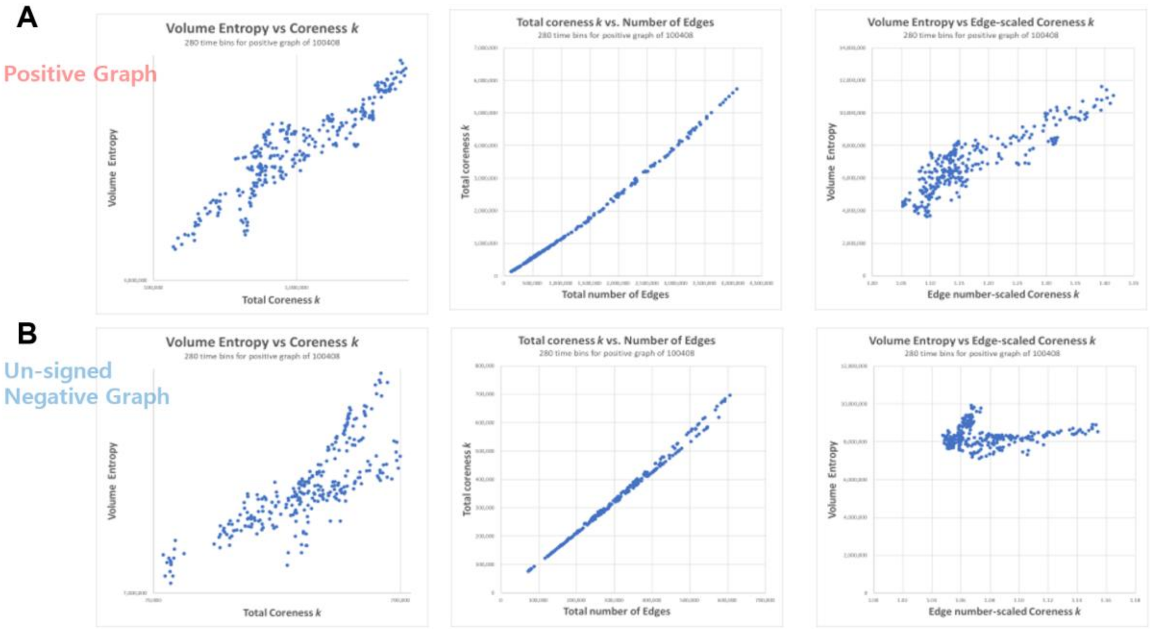

Supplementary Figure 11. The relationships between the volume entropy and total coreness k of the time bins of the positive and negative graphs of the individuals are presented in Suppl. Figure 10.

First column shows the apparent correlation between volume entropy and total coreness k per time bin graphs. It is noted that those two measures are global ones for the graph as a total. On the second column, the expected 1:1 relationship between total edge number and total coreness k, and thus on the third column, relationships between volume entropy and edge-scaled total coreness k were shown. Correlations seemed to exist between volume entropy and total coreness k on the first column plots, however, on the third column especially in negative graphs, the relationship became scattered and were then assumed to have depended on the confounding effect of varying edge numbers.

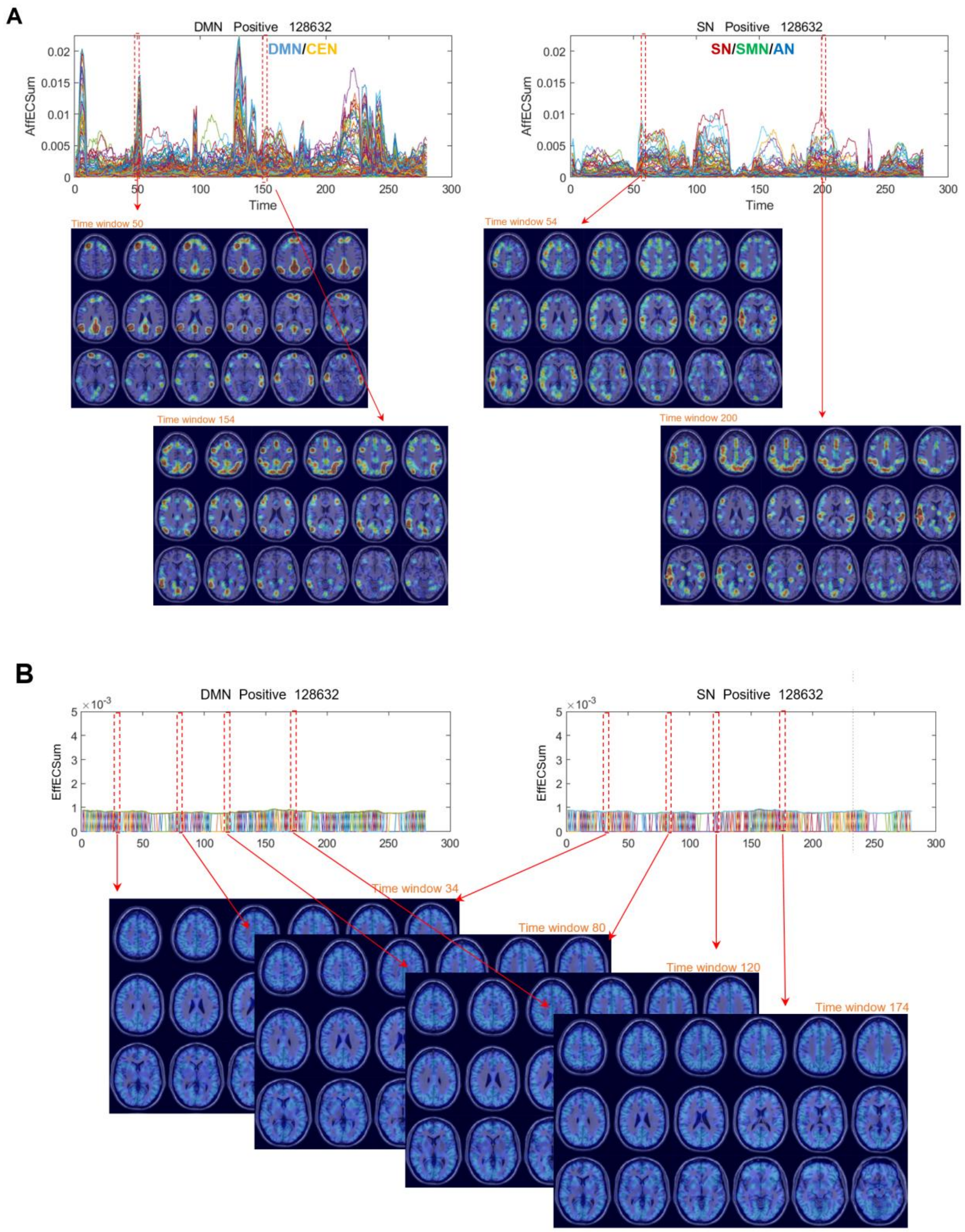

Supplementary Figure 12. Example of voxels/IC composition timepoint plots and their

afferent and efferent node capacity animation maps of positive graphs in an individual (#128632). The snapshot images of animation maps were presented for immediate visual recognition.

A. Timepoint plots of afferent node capacity of the positive graph of an individual. Snapshots of animation of afferent node capacity accompanied the timepoint plots at 50th and 154th time bins for voxels of DMN and at 54th and 200th time bins for voxels of SN.

B. Timepoint plots of efferent node capacity of the positive graphs were presented as a line with markers. Snapshots of animation of efferent node capacity were attached at 34th, 80th, 120th, and 174th time bins. It was noted that efferent node capacity was homogeneous with flickering all over the gray matter voxels, and thus timepoint plots were unremarkable even not to make any discernable collective modules.

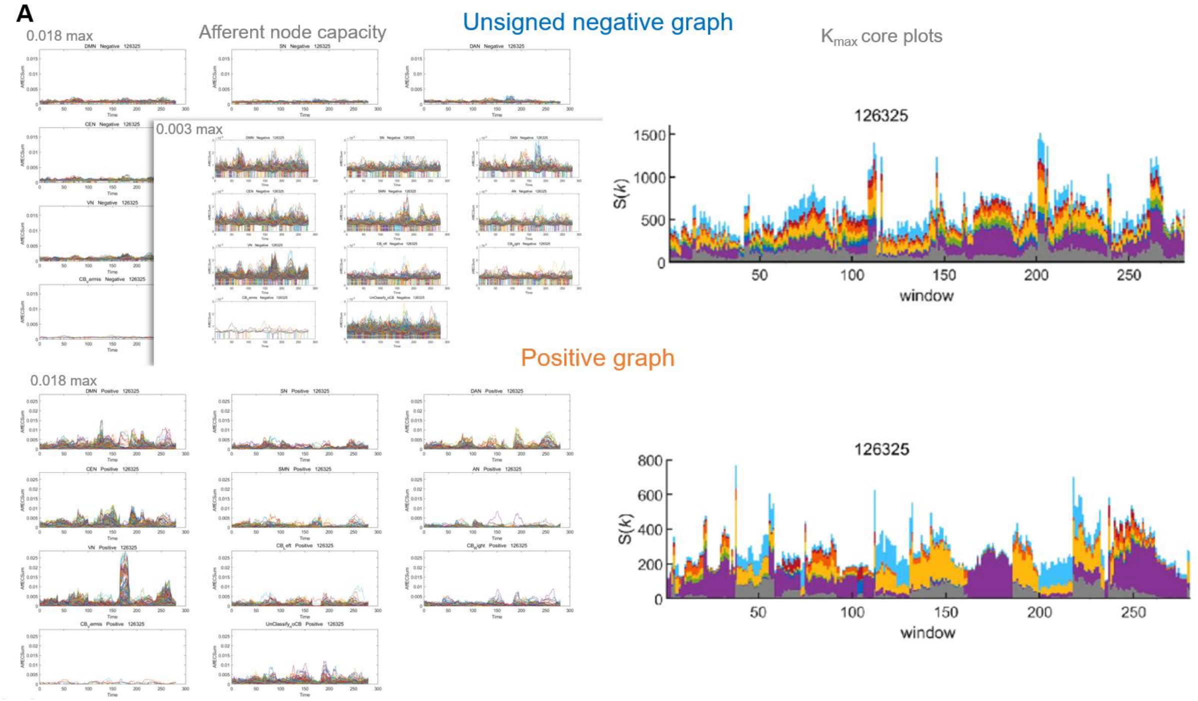

A
0.018 max
Afferent node capacity
Unsigned negative graph
K_max core plots
0.003 max
126325
S(k)
window
Positive graph
0.018 max
126325
S(k)
window

B

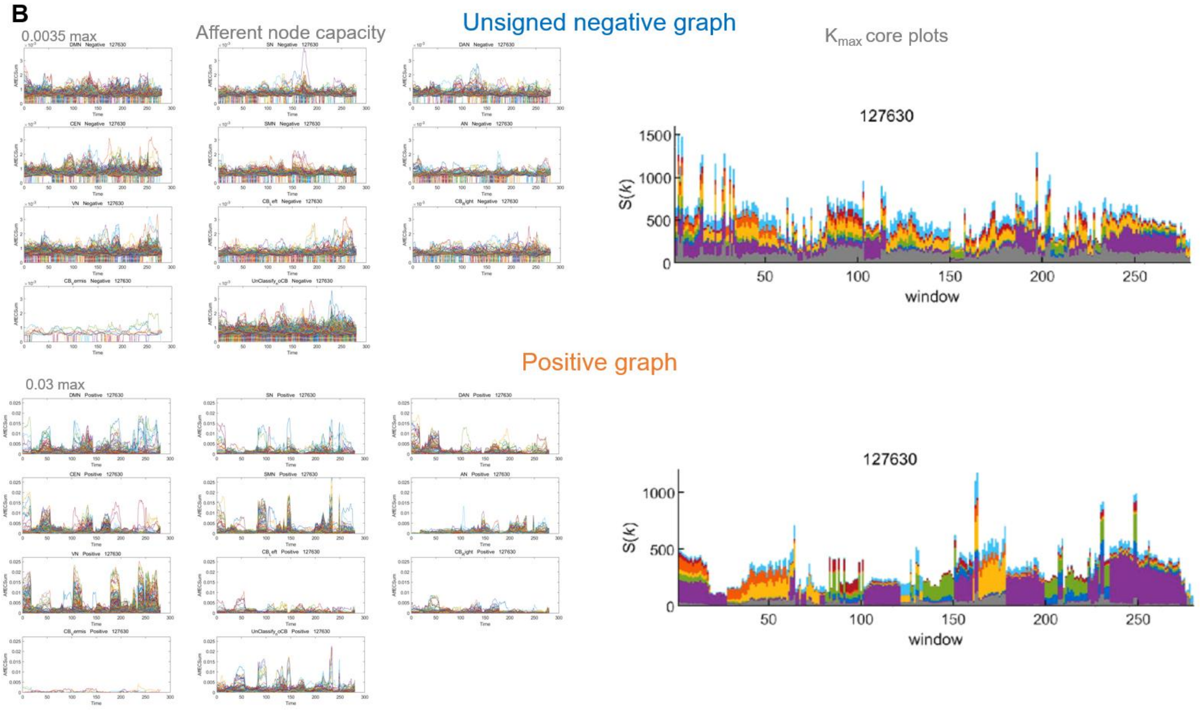
0.0035 max
Afferent node capacity
Unsigned negative graph
K_{max} core plots
127630
S(k)
window
Positive graph
0.03 max
127630
S(k)
window

C

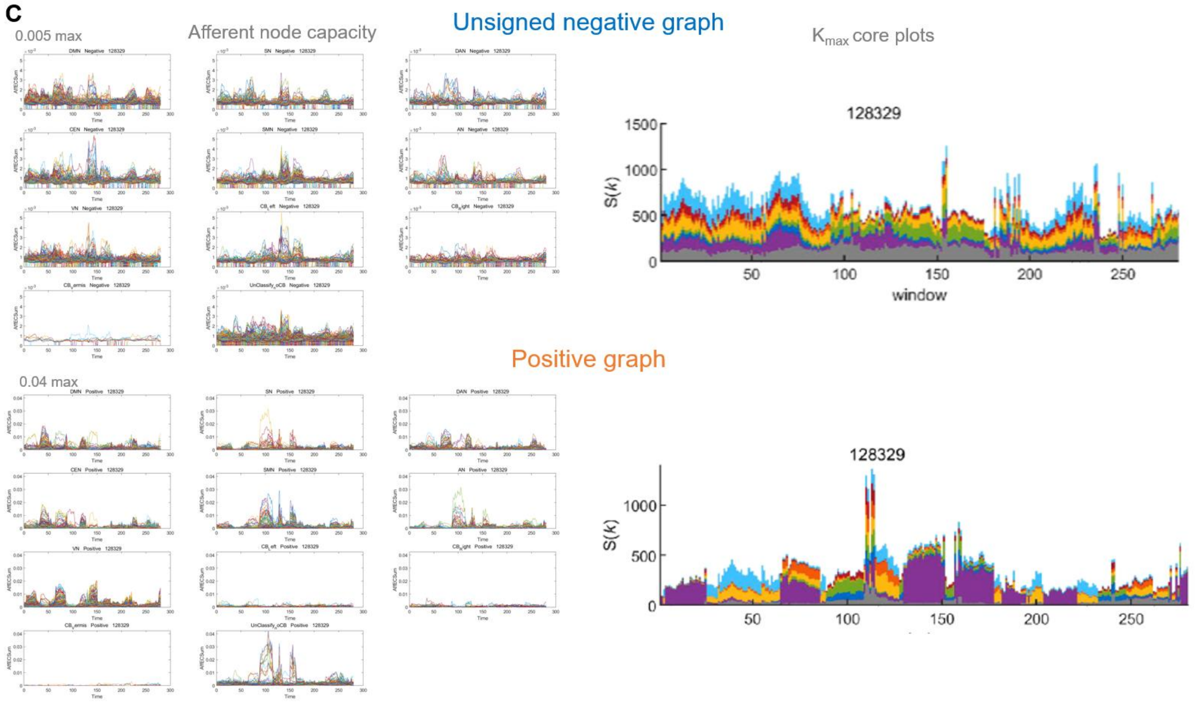
0.005 max
Afferent node capacity
Unsigned negative graph
K_{max} core plots
128329
S(k)
window
Positive graph
0.04 max
128329
S(k)

D

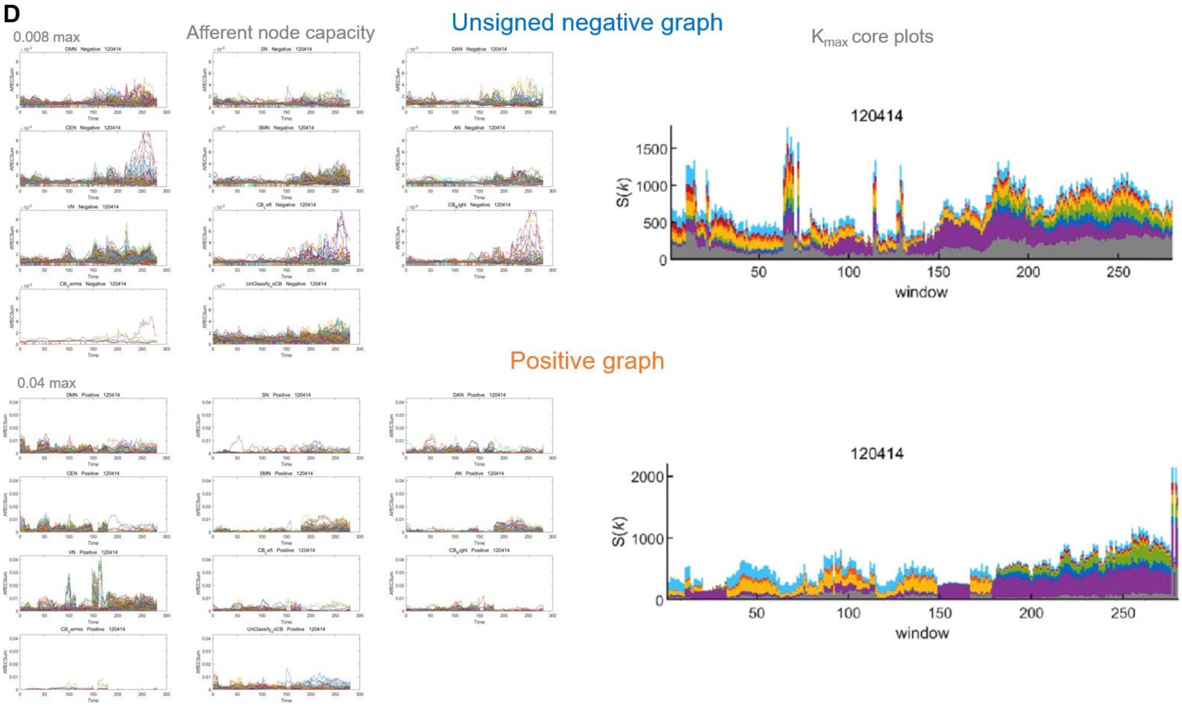
0.008 max
Afferent node capacity
Unsigned negative graph
K_{max} core plots
120414
S(k)
window
Positive graph
0.04 max
120414
S(k)
window

E

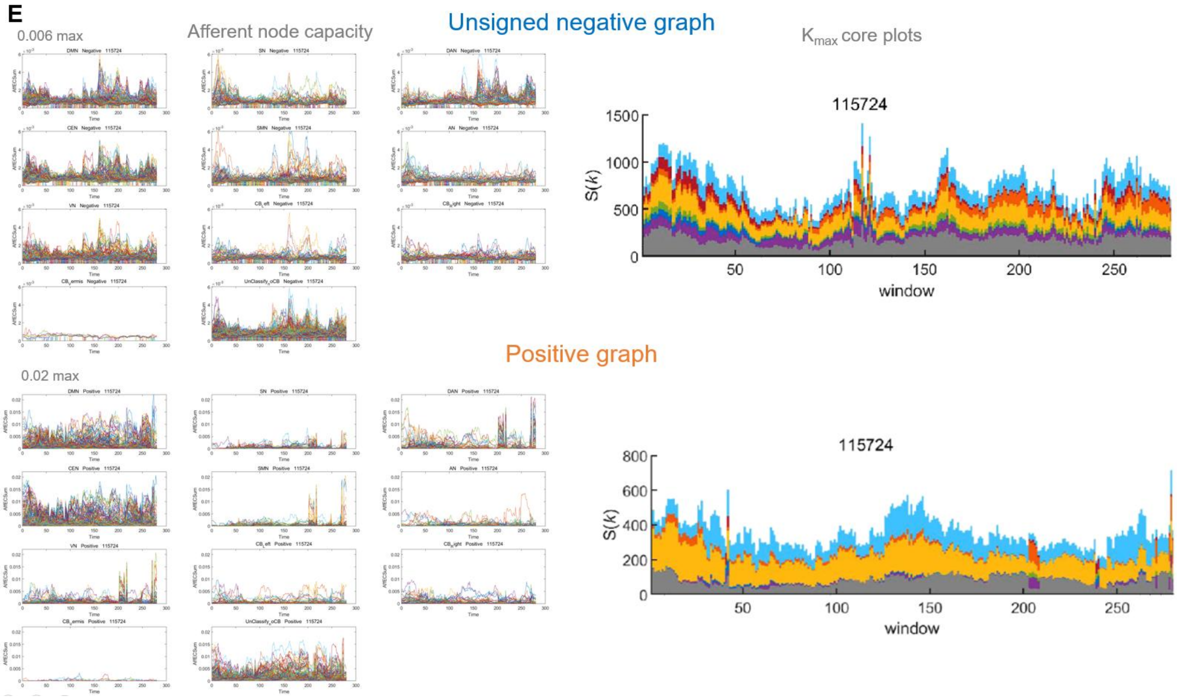
0.006 max
Afferent node capacity
Unsigned negative graph
Kmax core plots
115724
S(k)
window
Positive graph
0.02 max
115724
S(k)
window

F

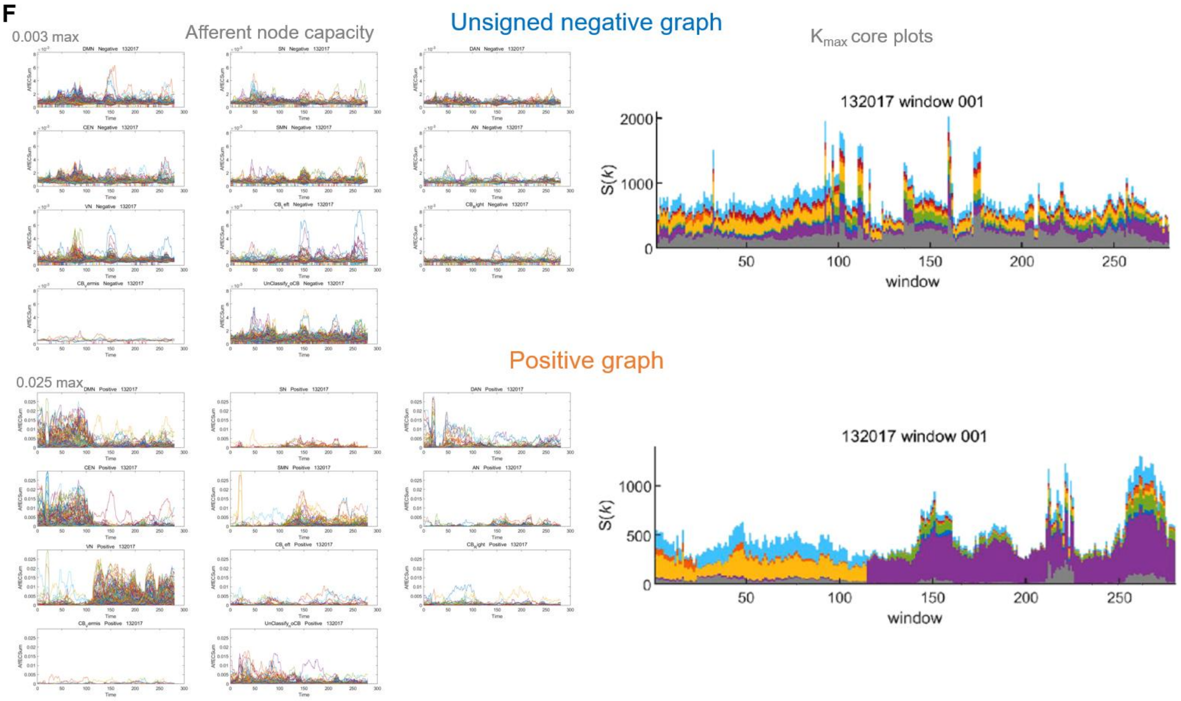
0.003 max
Afferent node capacity
Unsigned negative graph
Kmax core plots
132017 window 001
S(k)
window
Positive graph
0.025 max
132017 window 001
S(k)
window

G

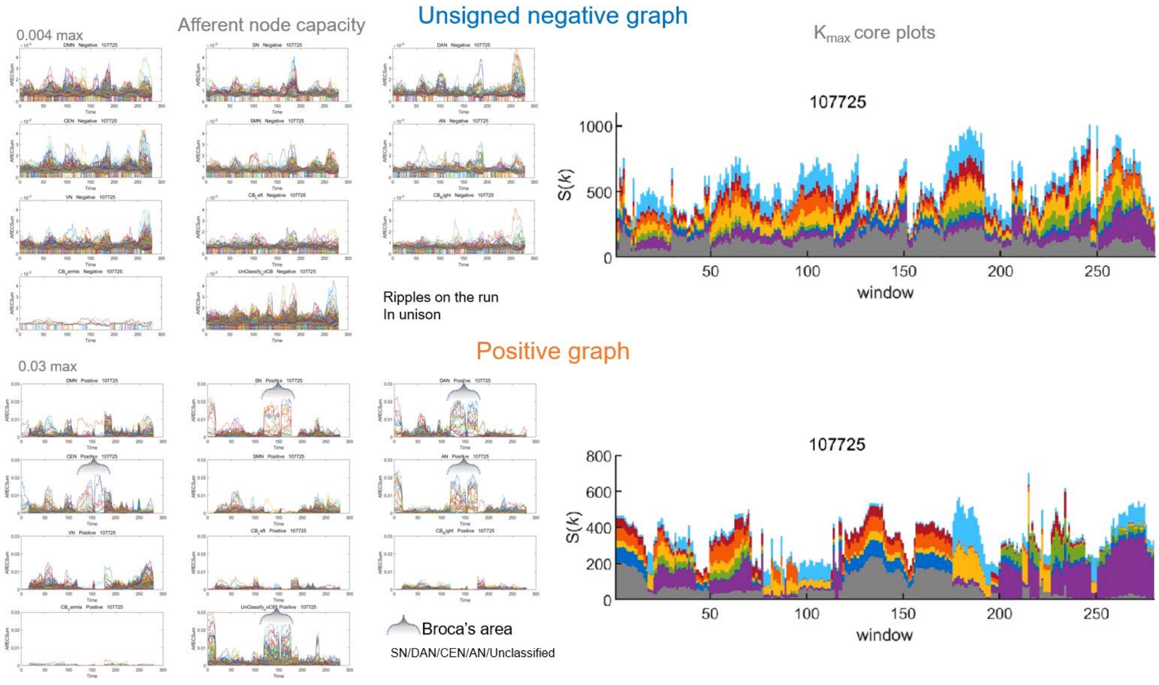

H

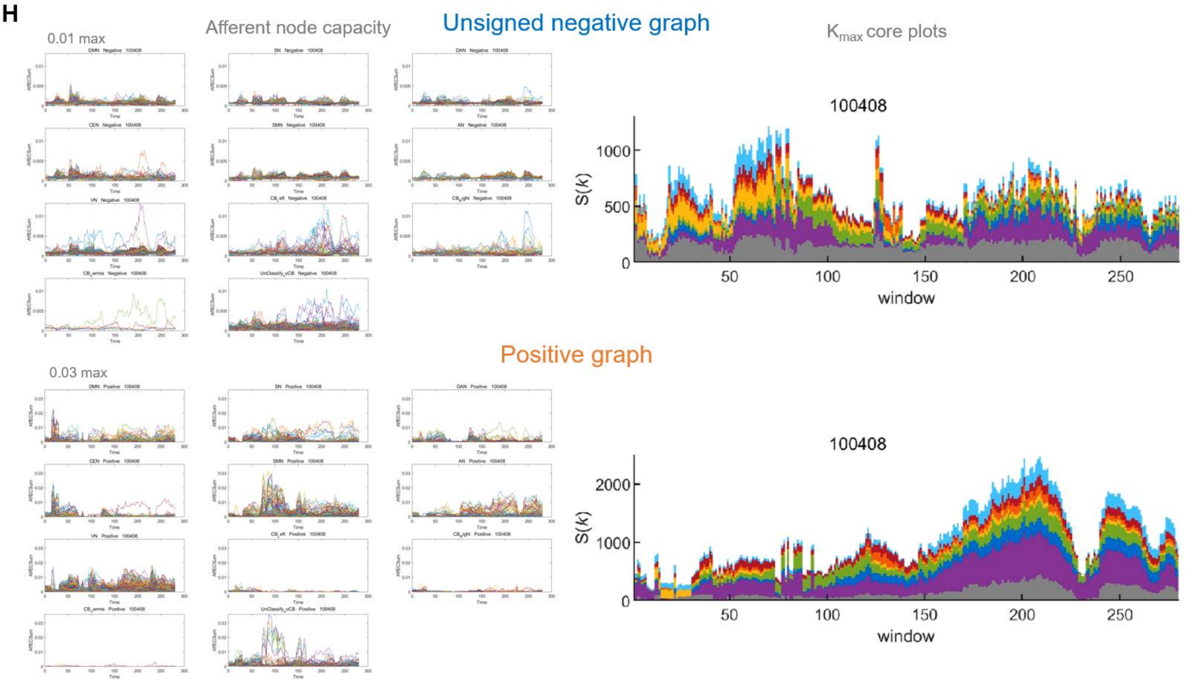

Supplementary Figure 13. Back-to-back representation of afferent node capacity of voxels/IC timepoint plots of unsigned negative graphs and their corresponding positive

graphs in representative individuals, followed by their matching k_{max}core stacked histogram timepoint plots. In the following 8 example showcases, in negative graphs unlike in positive graphs, afferent node capacity plots did rarely show modules nor module exchange.

A. Afferent edge capacity of negative graphs were much smaller ($1/6^{th}$) than those of positive graphs. Unlike afferent node capacity of positive graphs, those of negative graphs did not show evidence of module formation and when the maximum of the ordinate was lowered from 0.018 to 0.003, ripples of collective voxel trajectories rose and fell just in the small and fragmented waves in unison among the ICs. On k_{max}core stacked histogram plots, state transition was not prominent in negative graphs, which was prominent in positive graphs.

B. Height of ripples of afferent node capacity of negative graphs was $1/10^{th}$ of that of positive graphs. Once amplified, the ripples and loose threads were noted in negative graphs. A few state transitions were there on the stacked histogram plots of the negative graphs but not as much explicitly and frequently as those of the positive graphs.

C. Timepoint plots of afferent node capacity showed ripples with much lower values ($1/9^{th}$) in negative graphs than in positive graphs. In contrast, stacked histogram plots of k_{max}core showed at least two state transition at 100th time bin and 180th time bin. For these module exchanges of negative graphs, all the ICs constituted the k_{max}core at 100th time bin until DMN voxels left and SMN voxels joined and stayed till 180th time bin. Then the voxels/ICs composition returned to the previous one.

D. In this case, $1/5^{th}$ of height (compared to that of positive graph) of afferent node capacity of negative graph, was dedicated to later surge of trajectory of DMN/CEN/DAN/SMN/AN and cerebellums. Stacked histogram plots of negative graph also showed different pattern between the first and the second halves on k_{max}core stacked histogram plots. Afferent node capacity and stacked histogram of positive graphs showed the concordant changes of DMN/CEN and VN/SMN/AN modules.

E. 1/3 height (compared to that of positive graph) of afferent node capacity of negative graph showed ripples in unison, which was also manifest in k_{max}core stacked histogram of negative graph. Interestingly, afferent node capacity of positive graph was dominated by the participation of voxels of DMN/CEN/unclassified. This was also corroborated by the same findings of k_{max}core stacked histogram plots of positive graph.

F. $1/8^{th}$ height (compared to that of positive graph) of afferent node capacity of negative graph showed ripples in unison and threads. Stacked histogram plots of k_{max}core could be divided to 3 to 5 segments with different voxels/IC composition in negative graph. In contrast, afferent node capacity and k_{max}core results were concordant in that the DMN/CEN first and then VN major in the following. Modules of longer duration on afferent node capacity plots were compatible with the top tier voxels/IC on stacked histogram plots in positive graphs.

G. While $1/8^{th}$ height (compared to that of positive graph) of afferent node capacity showed ripples in unison and matching top tier voxels on the stacked histogram plots of k_{max}core in

negative graphs, afferent node capacity plots showed typical module formation and exchange in positive graph. Stacked histogram of k_{max}core also showed typical state transition in positive graphs. This case was the one in Figure 5B and Suppl. Figure 13CD. Afferent node capacity matched k_{max}core of negative graph as well as in positive graph. Unique in this case was the finding that we needed MRI-overlayed voxel coreness k animation plot to find the Broca area and asymmetry of voxels/IC contribution to modules formation/exchange.

H. 1/3th height (compared to that of positive graph) of afferent node capacity of negative graph showed unimpressive small waves but with much loose threads in VN, left and right cerebellum and the unclassified. Stacked histogram of k_{max}core of negative graph also showed progressive changes of IC composition but not with exact state transition. In this case, the stacked histogram of k_{max}core of positive graph also showed homogenous progress of totally one state except for the initial transition to DMN/CEN and then back to 'VN and all the others' state. Afferent node capacity plots of positive graph showed very early DMN/CEN module and then the modules were scattered over VN/SMN/SN and AN.

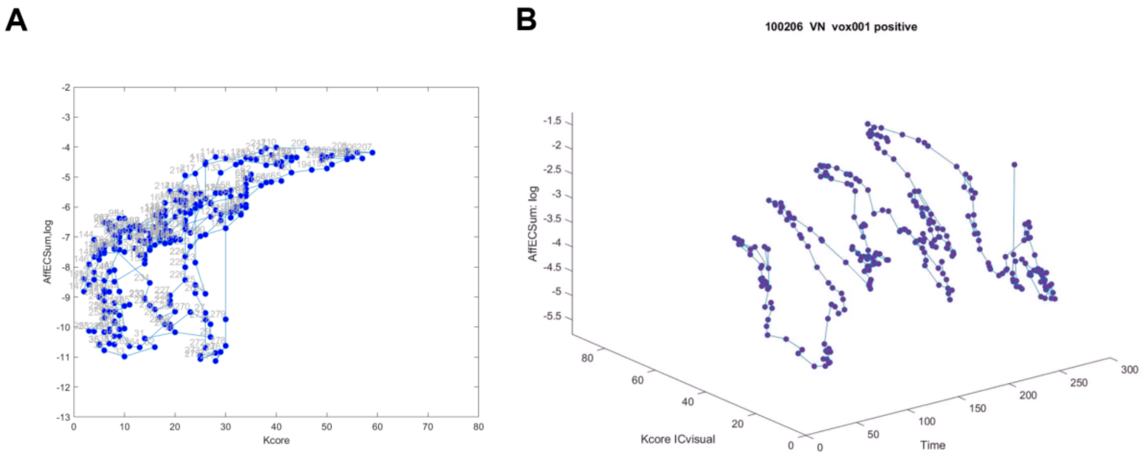

Supplementary Figure 14. Trajectory tracing of a voxel along the time-bin progress of its own coreness k values and afferent node capacity. This was the snapshot of animation plots showing all the voxels from an IC (VN, n=247 voxels). It is to be remarked that coreness k value was derived from k core percolation of undirected graph of 5,937 or 1,489 voxels data. For this comparison, we performed another k core percolation for positive graph of #100206 using 10x10x10 mm^3 matrix (voxel number of 1,489) with the same threshold of 0.65. 247 voxels were given their coreness k value and afferent node capacity derived from positive graph. 280 time-bin data was plotted in two ways.

A. Logarithmic representation on the ordinate and linear one on the abscissa box was filled with 280 time-bins data of voxel 1 among 247 voxels. In fact, this data was also in animation plot in which we could see the pattern of all the 247 voxels. On the readout of animation plot, frog-like expansion and shrinkage was observed both horizontally and in up-and-down fashion.

B. 280 time-bin data were expanded with the help of Matlab. And animation plot was reviewed. We now could see the collective frog-like motions of each time bin altogether to reach the top tier of hierarchy with the help of afferent node capacity of each voxel at that moment.

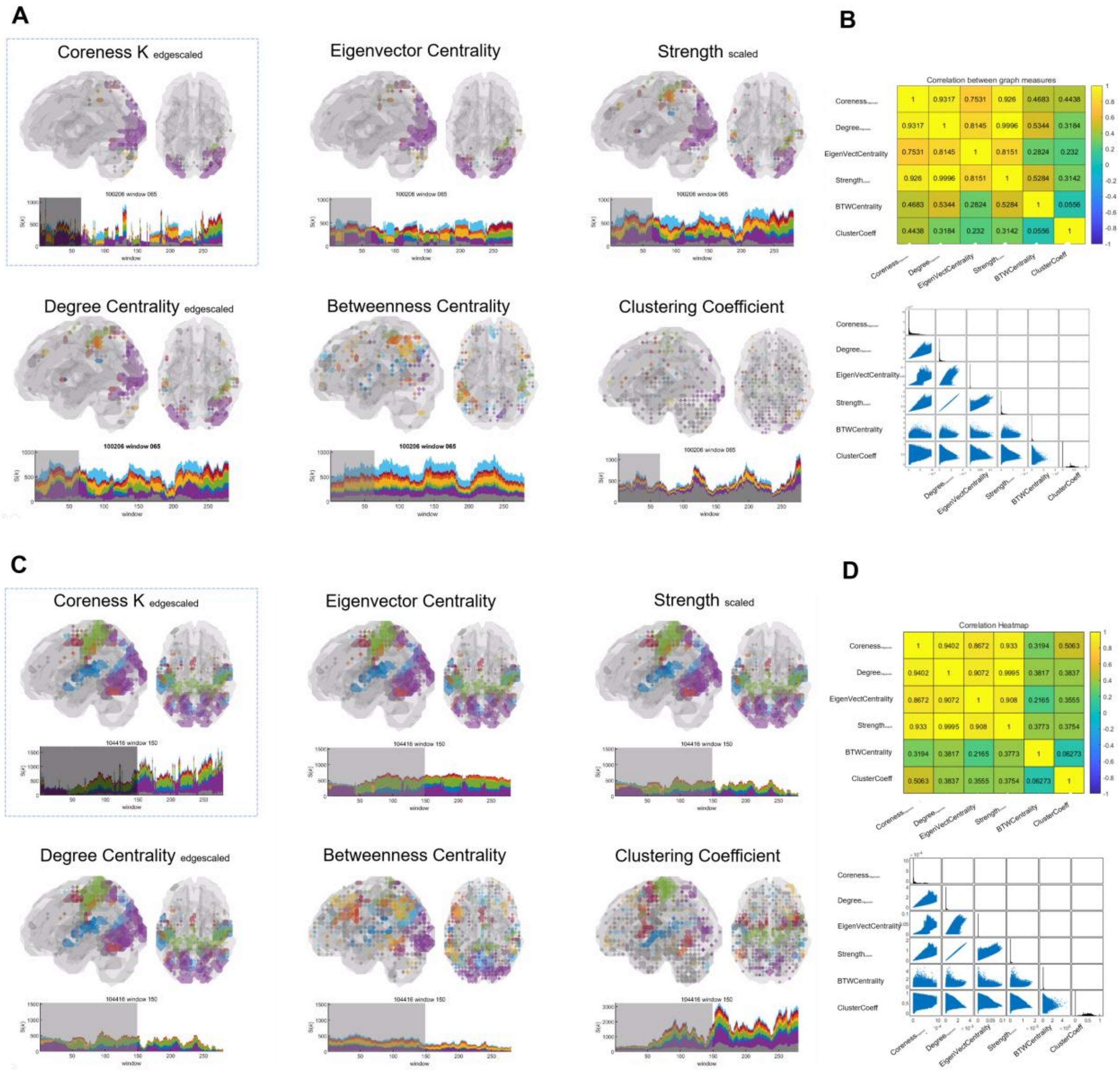

Supplementary Figure 15. Glass brain animation plots and stacked histogram timepoint plots of k_{max}core voxels and other graph metrics

A. Glass brain images and stacked histograms along 280 time bins of the k_{max}core, eigenvector centrality, strength, degree centrality, betweenness centrality, and clustering coefficient in the individual subject brain. Voxels belonging to IC**s** were similar in k_{max}core, eigenvector centrality, strength, and degree centrality. However, In the stacked histogram, the time-dependent changes in the k-core are more pronounced in terms of state transitions compared to other graph metrics. The transitions observed in the stacked histograms of other graph metrics appear smoothed compared to those seen in the k-core. B. Correlation between each pair of graph measures showed in heatmap and plotmatrix. C. In another individual subject as well, the patterns observed in the glass brain are similar to those seen in degree,

strength, and eigenvector, however the state transitions in the stacked histogram of k_{max}core are more pronounced. D. Additionally, these four graphs metrics show a high correlation, as can also be seen in the heatmap and plot matrix.
